# The causal relationship between the human gut microbiota and pyogenic arthritis: a Mendelian randomization study

**DOI:** 10.3389/fcimb.2024.1452480

**Published:** 2024-11-26

**Authors:** Boliang Bai, Longfei Luo, Feng Yao, Qian Sun, Xingguang Chen, Wen Zheng, Lang Jiang, Xiaodong Wang, Guanghao Su

**Affiliations:** ^1^ Department of Orthopedics, Affiliated Children’s Hospital of Soochow University, Suzhou, China; ^2^ Department of Bioinformatics, Center for Systems Biology, School of Biology and Basic Medical Sciences, Suzhou Medical College of Soochow University, Suzhou, China; ^3^ Department of Orthopedics, The Second Affiliated Hospital of Jiaxing University, Jiaxing, China

**Keywords:** pyogenic arthritis, gut microbiota, Mendelian randomization, gut microbiota metabolic pathways, causal effect

## Abstract

**Background:**

Recent studies have indicated the role of the gut microbiota in the progression of osteoarticular diseases, however, the causal relationship between the gut microbiota and pyogenic arthritis remains unclear. There is also a lack of theoretical basis for the application of the gut microbiota in the treatment of pyogenic arthritis.

**Methods:**

In our study, we utilized the largest genome-wide association study (GWAS) data from the MiBioGen Consortium involving 13,400 participants and extracted summary statistical data of the microbiota metabolic pathways of 7,738 participants of European descent from the Dutch Microbiome Project (DMP) The data of pyogenic arthritis were derived from the FinnGen R10 database, including 1,086 patients and 147,221 controls. We employed the two-sample Mendelian randomization approach to investigate the causal association between the gut microbiota and pyogenic arthritis. Our methods comprised inverse variance weighting, Mendelian Randomization Egger regression, weighted median, and weighted modal methods. Subsequently, polygenic and heterogeneity analyses were conducted.

**Results:**

At the class level, β-proteobacteria is positively correlated with the risk of pyogenic arthritis. At the order level, Burkholderia is positively associated with the disease. At the genus level, the unclassified genus of Sutterellaceae is positively correlated with the disease, while the unnamed genus of Lachnospiraceae, Rothia, and the unnamed genus of Erysipelotrichaceae are negatively correlated with the disease. In addition, Faecalibacterium and Finegoldia are also negatively correlated with the disease. Sensitivity analysis did not show any abnormal evidence.

**Conclusion:**

This study indicates that β-proteobacteria, Burkholderiales, and the unclassified genus of Sutterellaceae are associated with an increased risk of the disease, while the unnamed genus of Lachnospiraceae, Rothia, the unnamed genus of Erysipelotrichaceae, Faecalibacterium, and Finegoldia are related to a reduced risk. Future studies are needed to elucidate the specific mechanisms by which these specific bacterial groups affect pyogenic arthritis.

## 1 Introduction

Pyogenic arthritis (PA) is a severe joint emergency, if not controlled in time, it will lead to cartilage destruction, irreversible damage to the concerned joints and arthritis, and even the occurrence of acute septic shock. The incidence of PA is 2-6/100,000 people (Sharoff et al., 2024). It is commonly caused by infection with Staphylococcus aureus, and with the application of advanced diagnostic methods such as real-time polymerase chain reaction(PCR), the cases of PA caused by Kingella kingae and suppurative streptococcus are gradually increasing (Iseri Nepesov et al., 2024). At present, the therapy for osteoarticular diseases include drug and non-drug plans, for example: healthier lifestyle, supplementing proteins, calcium and vitamin D, and maintaining reasonable weight (Papageorgiou and Biver, 2021). Although PA has been studied for a long time, there have not been many changes in the treatment protocol in nearly 30 years, and about 50% of patients have left irreversible joint sequelae. These sequelae include: joint pain and limited movement, joint stiffness, joint deformity, cartilage damage, bone destruction, chronic arthritis, etc (Jin, 2024). So, this clinical problem is urgently awaiting to be solved. The role of the gut microbiota (GM) in osteoarticular health and diseases is gradually being focused on, and they may affect the human body through themselves or their metabolites.

The microorganisms in the human gastrointestinal tract, known as the GM (most of which are bacteria, as well as viruses, fungi, and other microorganisms), co-inhabit the human body together (Papageorgiou and Biver, 2021). Research has demonstrated that the GM participates in regulating various physiological processes in the human body (Dominguez-Bello et al., 2019). Meanwhile, it is related to many human diseases, such as obesity, cardiovascular diseases, inflammatory bowel disease, cancer, diabetes, and depression (Fonseca et al., 2024; Wang et al., 2024), and it also exerts an important role in inflammatory joints (Gao et al., 2024; Shan et al., 2024; Yao et al., 2024). Although current observational studies have provided valuable insights into the changes in the microbiota of PA patients, determining causal relationships still remains challenging. The observational data influenced by multiple confounding factors such as environmental factors, drug effects, and dietary habits are not sufficient to ultimately determine whether the changes in the microbiota are causally related to PA (Cafaro et al., 2024).

Mendelian randomization (MR) is a statistical method used to infer the causality between exposure factors and outcomes, usually by using genetic variation as an instrumental variable(IV) (Li et al., 2024). MR utilizes the random classification of alleles in the genetic process to bypass confounding factors and reverse causality, so it is similar to randomized controlled trial(RCT) and has a relatively strong evidence level. Although there are an increasing number of studies verifying the association between the GM and diseases through MR (Elhage et al., 2024; Lou et al., 2024; Ni Lochlainn et al., 2024), the causality between the GM and the risk of PA onset has not been studied yet.

In this research, we adopt the approach of Mendelian randomization to explore the potential causality between the gm and PA, offering assistance for the pathogenesis of the disease and the treatment strategy.

## 2 Materials and methods

### 2.1 Data sources

From a whole-genome association study (GWAS) of the MiBioGen Alliance, a total of 18,340 multiple participants (85% of European descent) were extracted with summary statistics of the abundance of the GM (Kurilshikov et al., 2021), through 16S ribosomal ribonucleic acid (rRNA) gene sequencing analysis, there were a total of 211 taxa: 9 phyla, 16 classes, 20 orders, 35 families, and 131 genera. Summary statistics of the microbiota metabolic pathways of 7,738 European-descent participants were obtained from the Dutch Microbiome Project (DMP) (Lopera-Maya et al., 2022), including 205 bacterial metabolic pathways (**Supplementary Table S1**). PA data comes from FinnGen R10 project database (https://www.finngen.fi/en/) finn-b-M13_PYOGARTH, including 1,086 cases and 14,7221 controls, with a total of 16,380,139 available Single nucleotide polymorphisms (SNPs).

### 2.2 Selection of the IV

The IVs used in the research need to fulfill three assumptions: (1) There is a substantial correlation between the SNP and the exposure aspects; (2) The SNP is mutually independent from the confounding aspects; (3) The SNP can only exert an influence on the outcome through the exposure aspects., the genetic variation does not influence the outcome.SNPs refer to the diversity of DNA sequence caused by a single nucleotide variation at the genomic level,and are not affected by acquired confounding factors (Larsson et al., 2023). So compared with the random allocation of exposure in traditional random controlled trials, using SNP as a tool variable has advantages (Ference et al., 2021). So we chose SNPs as IVs.Firstly, we performed a correlation analysis to determine whether there was an association between SNPs and GMs as well as PA, along with the strength of this association. Initially, we set the significance level of SNPs related to GM and PA at p<1×10^-8^. However, after applying this setting to the GM data, we found that the number of available SNPs in the result was too few to conduct further research. Thus, we adjusted the significance level of the GM data and set it to p<1×10^-5^; Additionally, in order to guarantee the independence of SNPs and avoid linkage disequilibrium (LD) bias (Burgess et al., 2017), We set the LD level to r^2^ < 0.001 with a distance of 10,000 kb. Next, we removed weak instrumental variables through the F value (Burgess and Thompson, 2011) to ensure that the selected SNPs had sufficient strength to accurately infer causal relationships. We retained the SNPs with F > 10. F = R^2^×(N-1-i)/(1-R^2^)×i, N is the sample size and i is the number of effective SNPs, R^2^ = 2 × EAF × (1 - EAF) × β^2^ (i < 10) or R^2^ = 2 × EAF × (1 - EAF) × β^2^/[(2 × EAF × (1 - EAF) × β^2^) + (2 × EAF × (1 - EAF) × N × SE^2)^] (i ≥ 10) (Rogne et al., 2024), EAF is the allele frequency of the exposure factor, β is the effect size of genetic variation on the exposure factor and SE is the standard error.Finally, we manually removed SNPs related to confounding factors through the online phenotype scanning tool to reduce the interference of confounding factors on causal inference.Through extensive literature review and in-depth discussions with experts in the field, we conducted a thorough review of all the selected SNPs and found that none of them could be determined to be related to confounding factors (see **Supplementary Tables S1, S2**). To further verify the reliability of the process of removing confounding factors, we performed a sensitivity analysis. We found that the results remained basically stable, indicating that these SNPs are reliable.

### 2.3 Methods of statistical analysis

In order to study the causality between the GM and PA, we mainly use the inverse variance weighted (IVW) method for analysis, and the other four methods (MR. Egger, weighted median, Simple mode, and weighted mode) for supplementation. When IVW P < 0.05, it is considered that the result is significant.

**Figure 1:**
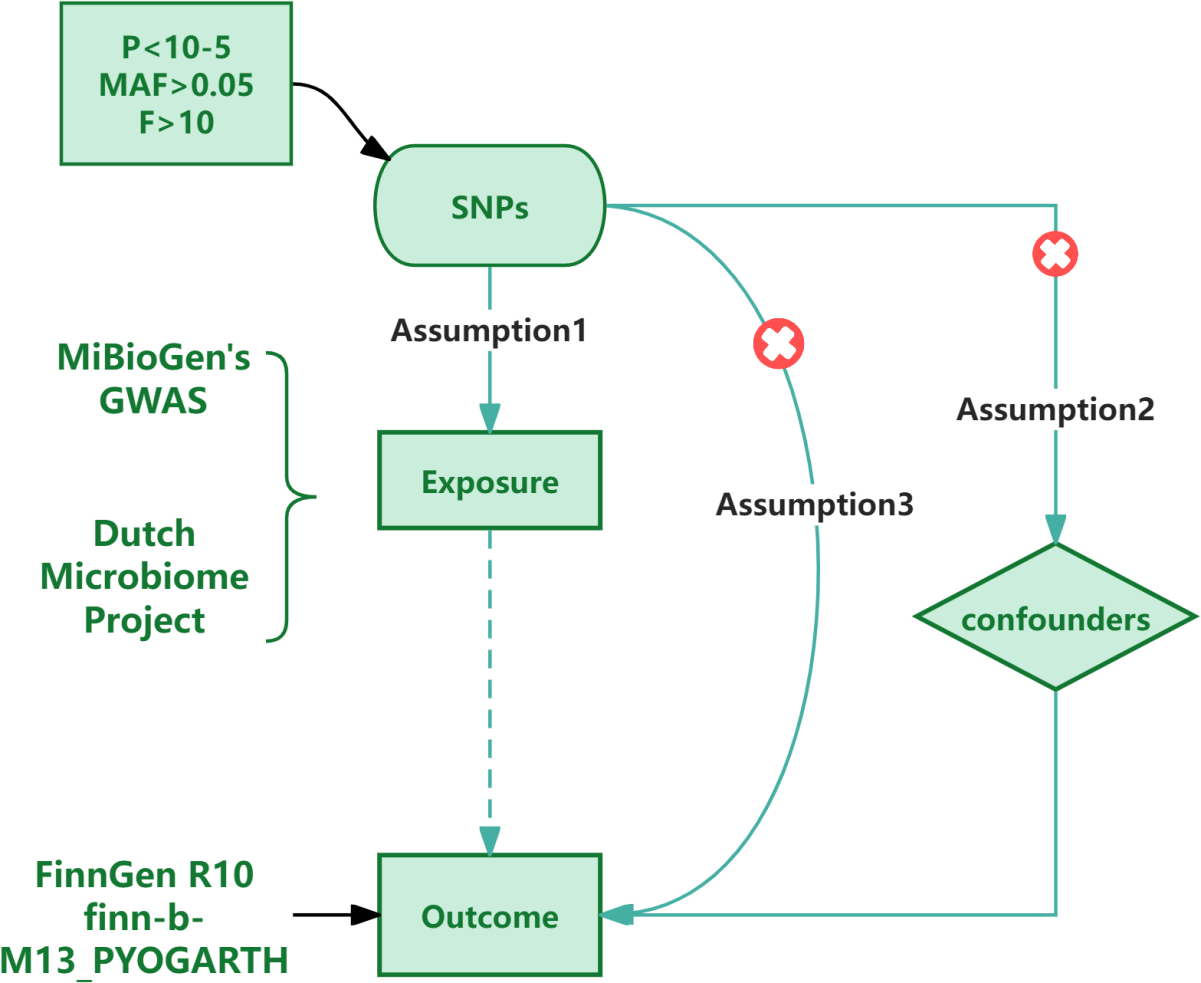
Flowchart of this Mendelian randomization study.

In addition, in order to maintain the stability of the results, we also carried out tests for heterogeneity and pleiotropy. We use Cochran’s Q test (Burgess and Thompson, 2017; Hao et al., 2024) to verify the heterogeneity among the relevant SNPs, and consider that the heterogeneity has no effect on the results when P > 0.05.In the research, we used MR Egger regression to verify the horizontal pleiotropy of the results, and considered that there was no horizontal pleiotropy when p > 0.05. The analysis software uses Rstudio 4.3.3, and the analysis package uses Two Sample MR and PhenoScanner. The process is shown in **Figure 1**.

## 3 Results

**Figure 2:**
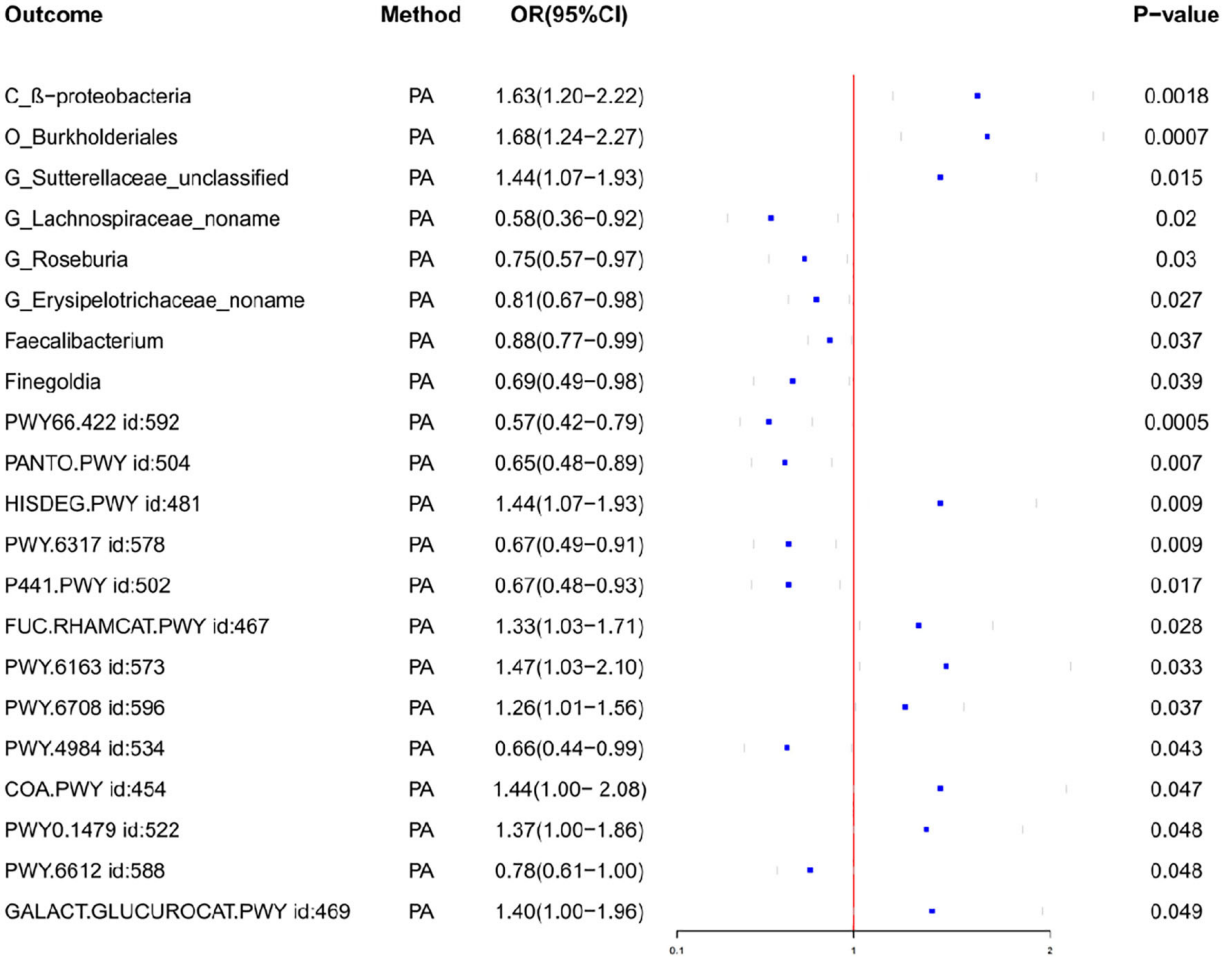
Forest plot of the associations between gut microbiota and Pathway with the risk of pyogenic arthritis.

**Figure 3:**
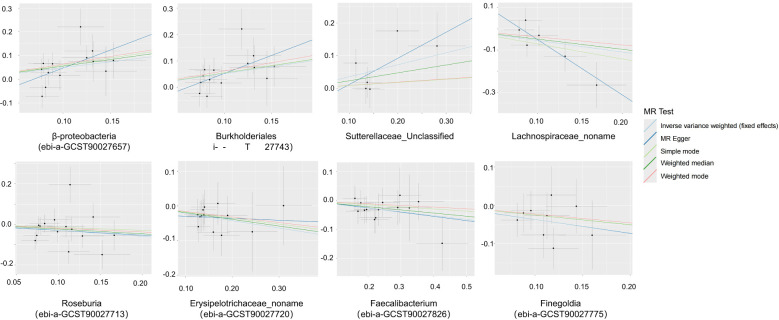
Scatter plots of the causal association between the gut microbiota and pyogenic arthritis.

Through MR analysis, we identified 8 GMs that have a causality with PA, as well as 13 GMs metabolic pathways that have a causality with PA (**Tables 1**, **2**, **Figure 2**, **Supplementary Figure S1**). **Figure 3** shows the causality of the GMs on PA, and we found 1 class, 1 order, 4 genera, and 2 Species of bacteria that are significantly related to the onset of PA. Among them, β-proteobacteria class, Burkholderiales order, and the unclassified genus of Sutterellaceae are positively correlated with the onset of PA, suggesting that it may be an inducible factor for the onset of PA; the unnamed genus of Lachnospiraceae, Rothia genus, the unnamed genus of Erysipelotrichaceae, Faecalibacterium, and Finegoldia are negatively correlated with the onset of PA and can be used as protective factors to prevent the onset of PA.

**Table 1:** The results of the MR analysis between GM and PA (P<1×10-5).

Classification		Nsnp	IVW		Pleiotropy		Heterogeneity		Steiger test
			P	OR (95%Cl)	Egger intercept	P	Q	P	
Class	β-proteobacteria ebi−a−GCST90027657	13	0.0018	1.63 (1.20-2.22)	-0.105	0.155	14.4	0.273	True
Order	Burkholderiales ebi−a−GCST90027743	14	0.0007	1.68 (1.24-2.27)	-0.083	0.198	10.7	0.629	True
Genus	Sutterellaceae_ Unclassified ebi−a−GCST90027728	6	0.015	1.44 (1.07-1.93)	-0.066	0.515	5.2	0.385	True
	Lachnospiraceae_noname ebi−a−GCST90027712	6	0.020	0.58 (0.36-0.92)	0.212	0.109	6.6	0.250	True
	Roseburia ebi−a−GCST90027713	15	0.030	0.75 (0.57-0.97)	-0.006	0.922	17.3	0.240	True
	Erysipelotrichaceae_noname ebi−a- GCST90027720	13	0.027	0.81 (0.67-0.98)	-0.024	0.744	3.1	0.994	True
Species	Faecalibacterium ebi−a−GCST90027826	14	0.037	0.88 (0.77-0.99)	0.003	0.948	3.7	0.993	True
	Finegoldia ebi−a−GCST90027775	9	0.039	0.69 (0.49-0.98)	-0.0007	0.994	3.7	0.883	True

**Table 2:** The results of MR between GM metabolic pathways and PA (P<1×10^-5^).

Pathway	Nsnp	IVW		Pleiotropy		Heterogeneity		Steiger test
		P	OR (95%Cl)	Egger intercept	P	Q	P	
D-galactose degradation V (Leloir pathway) ebi-a-GCST90027592	11	0.0005	0.57 (0.42-0.79)	0.022	0.761	7.1	0.711	True
phosphopantothenate biosynthesis I ebi-a-GCST90027504	11	0.007	0.65 (0.48-0.89)	0.033	0.699	10.0	0.436	True
L-histidine degradation I ebi-a-GCST90027481	12	0.009	1.44 (1.07-1.93)	-0.083	0.258	11.8	0.377	True
E-galactose degradation I (Leloir pathway) ebi-a-GCST90027578	13	0.009	0.67 (0.49-0.91)	-0.027	0.700	18.2	0.108	True
superpathway of N-acetylneuraminate degradation ebi-a-GCST90027502	12	0.017	0.67 (0.48-0.93)	0.047	0.432	12.9	0.293	True
superpathway of fucose and rhamnose degradation ebi-a-GCST90027467	8	0.028	1.33 (1.03-1.71)	-0.051	0.607	7.1	0.415	True
chorismate biosynthesis from 3-dehydroquinate ebi-a-GCST90027573	10	0.033	1.47 (1.03-2.10)	0.091	0.340	5.9	0.742	True
ubiquinol-8 biosynthesis (prokaryotic) ebi-a-GCST90027596	12	0.037	1.26 (1.01-1.56)	0.022	0.782	12.4	0.328	True
urea cycle	8	0.043	0.66 (0.44-0.99)	0.092	0.589	6.3	0.499	True
coenzyme A biosynthesis I (prokaryotic) ebi-a-GCST90027534	10	0.047	1.44 (1.00-2.08)	-0.047	0.566	4.5	0.869	True
tRNA processing ebi-a-GCST90027522	11	0.048	1.37 (1.00-1.86)	0.034	0.641	12.4	0.257	True
superpathway of tetrahydrofolate biosynthesis ebi-a-GCST90027588	13	0.048	0.78 (0.61-1.00)	0.007	0.937	15.1	0.234	True
superpathway of hexuronide and hexuronate degradation ebi-a-GCST90027469	11	0.049	1.40 (1.00-1.96)	-0.002	0.973	6.6	0.760	True

We also observed the results related to the microbial metabolic pathways, among which the biological pathways such as D-galactose degradation I/V(Leloir pathway), phosphopantothenate biosynthesis I, superpathway of N-acetylneuraminate degradation, increased urea cycle, superpathway of tetrahydrofolate biosynthesis are all negatively correlated with the onset of PA and are the protective factors for the onset of PA; L-histidine degradation I, superpathway of fucose and rhamnose degradation, chorismate biosynthesis from 3-dehydroquinate, ubiquinol-8 biosynthesis, coenzyme A biosynthesis I, transfer ribonucleic acid(tRNA) processing and superpathway of hexuronide and hexuronate degradation are positively correlated with the onset of PA and can promote the progression of PA disease.

**Figure 4:**
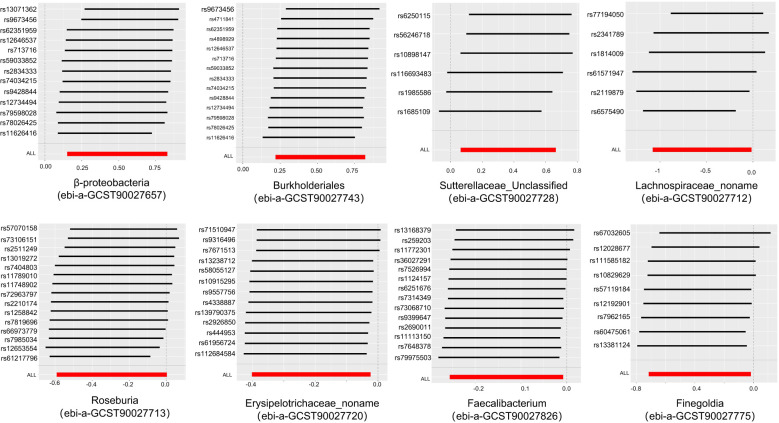
Leave-one-out plots of the causal association between the gut microbiota and pyogenic arthritis.

**Figure 5:**
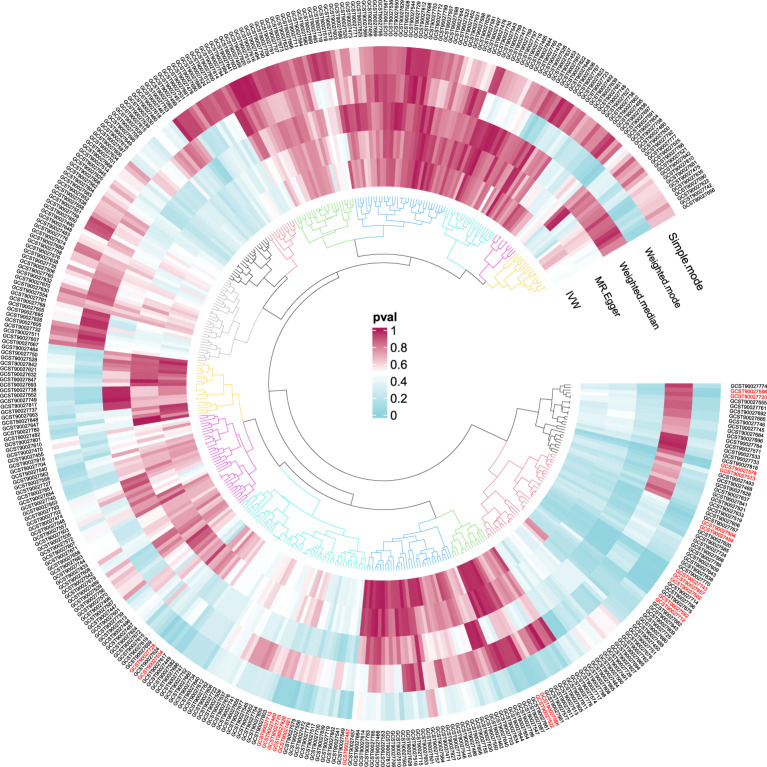
P-value results of MR of GM and pathway on PA.

In order to ensure the validity of IV, we selected the SNPs related to the GM based on the significance threshold of P < 1 × 10^-5^. The low-intensity SNPs with F < 10 were excluded, and the available range of all the SNPs F was from 19.98 to 23.02. In order to ensure the accuracy of the results, we also carried out the analysis of heterogeneity and pleiotropy. The P value of Cochran’s Q test for all the positive results was more than 0.05, indicating that there was no heterogeneity in the results. We also used MR Egger regression to test whether there was multi-level pleiotropy in the results, and likewise, the P value was more than 0.05, indicating that our MR results were not affected by the multi-level pleiotropy. Next, we conducted a leave-one sensitivity analysis. We deleted one SNP each time, recalculated, and checked for outliers that might affect the results. The leave-one sensitivity analysis did not find any abnormal results, further confirming the stability of our research results (**Figure 4**). Finally, we conducted the MR-Steiger test, and the results supported that there was no reverse impact on the significant MR results. **Figure 5** presents the MR research results related to all the GMs and the microbial metabolic pathways and PA, among which the red highlighted parts indicate significant correlation (IVW, P < 0.05), and **Supplementary Table S2** shows all the valid results in the MR.

## 4 Discussion

With the research of MR, we preliminarily investigated the possible causality between GM and PA, and the results indicated that the data sets of 8 GMs and 13 GM metabolic pathways were found to have a significant causality with the risk of PA onset.

The GM, as the “metabolic organ” of the human body, has been the focus of intensive research for many years. Based on its regulatory effect on the whole body, it is closely related to a variety of diseases. It is generally believed that the gut microbial community will produce a variety of compounds, including enzymes and short-chain fatty acids. These metabolites will affect the body’s endocrine system, thereby affecting physical health (Lui, 2024). Based on the interaction between the gut and the joint, this connection is defined as the “gut-joint axis”. These compounds produced by bacteria will be transferred from “leaky gut” to the blood, and then reach the joint to affect the disease process (Longo et al., 2024). Normally, the intestinal epithelium is capable of defending against harmful pathogens. The tight junction proteins can provide protection between intestinal epithelial cells. However, after intestinal dysbiosis, the tight junction proteins are disrupted or inhibited, thereby increasing intestinal permeability and causing inflammatory reactions (Tu et al., 2021). The GM can influence the human immunity system,it play a role in the release of inflammatory factors, and is regarded as a powerful immune modulator (Jiang, 2024). By activating toll-like receptors, they can stimulate the release of pro-inflammatory cytokines. The released cytokines are capable of regulating the inflammatory response and bone remodeling (Kubatzky et al., 2018).

In our research, β-proteobacteria, Burkholderiales, and an unclassified genus of Sutterellaceae were significantly positively correlated with the onset of PA. Sutterellaceae has the ability to inhibit the degradation of Immunoglobulin A(IgA), and they also secrete IgA proteases to degrade IgA, thereby reducing the concentration of IgA in the intestinal mucosa and damaging the function of the intestinal antibacterial immune response (Kaakoush, 2020). Some studies also show that the abundance of Sutterellaceae is negatively correlated with the levels of inflammatory cytokines [Interleukin (IL)-12, IL-13, Interferon gamma (IFN-γ)]. In a set of *in vitro* experiments, Sutterella can adhere to intestinal epithelial cells and promote the secretion of IL-8 (Hiippala et al., 2016). Meanwhile, fucoidan is a kind of complex sulfated polysaccharide derived from brown algae and is regarded as a protein prebiotic. It can alter the gut microbiota, slow down the intestinal mucosal damage induced by cyclophosphamide, and reduce the antigen load and inflammatory response in the host. Furthermore, fucoidan has a significant effect on reducing the abundance of Sutterella (Xue et al., 2018). In our results, this point is also suggested, and the superpathway of fucose and rhamnose degradation is significantly negatively correlated with the PA disease, which confirms the previous research results.

On the contrary, the research results show five other kinds of GMs that have a protective effect on PA. The Lachnospiraceae is recognized as a kind of probiotic in the human body, and some studies have shown that it is related to a variety of human diseases. Cross-sectional studies found that there are fewer Lachnospiraceae and Peptostreptococcaceae in patients with type II diabetes (Maskarinec et al., 2021). Research on the GM and systemic lupus erythematosus (SLE) shows that Lachnospira is negatively correlated with the risk of SLE (Xiang et al., 2021), its protective effect in PA patients was discovered for the first time in this study, and it may be achieved through the fermentation of pectin, polygalacturonic acid, fructose, and cellobiose, etc., among which acetate, formate, ethanol, and CO_2_ are the main final products of the fermentation of polygalacturonic acid and pectin (Carasso et al., 2021). Interestingly, this also coincides with the research results of our study on bacterial metabolic pathways, for example: D-galactose degradation I/V (Leloir pathway) is negatively correlated with the disease progression, providing a protective effect.The genus Rothia in the Lachnospiraceae can produce short-chain fatty acids, especially butyric acid, which affects colon movement and has anti-inflammatory properties (Van den Abbeele et al., 2013). Some studies (Wu et al., 2022) have focused on butyrate and showed that it can reduce macrophage apoptosis and inhibit osteoclast generation, demonstrating its protective effect in the development of PA.

Bacteroides is one of the important bacteria in the human colon and is capable of participating in the decomposition of carbohydrates, the utilization of nitrogen-containing substances, and the biotransformation process of steroid substances (Gibiino et al., 2018). In different researches, the role played by Bacteroides is also different, such as the increase in the content of Bacteroides in colorectal cancer, which promotes the progress of the disease (Wang et al., 2023). Recently discovered high urea load can promote the differentiation of macrophages towards the immunosuppressive subtype, thereby disrupting the intestinal immune homeostasis, and this mechanism is achieved through the gut microbiota, especially Bacteroides (Chen et al., 2023). On the contrary, when studying metabolic diseases such as obesity (Ley et al., 2006), the relative proportion of Bacteroidetes in obese people has decreased, while after using two kinds of low-calorie diets to lose weight, this proportion has increased. Such different results may be related to the different roles played by different species within Bacteroidetes. The conclusion drawn from our study is that Bacteroidetes, especially Bacteroides coprocola and Finegoldia magna, have a protective effect in the onset of PA, and they may play a role through bacterial pathways such as the urea cycle.

On the other hand, there are still some microbiota within the bone and joint. Different microbial communities can serve as a defense system against the invasion of pathogens. However, this kind of microbiota imbalance will benefit the harmful bacteria to the human body and eventually lead to bone and joint infections (Chen et al., 2021). In addition, these microbiota can simultaneously regulate the immune response of the bone and joint. The imbalance of the microbiota will lead to a decrease in immune defense, making the bone and joint more vulnerable to infection (Gilat et al., 2024). In some cases, bacteria can form stubborn biofilms within the joint, which is one of the causes of chronic infections. Besides, the interaction between microbiota in various parts can affect joint health by regulating the immune process of the body. In 2020, Christopher M. Dunn and others’ laboratory published information on 42 complete human cartilage microbial DNA samples for the first time. When comparing diseased human tissues with disease-free controls, significant changes occurred in the cartilage microbial DNA patterns, including the loss of alpha diversity and the enrichment of Gram-negative components. And the inferred metagenomic differences in cartilage samples are consistent with gut microbiome studies, including the enrichment of phosphatidylinositol signaling pathways and the increase in lipopolysaccharide pathways, suggesting that the joint microbiome may be related to the gut microbiome (Dunn et al., 2020). Current research typically focuses on identifying specific microbes in the joint and understanding how these microbes interact with the host’s immune system.The specific mechanism of the interaction between the joint microbiota and PA in the future is an aspect worthy of exploration.

Our research’s advantage mainly lies in that we have used the method of double-sample MR to comprehensively analyze the relationship between the GM and related metabolic pathways and PA. Firstly, according to Mendelian genetics law, alleles are randomly assigned to offspring, similar to the randomization in RCT. In addition, the genotype is fixed at the time of conception and cannot be changed by the disease (Zheng et al., 2017). Therefore, causal inference is less likely to be affected by reverse causality and confounding factors. Secondly, the data used in the MR analysis all come from publicly available databases and do not require additional expenses. These MR findings have important significance for public health, and by introducing the perspective of genetics, they enrich the previous research on the association between the GM and PA. From the perspective of preventive medicine, these results can provide information for PA prevention strategies by timely regulating the GM. In terms of diagnosis, it can remind people of conducting the screening and early diagnosis of PA through GM. Through the analysis of the microbial community metabolic pathways, the potentially related bacterial metabolic pathways with PA have been preliminarily understood, providing a new direction for the study of its mechanism. However, our study still has several limitations. Firstly, when processing GM data, when the sensitivity threshold for setting is p < 5 × 10^−8^, there are too few SNPs, Therefore, we chose p < 1 × 10^−5^ to select SNPs of the GM. We screened weak IVs through F, excluded SNPs with F < 10, and manually removed SNPs that might have confounding factors to ensure the reliability of IVs. Secondly, there are still many other microbial groups whose potential causal relationship with PA has not been explored. In addition, most of the samples in this study are from Europe, which imposes limitations on the research results.

## 5 Conclusion

In conclusion, our research has for the first time demonstrated the influence of the GM on PA, and also to a certain extent, improved the content of the “intestine-joint” axis. Through our research, it is indicated that the bacteria of β-proteobacteria may become a marker of PA, and they may promote the progress of PA through the superpathway of fucose and rhamnose degradation. These findings can provide new ideas for the prevention and treatment of PA. When there is a clinical suspicion of PA, the identification of the fecal microbiota can be used to see if there is an increase in β-proteobacteria to achieve the purpose of early diagnosis and early treatment. And based on our results, it is possible to develop drug preparations that are in conflict with β-proteobacteria, or new probiotics, such as Lachnospira, for the treatment of PA. In the future, the inherent mechanism of action between the GM and PA may be clarified through research. Only in this way can they be able to offer effective treatment and diagnosis schemes for PA patients, benefit the vast number of patients afflicted with this disease and reduce the burden on families.

## Data Availability

Publicly available datasets were analyzed for this study. These data can be found here: Gut microbiota GWAS data from MiBioGen (https://mibiogen.gcc.rug.nl/) and EBI database (http://ftp.ebi.ac.uk/pub/databases/gwas/summary_statistics/GCST90027001-GCST90028000), Pyogenic arthritis GWAS data from FinnGen R10 project database (https://r10.finngen.fi/pheno/M13_PYOGARTH).

## Author's note

No financial or other relationships that could potentially influence the objectivity and fairness of the research and its presentation have been established. The research and its findings are based solely on academic pursuit and the pursuit of truth, without any external biases or influences that might compromise the integrity of the work. All data and analyses presented in this paper are the result of independent and objective investigations. This statement is made to ensure the transparency and credibility of the research and to affirm the commitment of the authors to ethical and objective scholarly communication.
