# Supplementary material for: The causal relationship between the human gut microbiota and pyogenic arthritis: a Mendelian randomization study
Source: Front Cell Infect Microbiol. 2024 Nov 26;14:1452480. doi: 10.3389/fcimb.2024.1452480 (PMC11629706; doi:10.3389/fcimb.2024.1452480)
Supplement: Supplementary file 1 [file DataSheet1.zip › mendelian test/ebi-a-GCST90027449.csv_forest.pdf]

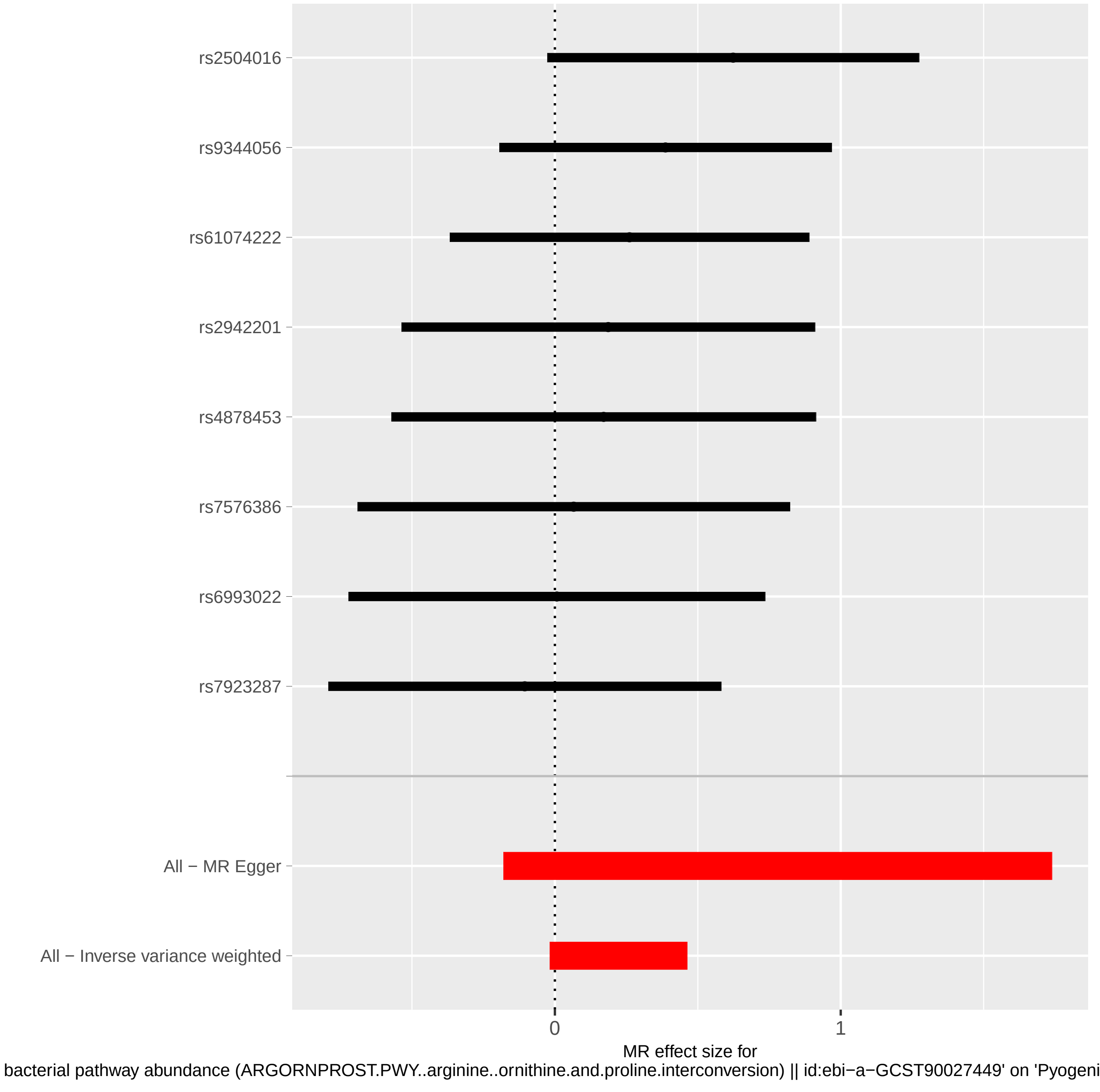

rs2504016
rs9344056
rs61074222
rs2942201
rs4878453
rs7576386
rs6993022
rs7923287
All − MR Egger
All − Inverse variance weighted
0
1
MR effect size for
bacterial pathway abundance (ARGORNPROST.PWY..arginine..ornithine.and.proline.interconversion) || id:ebi−a−GCST90027449' on 'Pyogeni
