# Supplementary material for: The causal relationship between the human gut microbiota and pyogenic arthritis: a Mendelian randomization study
Source: Front Cell Infect Microbiol. 2024 Nov 26;14:1452480. doi: 10.3389/fcimb.2024.1452480 (PMC11629706; doi:10.3389/fcimb.2024.1452480)
Supplement: Supplementary file 1 [file DataSheet1.zip › mendelian test/ebi-a-GCST90027450.csv_forest.pdf]

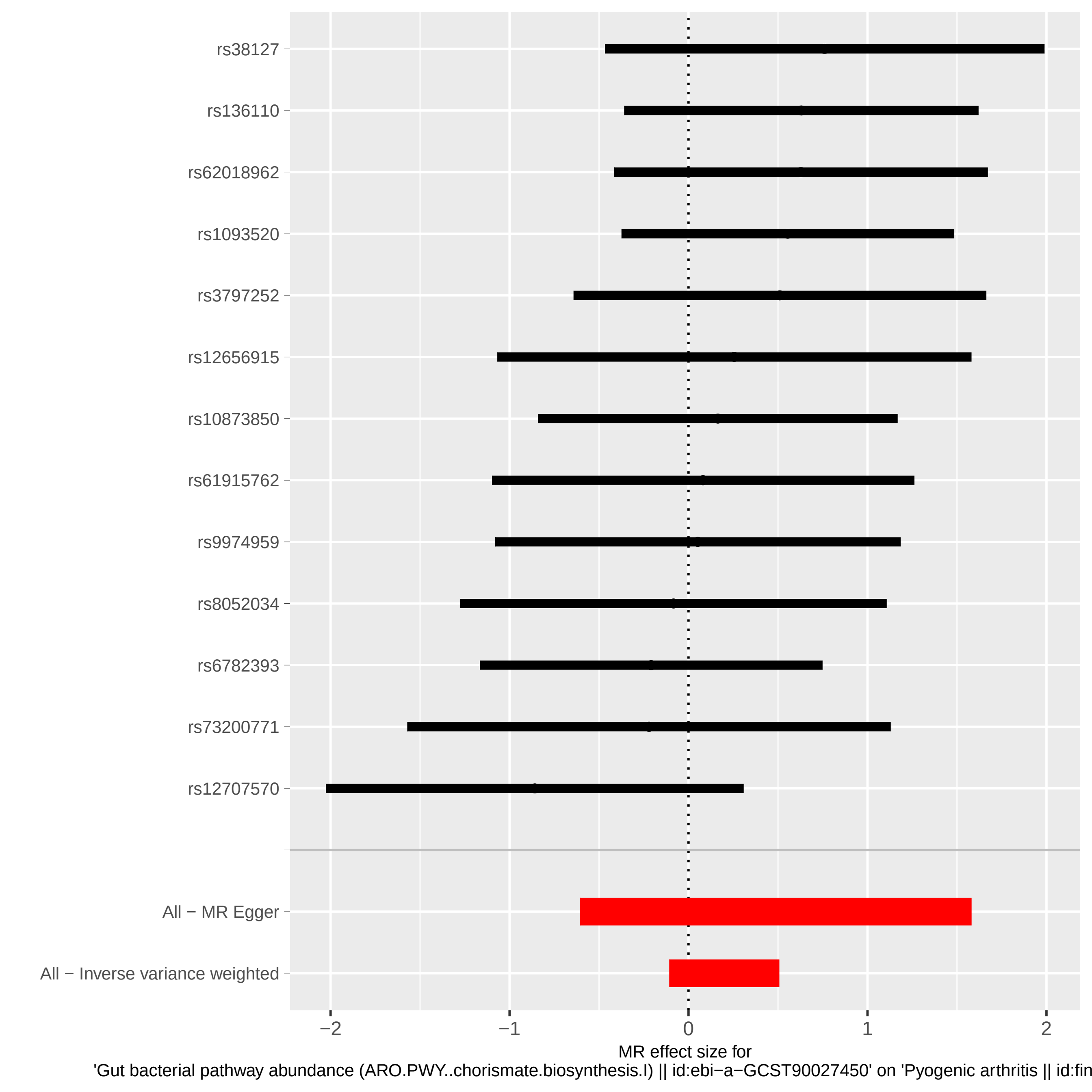
rs38127
rs136110
rs62018962
rs1093520
rs3797252
rs12656915
rs10873850
rs61915762
rs9974959
rs8052034
rs6782393
rs73200771
rs12707570
All − MR Egger
All − Inverse variance weighted
−2
−1
0
1
2
MR effect size for
'Gut bacterial pathway abundance (ARO.PWY..chorismate.biosynthesis.I) || id:ebi−a−GCST90027450' on 'Pyogenic arthritis || id:fin
