# Supplementary material for: The causal relationship between the human gut microbiota and pyogenic arthritis: a Mendelian randomization study
Source: Front Cell Infect Microbiol. 2024 Nov 26;14:1452480. doi: 10.3389/fcimb.2024.1452480 (PMC11629706; doi:10.3389/fcimb.2024.1452480)
Supplement: Supplementary file 1 [file DataSheet1.zip › mendelian test/ebi-a-GCST90027453.csv_forest.pdf]

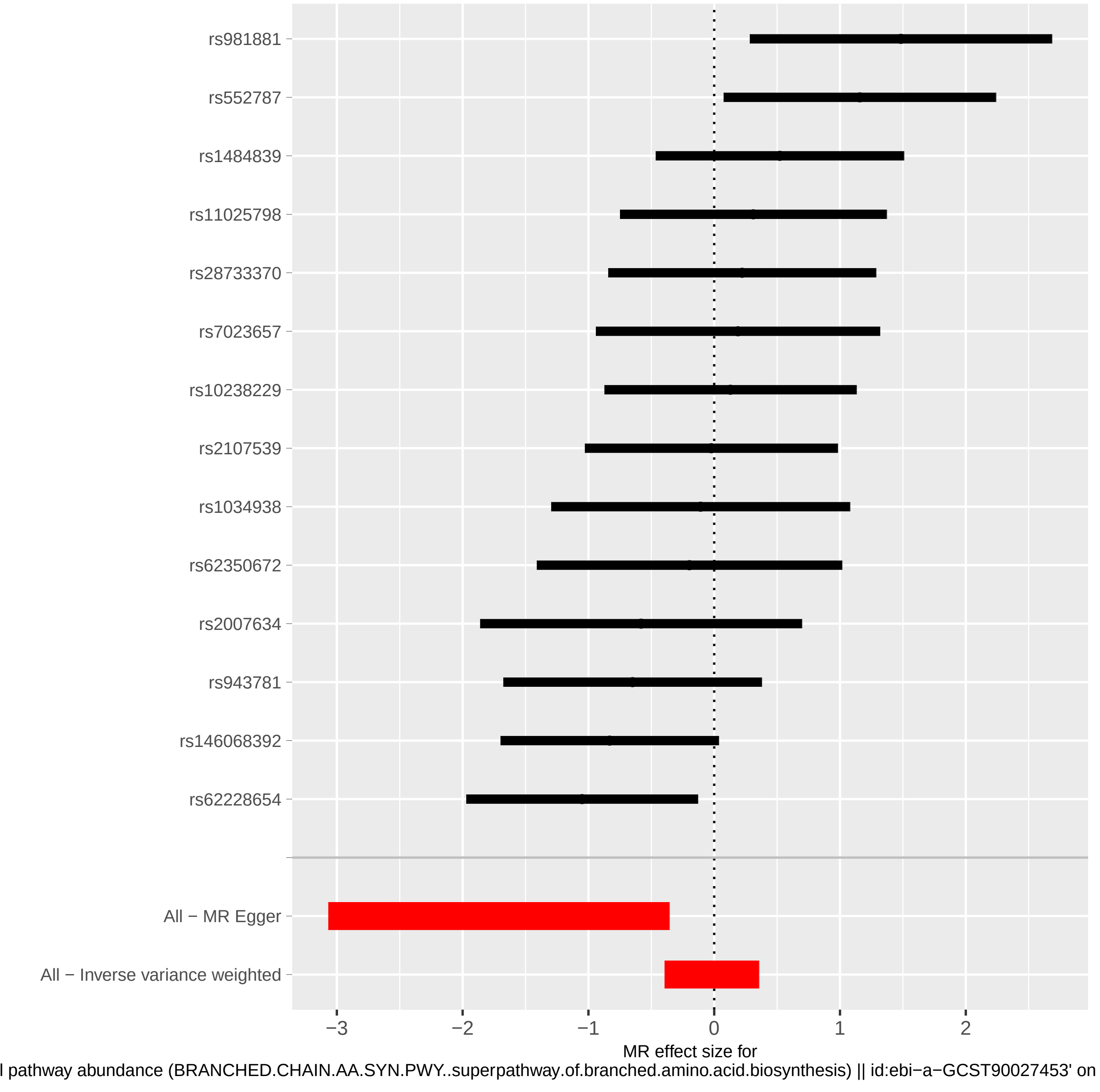
rs981881
rs552787
rs1484839
rs11025798
rs28733370
rs7023657
rs10238229
rs2107539
rs1034938
rs62350672
rs2007634
rs943781
rs146068392
rs62228654
All − MR Egger
All − Inverse variance weighted
−3
−2
−1
0
1
2
MR effect size for
l pathway abundance (BRANCHED.CHAIN.AA.SYN.PWY..superpathway.of.branched.amino.acid.biosynthesis) || id:ebi−a−GCST90027453' on
