# Supplementary material for: The causal relationship between the human gut microbiota and pyogenic arthritis: a Mendelian randomization study
Source: Front Cell Infect Microbiol. 2024 Nov 26;14:1452480. doi: 10.3389/fcimb.2024.1452480 (PMC11629706; doi:10.3389/fcimb.2024.1452480)
Supplement: Supplementary file 1 [file DataSheet1.zip › mendelian test/ebi-a-GCST90027455.csv_forest.pdf]

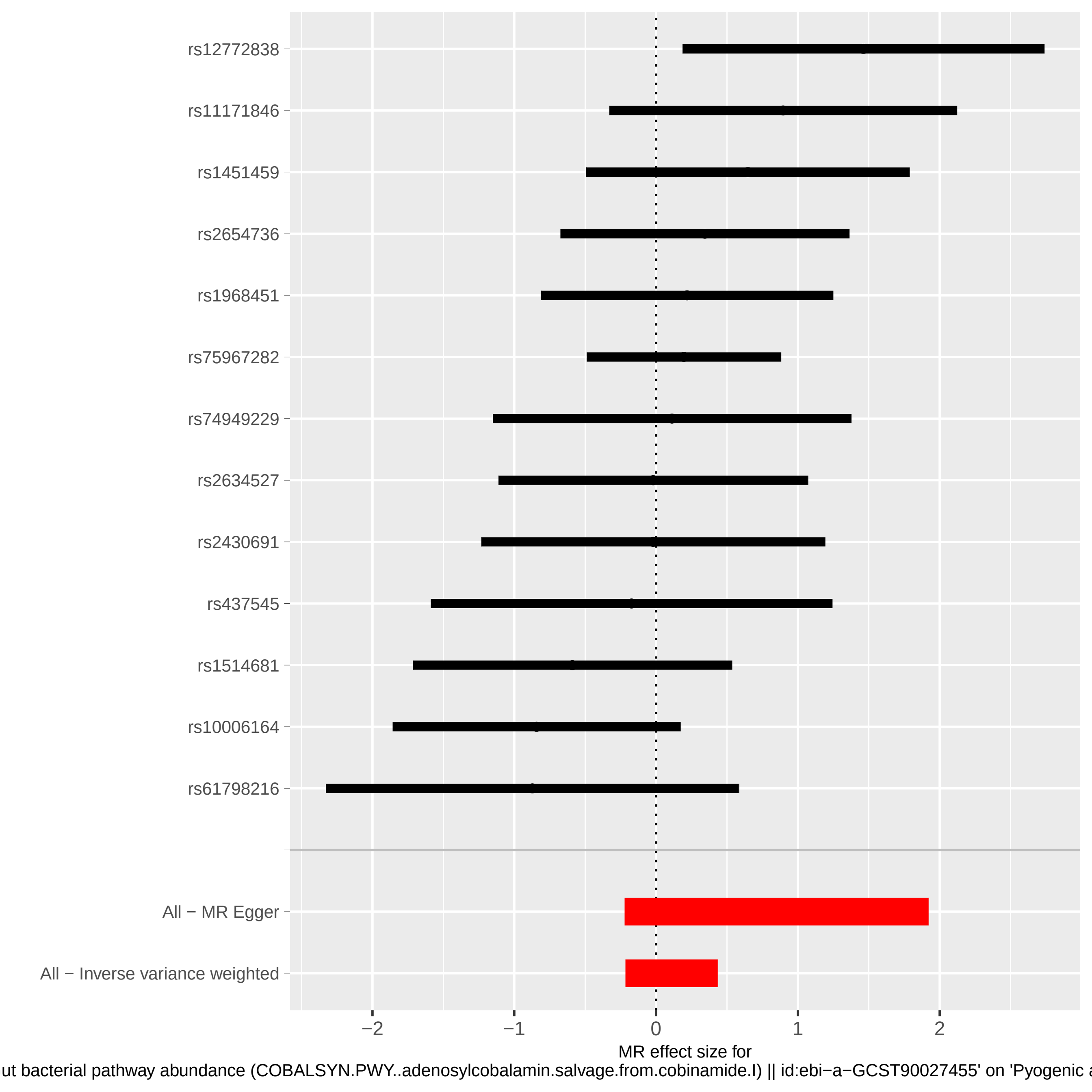

rs12772838
rs11171846
rs1451459
rs2654736
rs1968451
rs75967282
rs74949229
rs2634527
rs2430691
rs437545
rs1514681
rs10006164
rs61798216
All − MR Egger
All − Inverse variance weighted
−2
−1
0
1
2
MR effect size for
ut bacterial pathway abundance (COBALSYN.PWY..adenosylcobalamin.salvage.from.cobinamide.I) || id:ebi−a−GCST90027455' on 'Pyogenic a
