# Supplementary material for: The causal relationship between the human gut microbiota and pyogenic arthritis: a Mendelian randomization study
Source: Front Cell Infect Microbiol. 2024 Nov 26;14:1452480. doi: 10.3389/fcimb.2024.1452480 (PMC11629706; doi:10.3389/fcimb.2024.1452480)
Supplement: Supplementary file 1 [file DataSheet1.zip › mendelian test/ebi-a-GCST90027457.csv_forest.pdf]

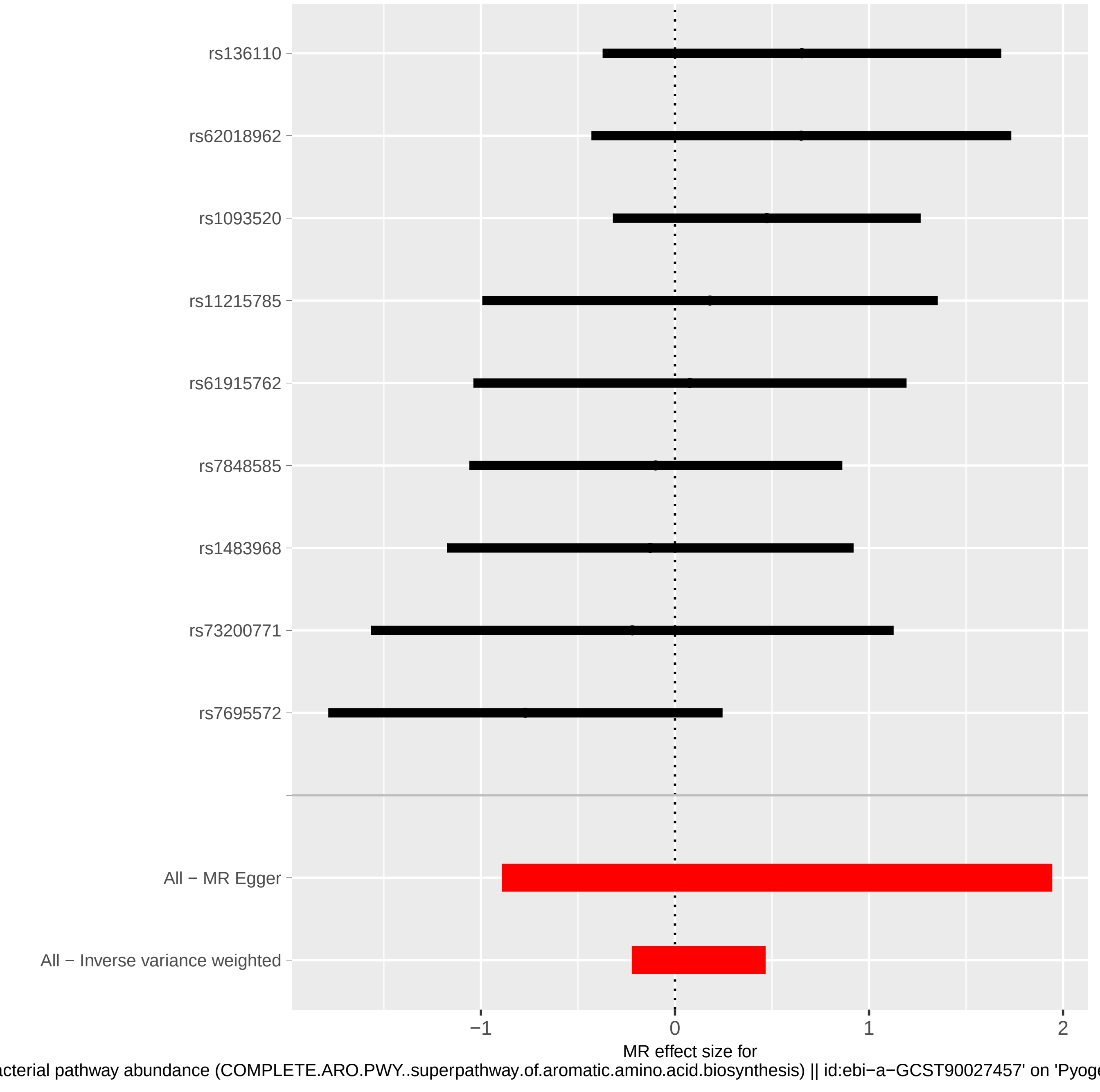

rs136110
rs62018962
rs1093520
rs11215785
rs61915762
rs7848585
rs1483968
rs73200771
rs7695572
All − MR Egger
All − Inverse variance weighted
−1
0
1
2
MR effect size for
acterial pathway abundance (COMPLETE.ARO.PWY..superpathway.of.aromatic.amino.acid.biosynthesis) || id:ebi−a−GCST90027457' on 'Pyoge
