# Supplementary material for: The causal relationship between the human gut microbiota and pyogenic arthritis: a Mendelian randomization study
Source: Front Cell Infect Microbiol. 2024 Nov 26;14:1452480. doi: 10.3389/fcimb.2024.1452480 (PMC11629706; doi:10.3389/fcimb.2024.1452480)
Supplement: Supplementary file 1 [file DataSheet1.zip › mendelian test/ebi-a-GCST90027459.csv_forest.pdf]

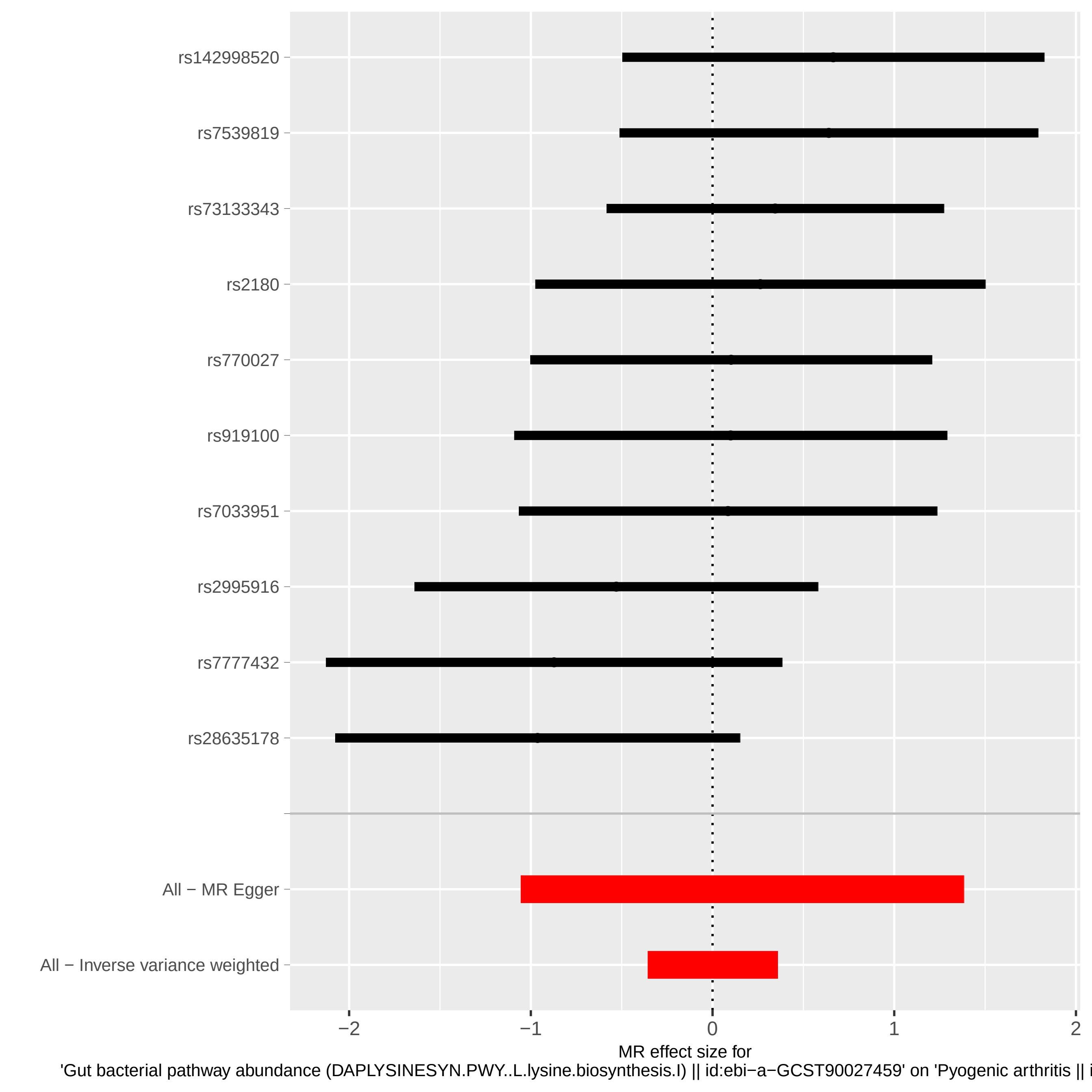
rs142998520
rs7539819
rs73133343
rs2180
rs770027
rs919100
rs7033951
rs2995916
rs7777432
rs28635178
All − MR Egger
All − Inverse variance weighted
−2
−1
0
1
2
MR effect size for
'Gut bacterial pathway abundance (DAPLYSINESYN.PWY..L.lysine.biosynthesis.I) || id:ebi−a−GCST90027459' on 'Pyogenic arthritis || i
