# Supplementary material for: The causal relationship between the human gut microbiota and pyogenic arthritis: a Mendelian randomization study
Source: Front Cell Infect Microbiol. 2024 Nov 26;14:1452480. doi: 10.3389/fcimb.2024.1452480 (PMC11629706; doi:10.3389/fcimb.2024.1452480)
Supplement: Supplementary file 1 [file DataSheet1.zip › mendelian test/ebi-a-GCST90027459.csv_leave_one_out.pdf]

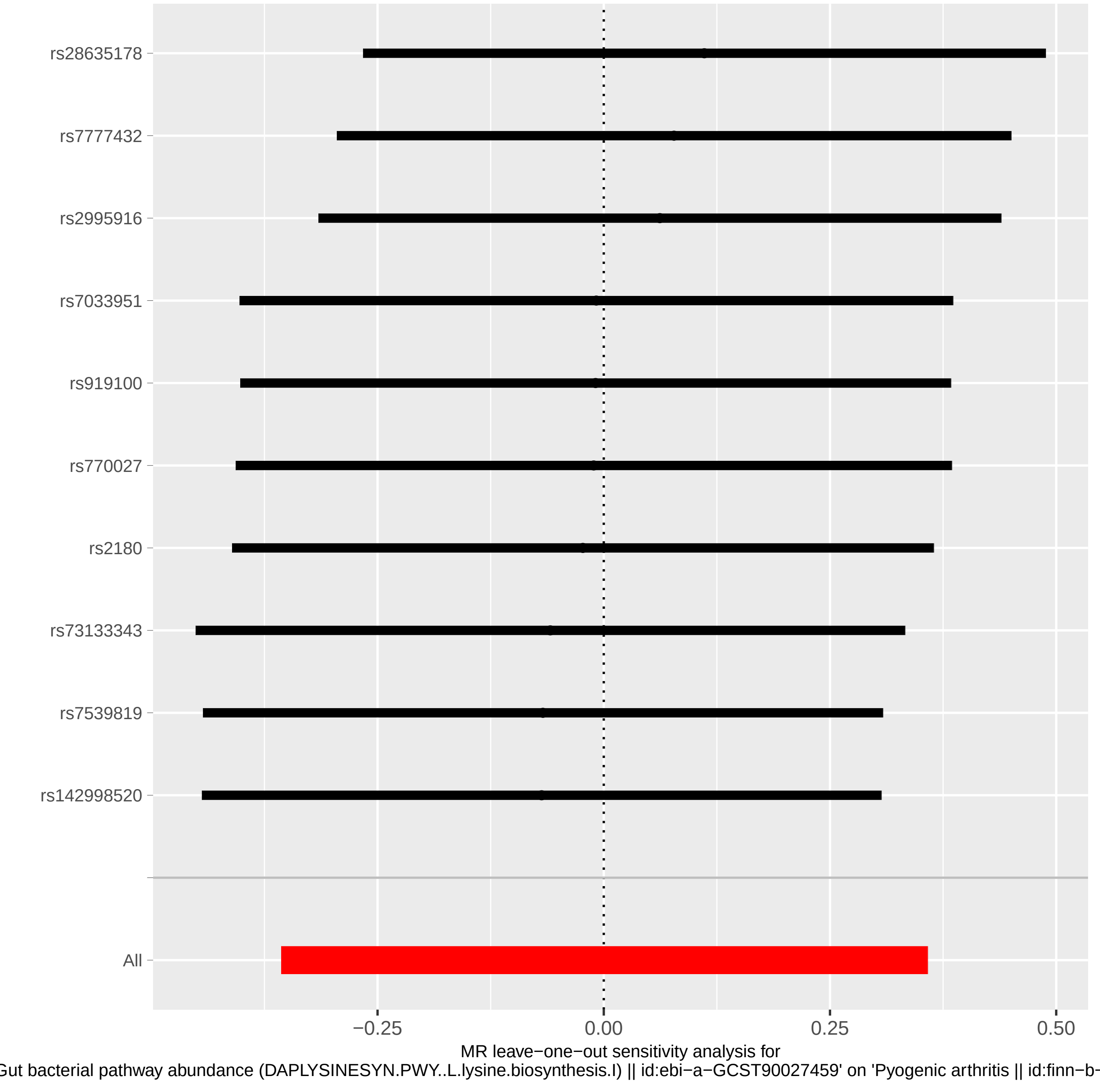

rs28635178
rs7777432
rs2995916
rs7033951
rs919100
rs770027
rs2180
rs73133343
rs7539819
rs142998520
All
−0.25
0.00
0.25
0.50
MR leave−one−out sensitivity analysis for
Gut bacterial pathway abundance (DAPLYSINESYN.PWY..L.lysine.biosynthesis.I) || id:ebi−a−GCST90027459' on 'Pyogenic arthritis || id:finn−b−
