# Supplementary material for: The causal relationship between the human gut microbiota and pyogenic arthritis: a Mendelian randomization study
Source: Front Cell Infect Microbiol. 2024 Nov 26;14:1452480. doi: 10.3389/fcimb.2024.1452480 (PMC11629706; doi:10.3389/fcimb.2024.1452480)
Supplement: Supplementary file 1 [file DataSheet1.zip › mendelian test/ebi-a-GCST90027460.csv_forest.pdf]

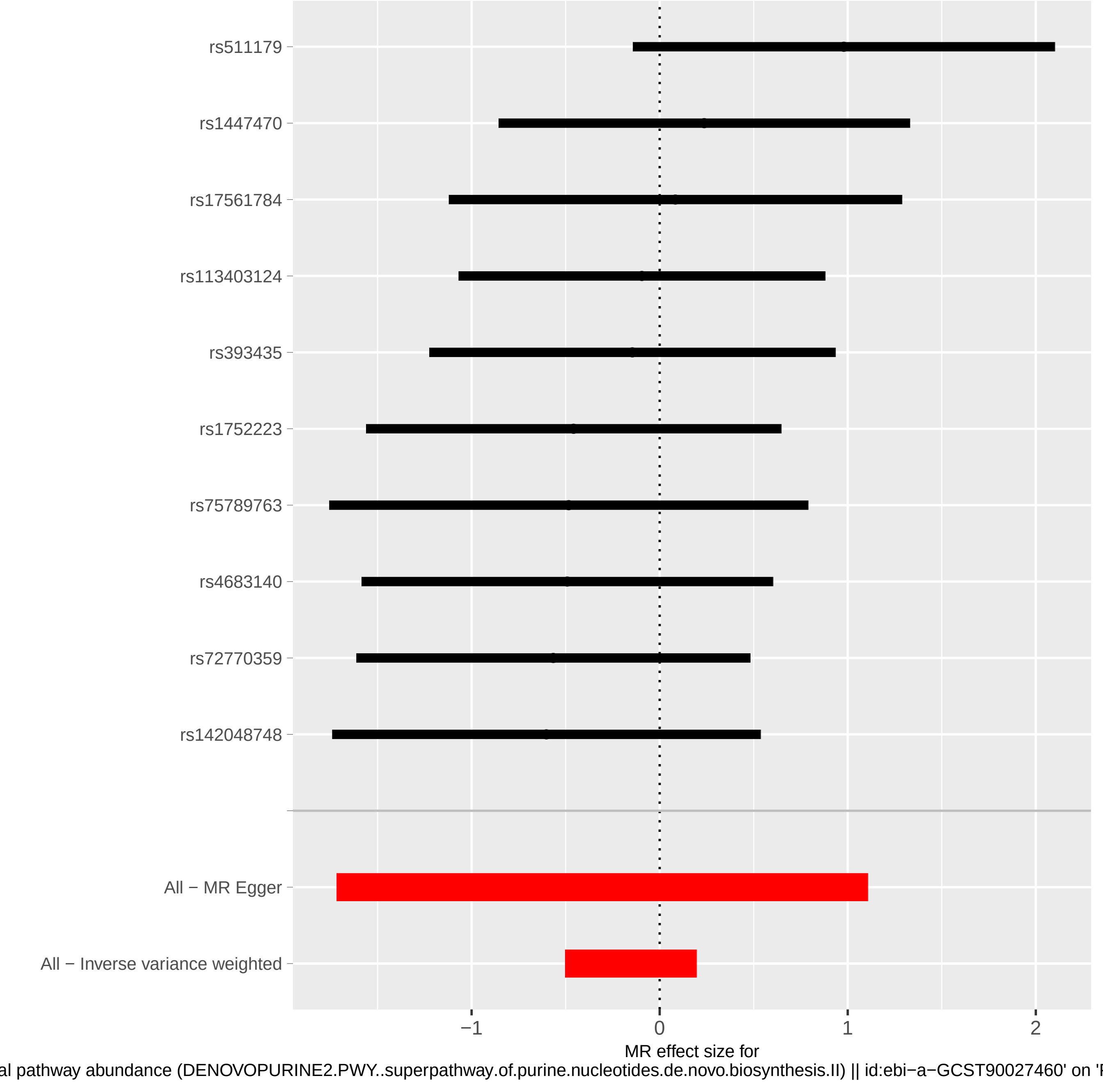
rs511179
rs1447470
rs17561784
rs113403124
rs393435
rs1752223
rs75789763
rs4683140
rs72770359
rs142048748
All − MR Egger
All − Inverse variance weighted
−1
0
1
2
MR effect size for
al pathway abundance (DENOVOPURINE2.PWY..superpathway.of.purine.nucleotides.de.novo.biosynthesis.II) || id:ebi−a−GCST90027460' on 'F
