# Supplementary material for: The causal relationship between the human gut microbiota and pyogenic arthritis: a Mendelian randomization study
Source: Front Cell Infect Microbiol. 2024 Nov 26;14:1452480. doi: 10.3389/fcimb.2024.1452480 (PMC11629706; doi:10.3389/fcimb.2024.1452480)
Supplement: Supplementary file 1 [file DataSheet1.zip › mendelian test/ebi-a-GCST90027464.csv_forest.pdf]

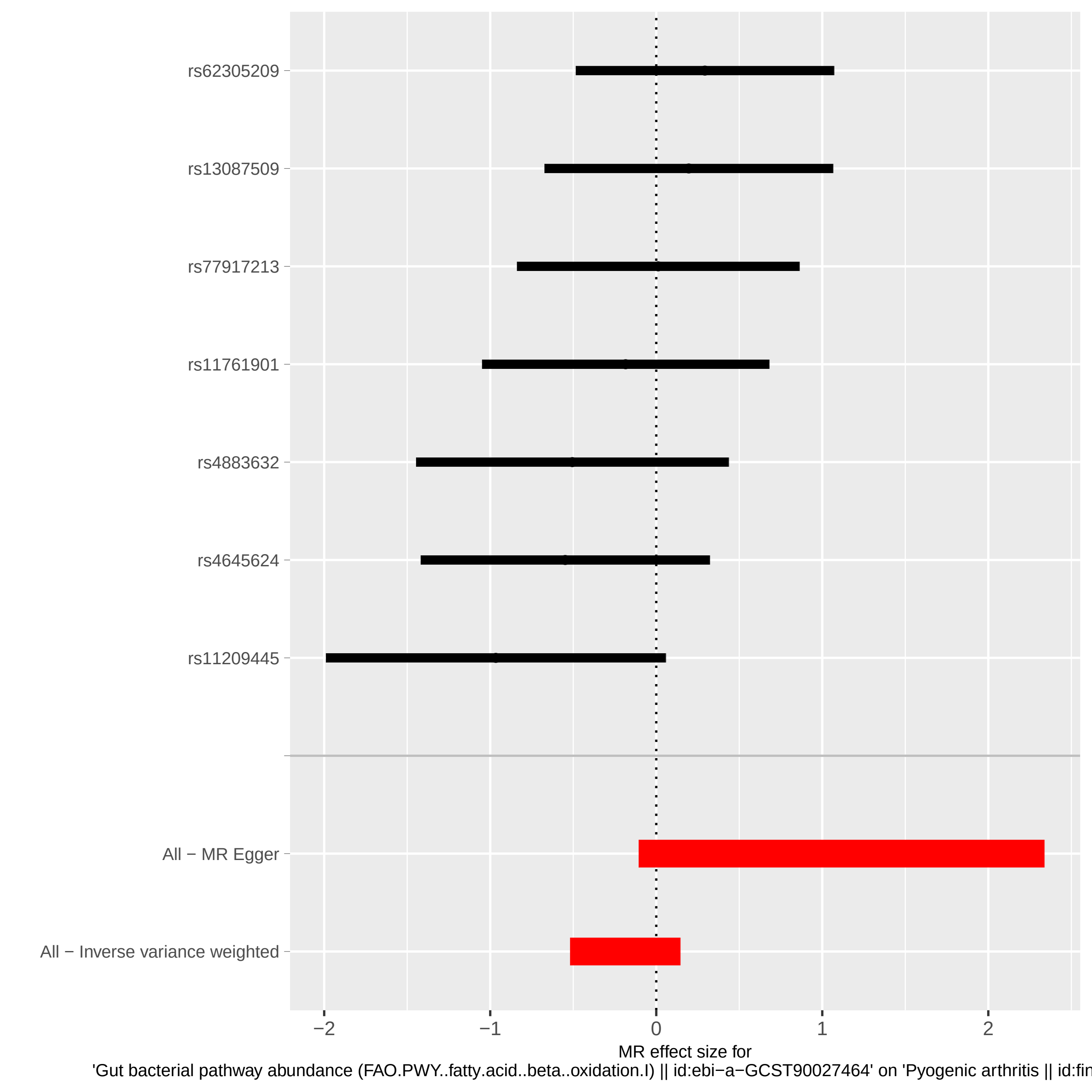

rs62305209
rs13087509
rs77917213
rs11761901
rs4883632
rs4645624
rs11209445
All − MR Egger
All − Inverse variance weighted
−2
−1
0
1
2
MR effect size for
'Gut bacterial pathway abundance (FAO.PWY..fatty.acid..beta..oxidation.I) || id:ebi−a−GCST90027464' on 'Pyogenic arthritis || id:fin
