# Supplementary material for: The causal relationship between the human gut microbiota and pyogenic arthritis: a Mendelian randomization study
Source: Front Cell Infect Microbiol. 2024 Nov 26;14:1452480. doi: 10.3389/fcimb.2024.1452480 (PMC11629706; doi:10.3389/fcimb.2024.1452480)
Supplement: Supplementary file 1 [file DataSheet1.zip › mendelian test/ebi-a-GCST90027468.csv_forest.pdf]

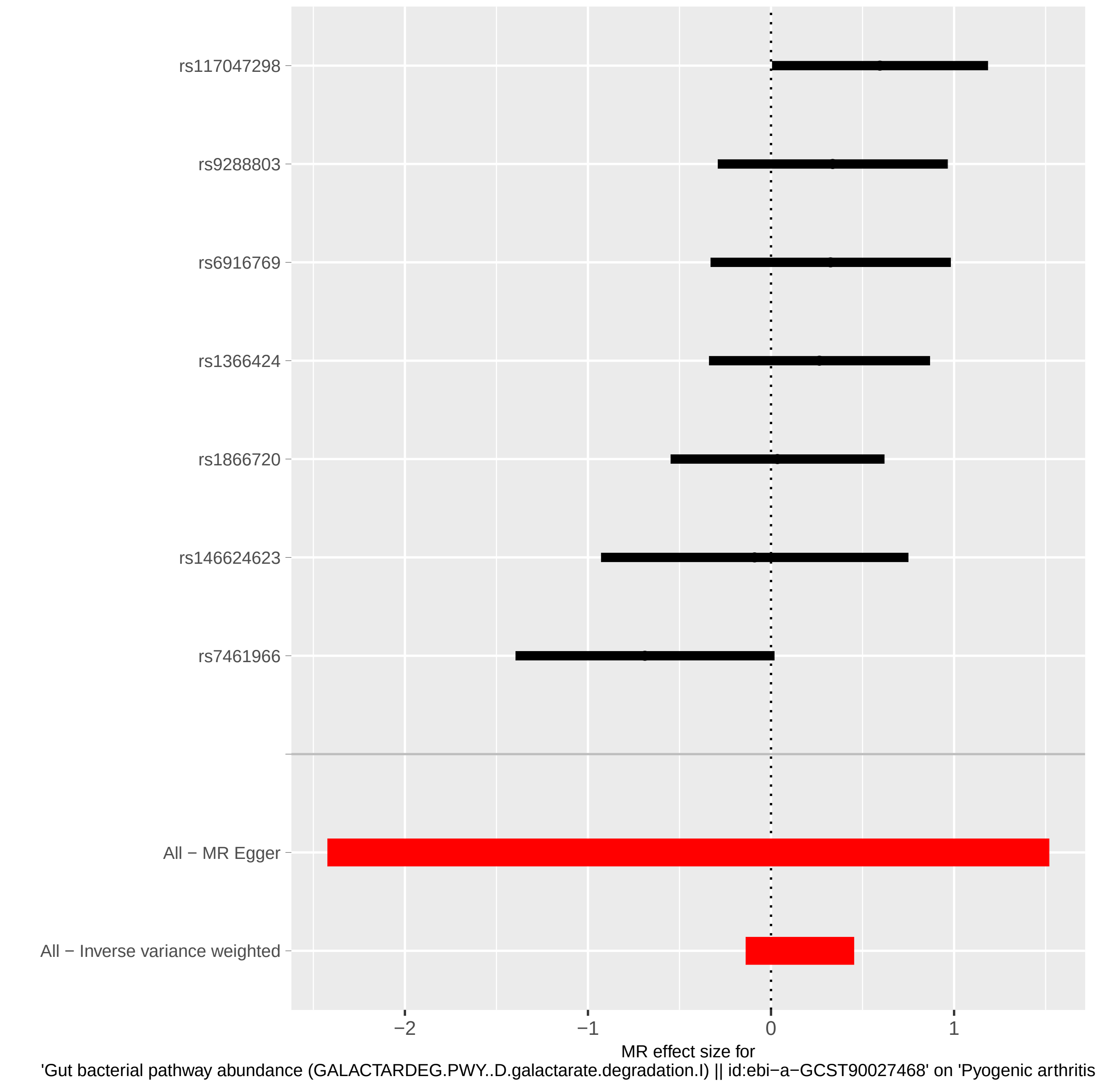
rs117047298
rs9288803
rs6916769
rs1366424
rs1866720
rs146624623
rs7461966
All − MR Egger
All − Inverse variance weighted
−2
−1
0
1
MR effect size for
'Gut bacterial pathway abundance (GALACTARDEG.PWY..D.galactarate.degradation.I) || id:ebi−a−GCST90027468' on 'Pyogenic arthritis
