# Supplementary material for: The causal relationship between the human gut microbiota and pyogenic arthritis: a Mendelian randomization study
Source: Front Cell Infect Microbiol. 2024 Nov 26;14:1452480. doi: 10.3389/fcimb.2024.1452480 (PMC11629706; doi:10.3389/fcimb.2024.1452480)
Supplement: Supplementary file 1 [file DataSheet1.zip › mendelian test/ebi-a-GCST90027471.csv_forest.pdf]

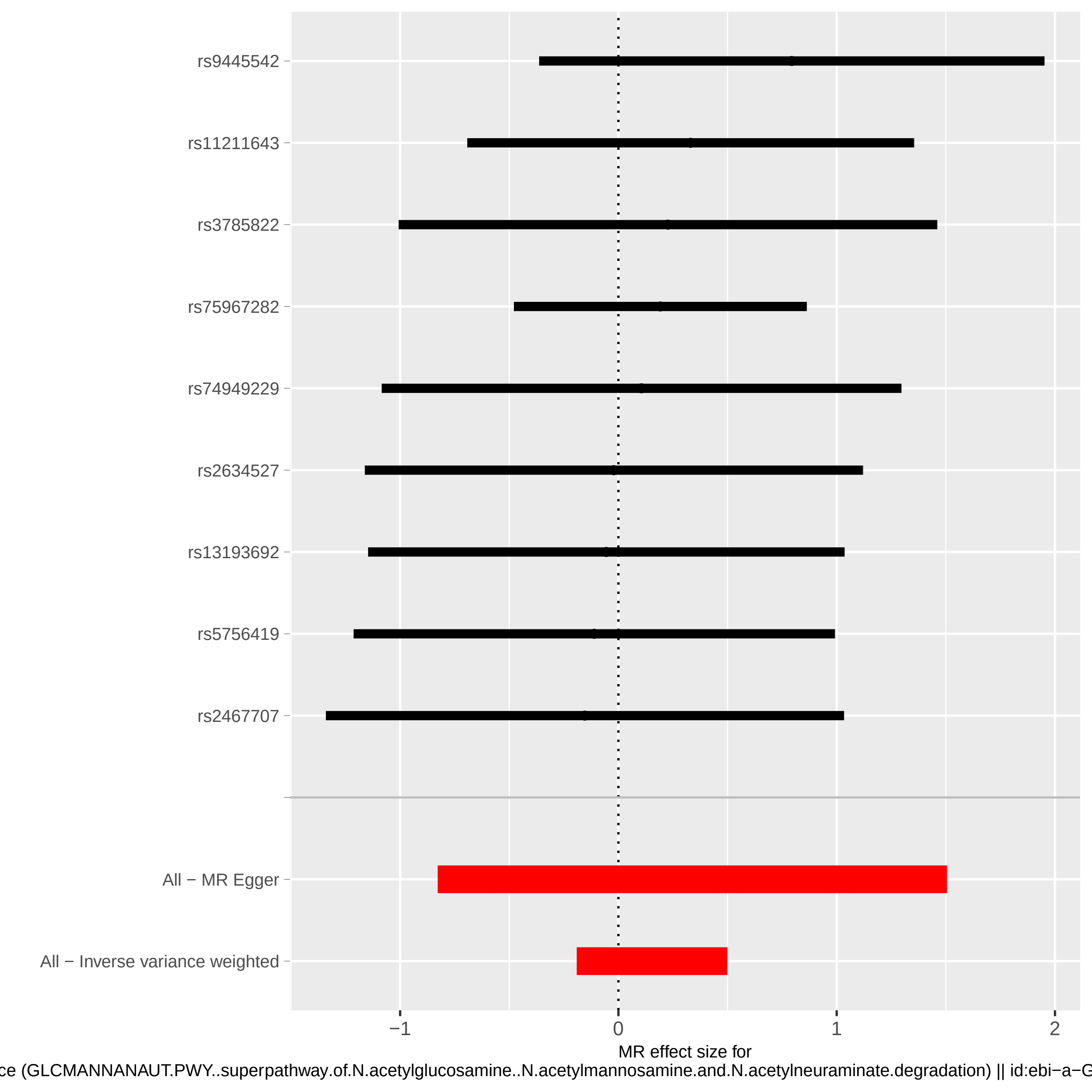

rs9445542
rs11211643
rs3785822
rs75967282
rs74949229
rs2634527
rs13193692
rs5756419
rs2467707
All − MR Egger
All − Inverse variance weighted
−1
0
1
2
MR effect size for
ce (GLCMANNANAUT.PWY..superpathway.of.N.acetylglucosamine..N.acetylmannosamine.and.N.acetylneuraminate.degradation) || id:ebi−a−G
