# Supplementary figures and images for: The causal relationship between the human gut microbiota and pyogenic arthritis: a Mendelian randomization study
Source: Front Cell Infect Microbiol. 2024 Nov 26;14:1452480. doi: 10.3389/fcimb.2024.1452480 (PMC11629706; doi:10.3389/fcimb.2024.1452480)

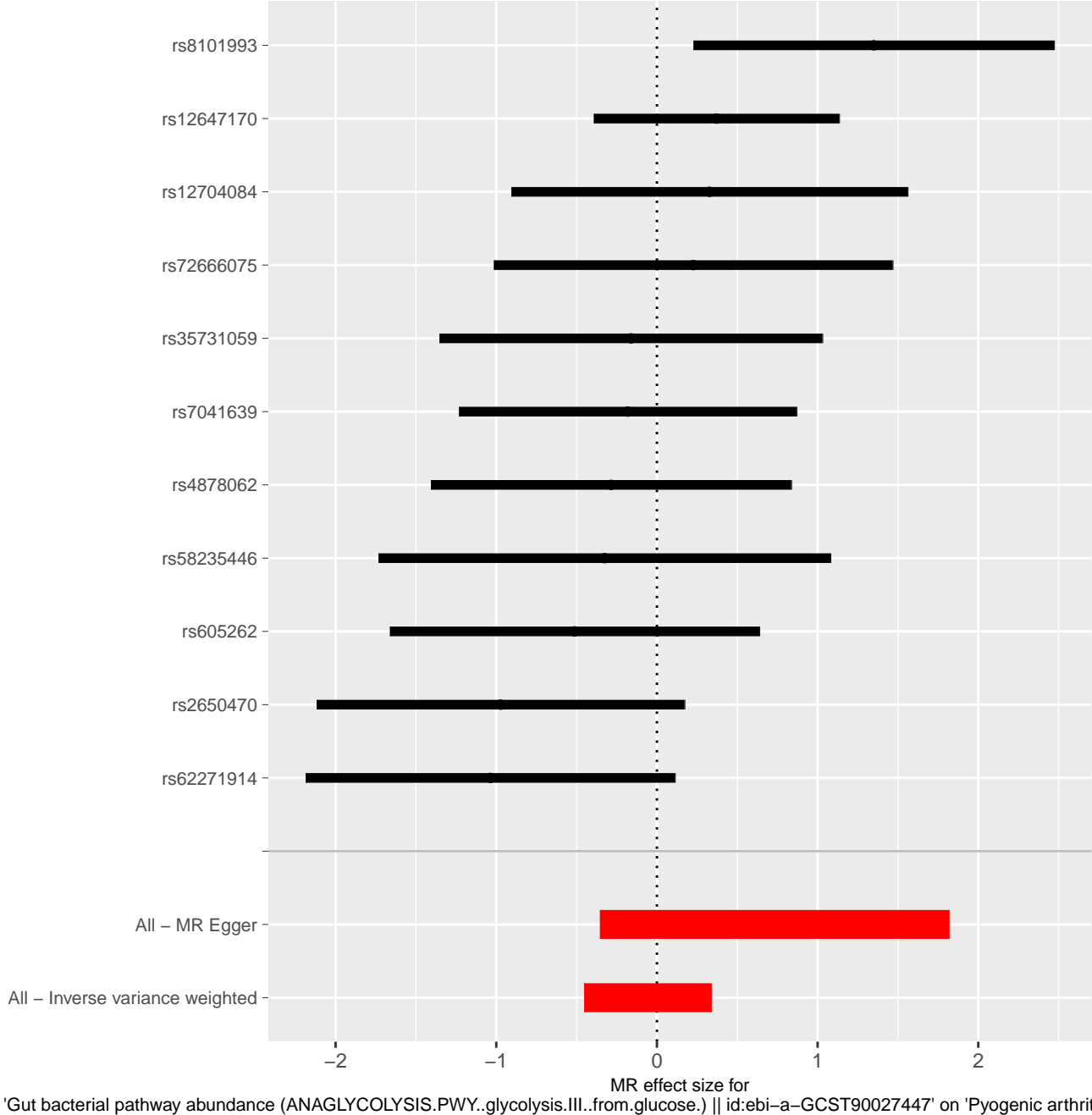

Supplement: Supplementary file 1 [file DataSheet1.zip › mendelian test/ebi-a-GCST90027447.csv_forest.pdf]

# MR Method

- Inverse variance weighted
- MR Egger

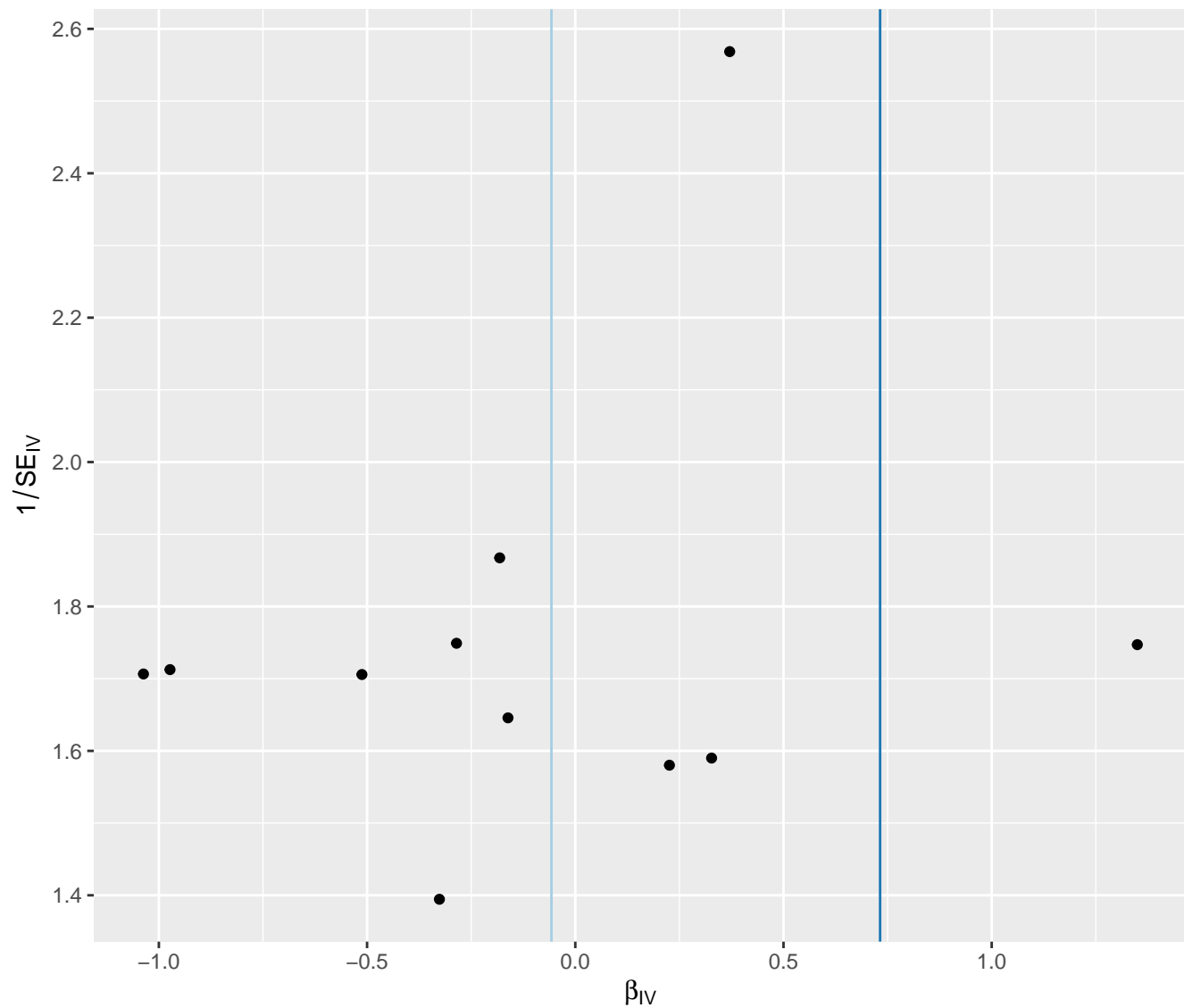

Supplement: Supplementary file 1 [file DataSheet1.zip › mendelian test/ebi-a-GCST90027447.csv_funnel.pdf]

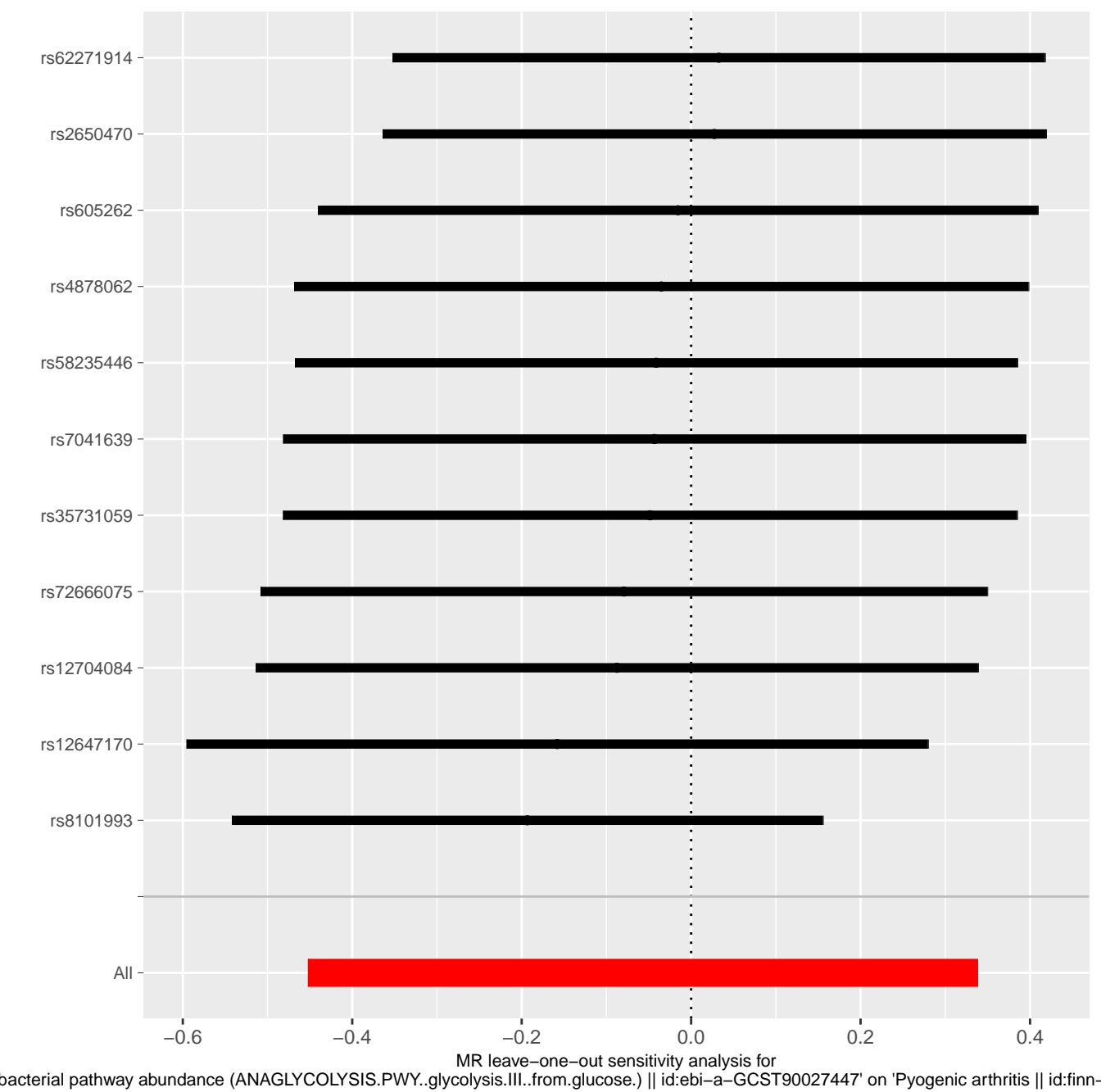

Supplement: Supplementary file 1 [file DataSheet1.zip › mendelian test/ebi-a-GCST90027447.csv_leave_one_out.pdf]

# MR Method

- Inverse variance weighted
- MR Egger

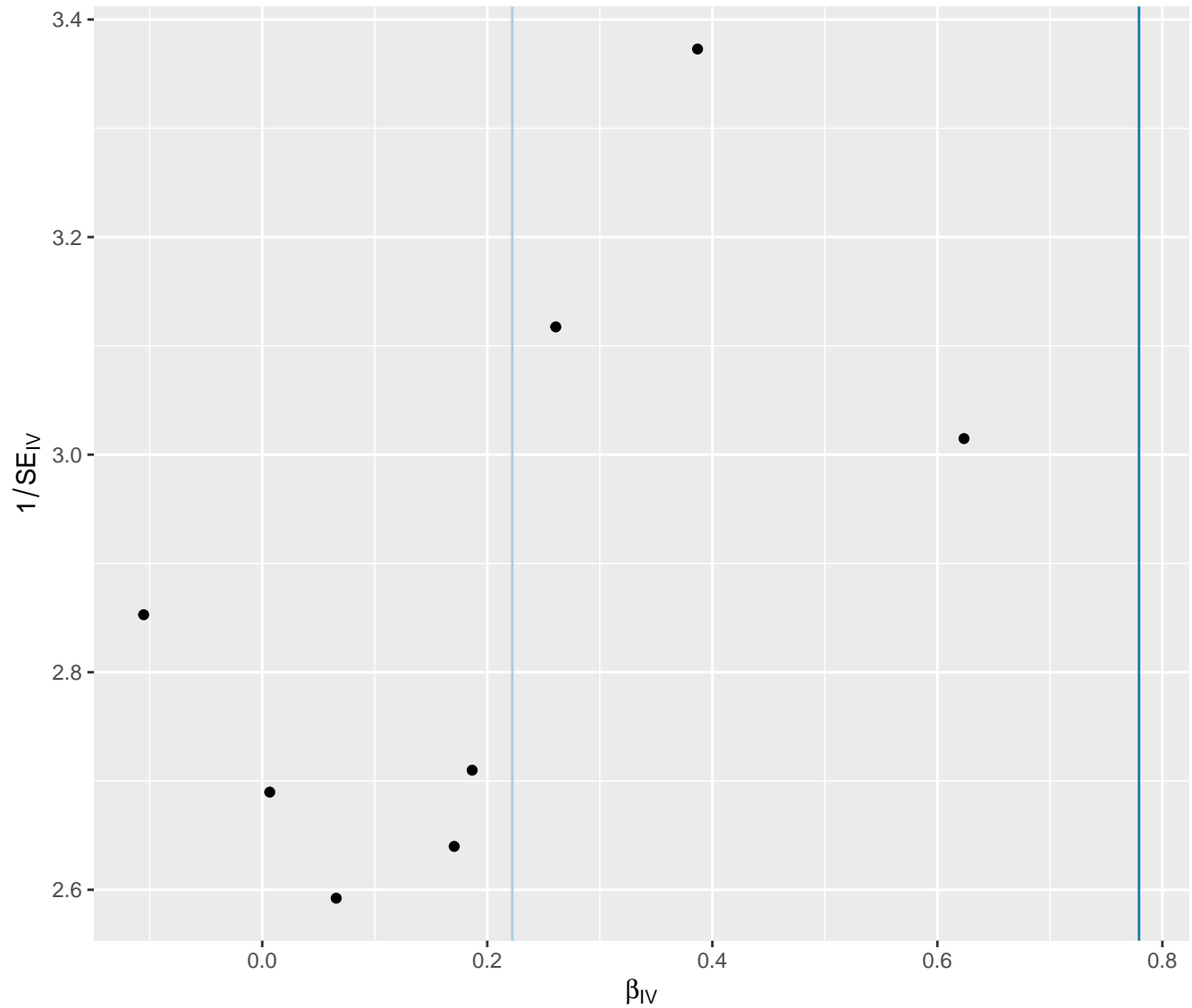

Supplement: Supplementary file 1 [file DataSheet1.zip › mendelian test/ebi-a-GCST90027449.csv_funnel.pdf]

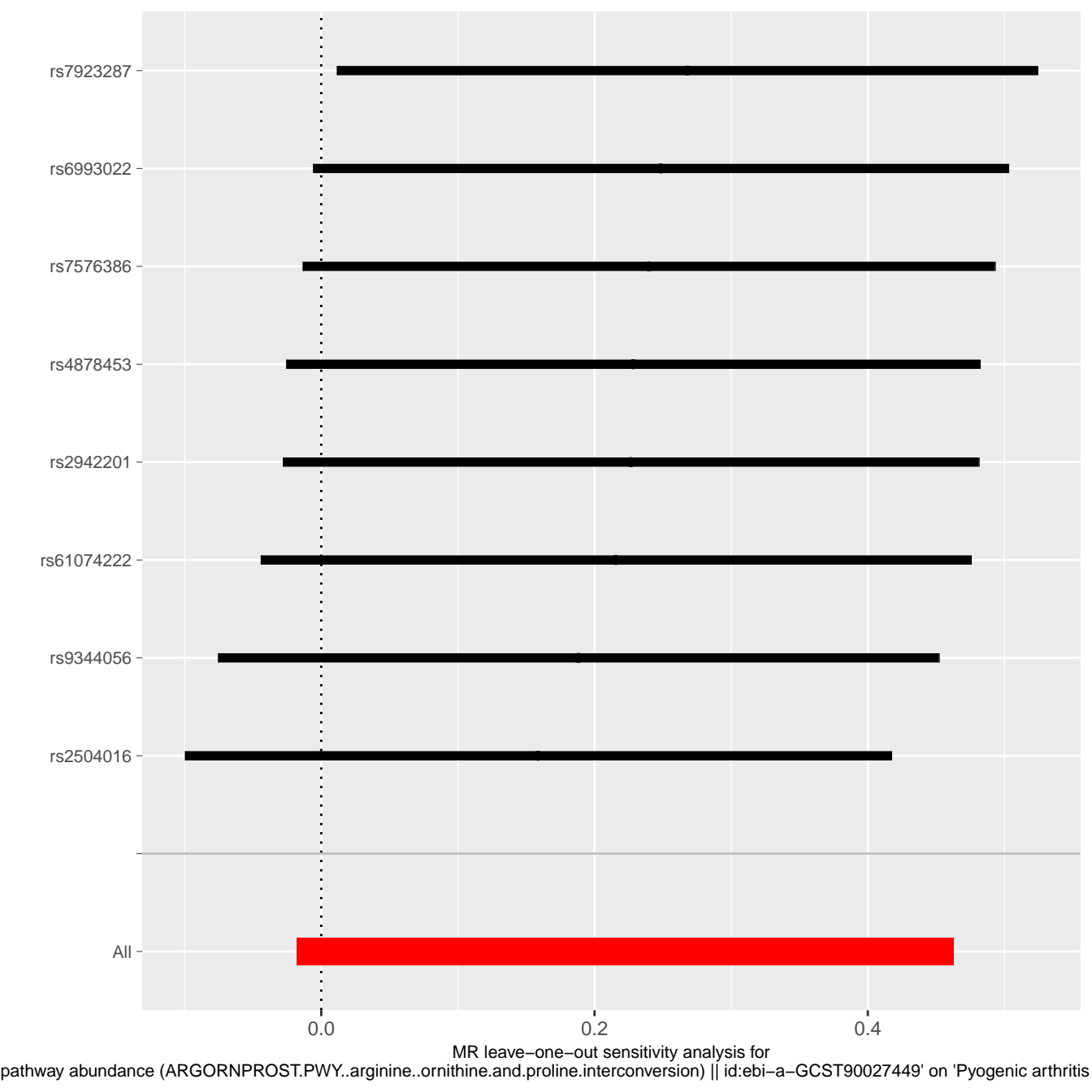

Supplement: Supplementary file 1 [file DataSheet1.zip › mendelian test/ebi-a-GCST90027449.csv_leave_one_out.pdf]

# MR Test

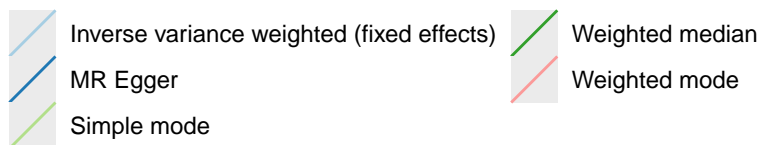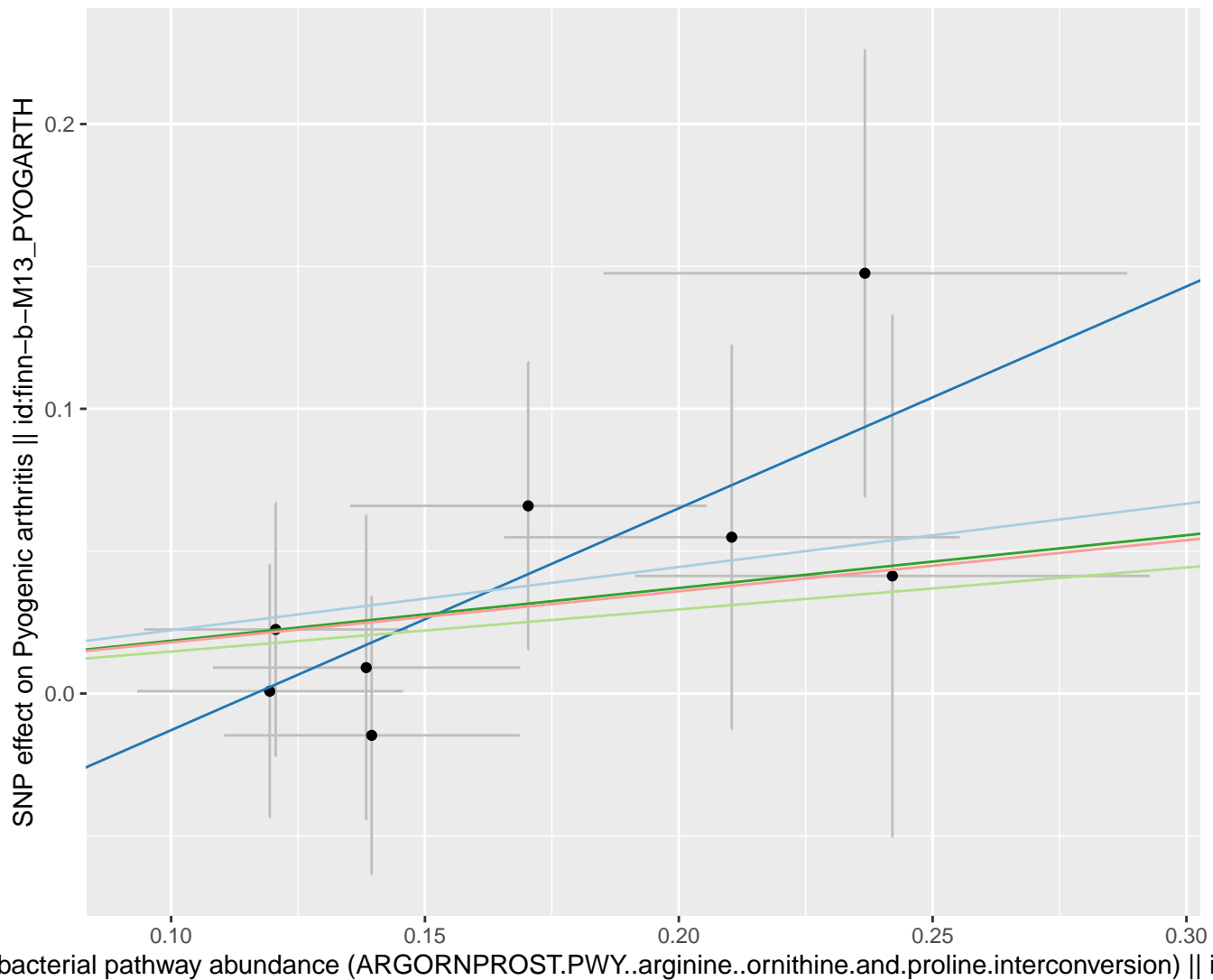

Supplement: Supplementary file 1 [file DataSheet1.zip › mendelian test/ebi-a-GCST90027449.csv_scatter.pdf]

# MR Method

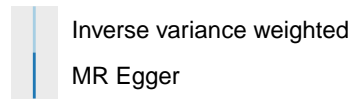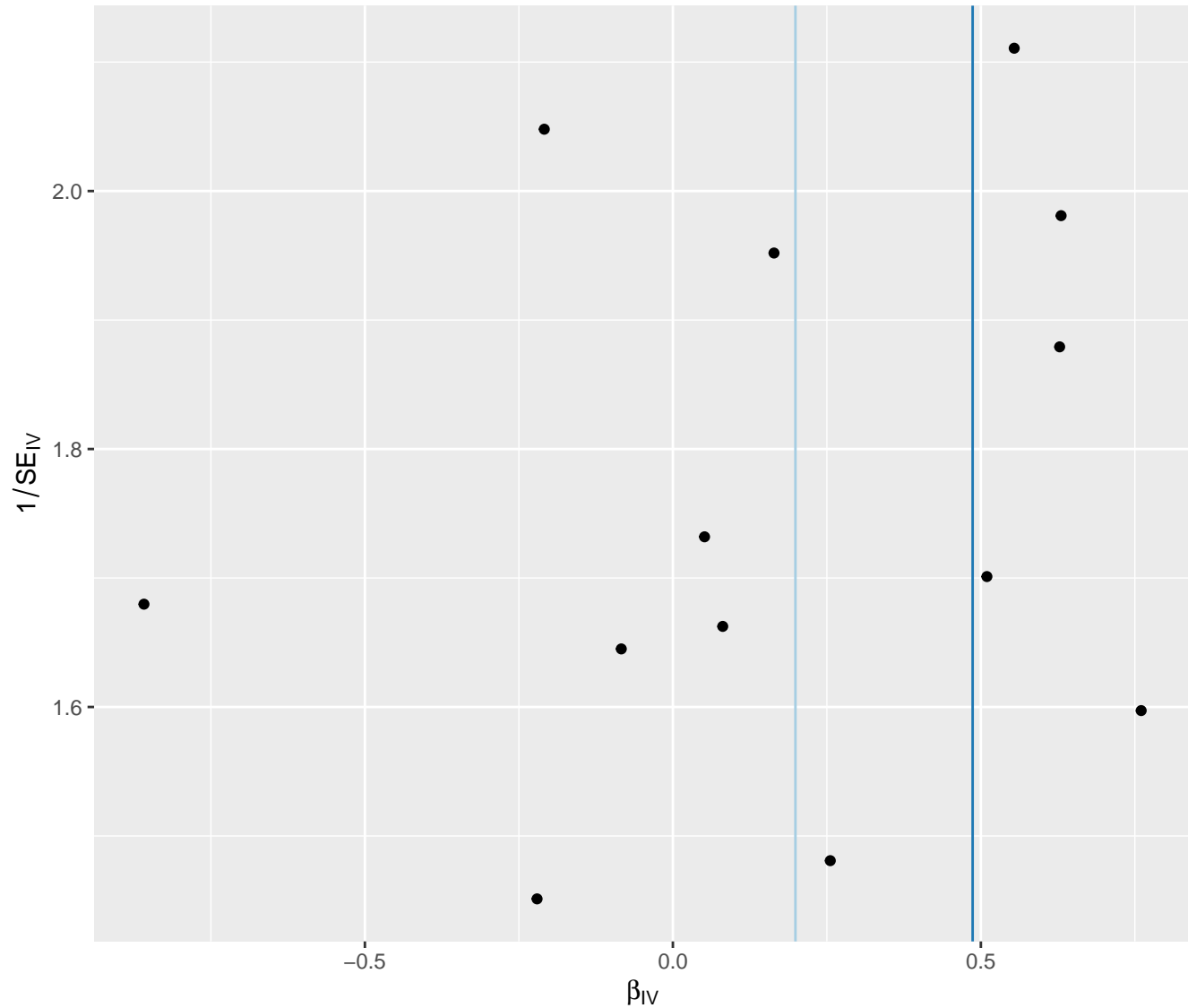

Supplement: Supplementary file 1 [file DataSheet1.zip › mendelian test/ebi-a-GCST90027450.csv_funnel.pdf]

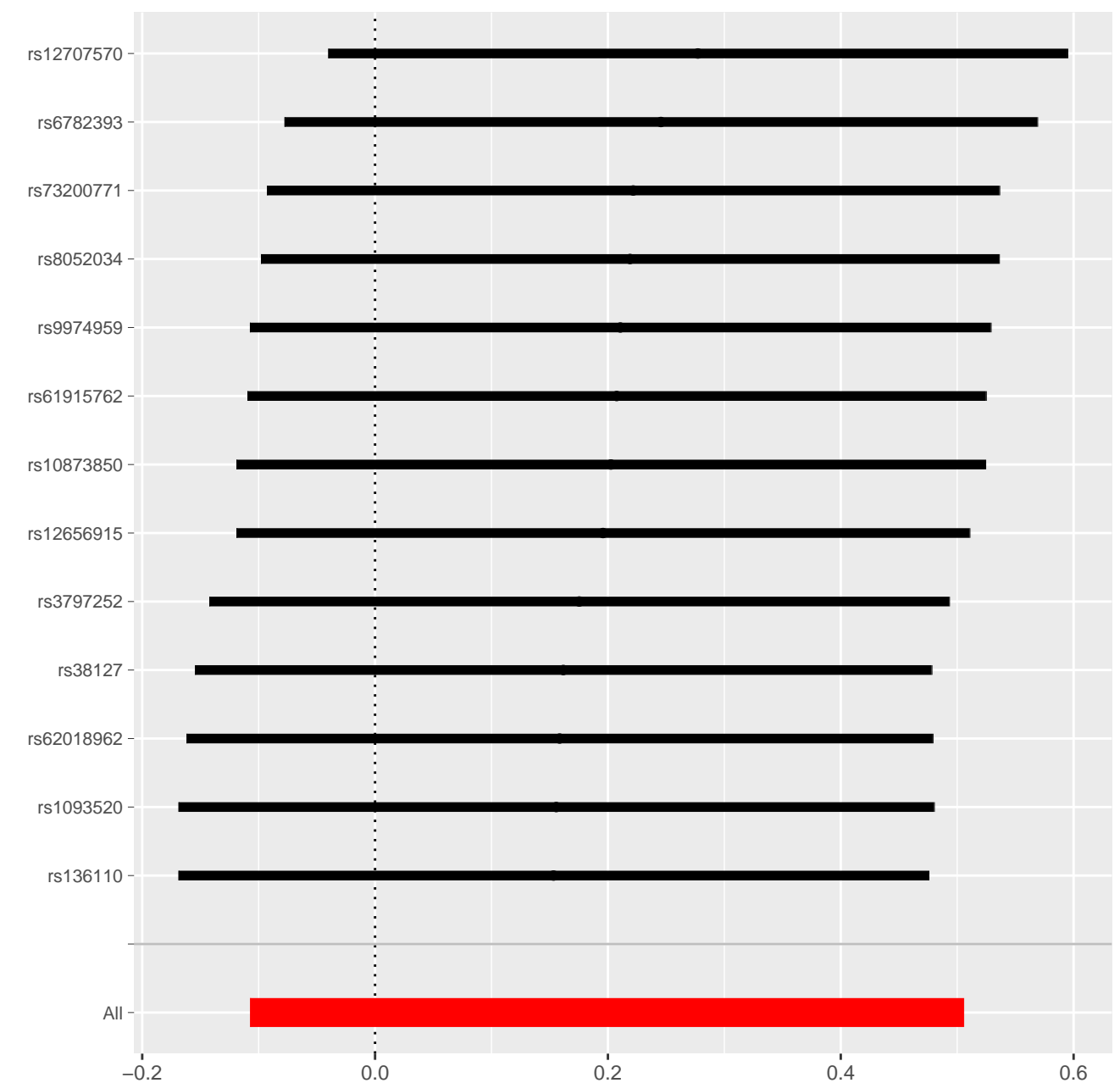

Supplement: Supplementary file 1 [file DataSheet1.zip › mendelian test/ebi-a-GCST90027450.csv_leave_one_out.pdf]

# MR Test

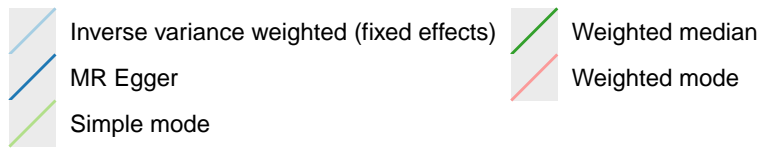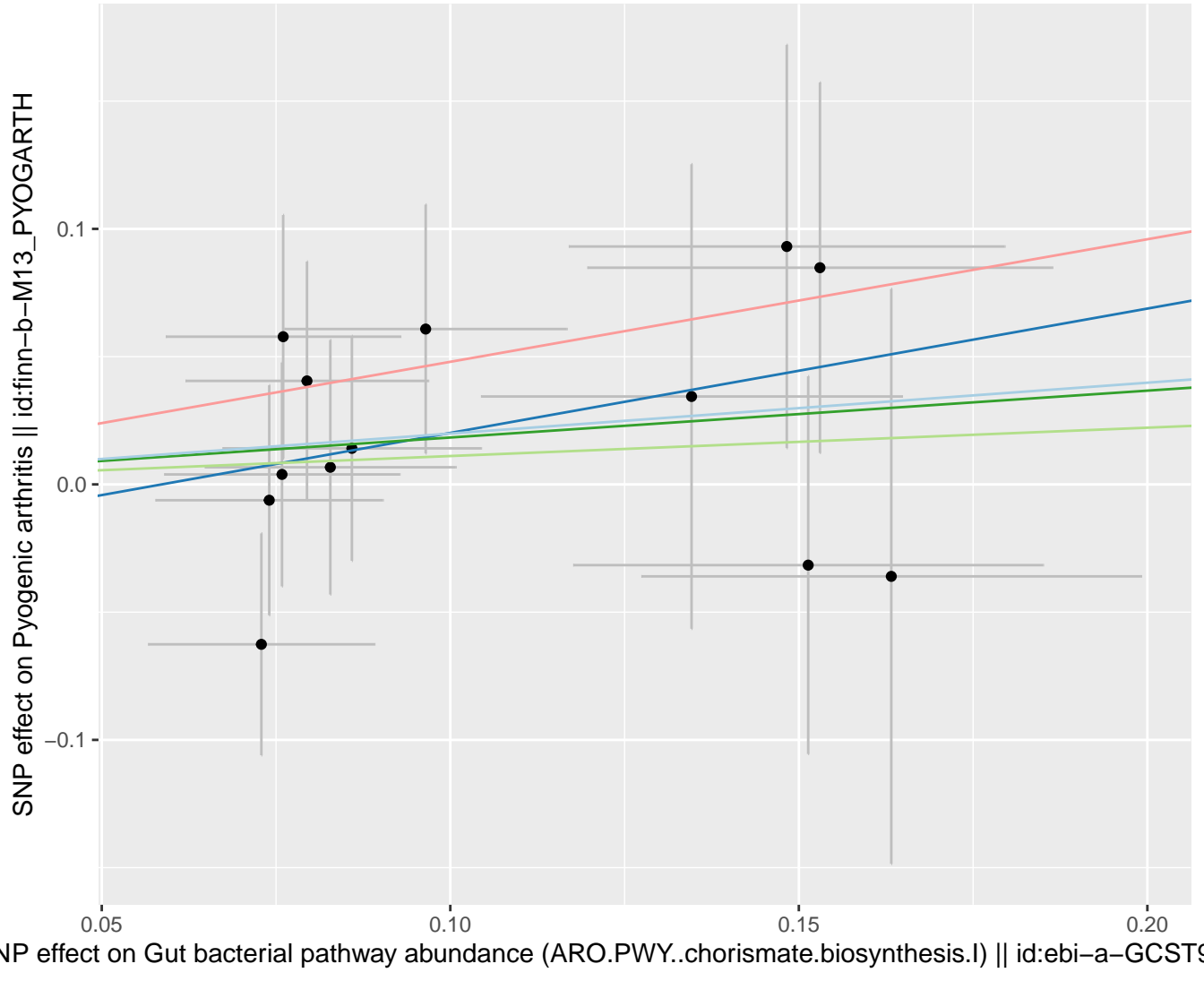

Supplement: Supplementary file 1 [file DataSheet1.zip › mendelian test/ebi-a-GCST90027450.csv_scatter.pdf]

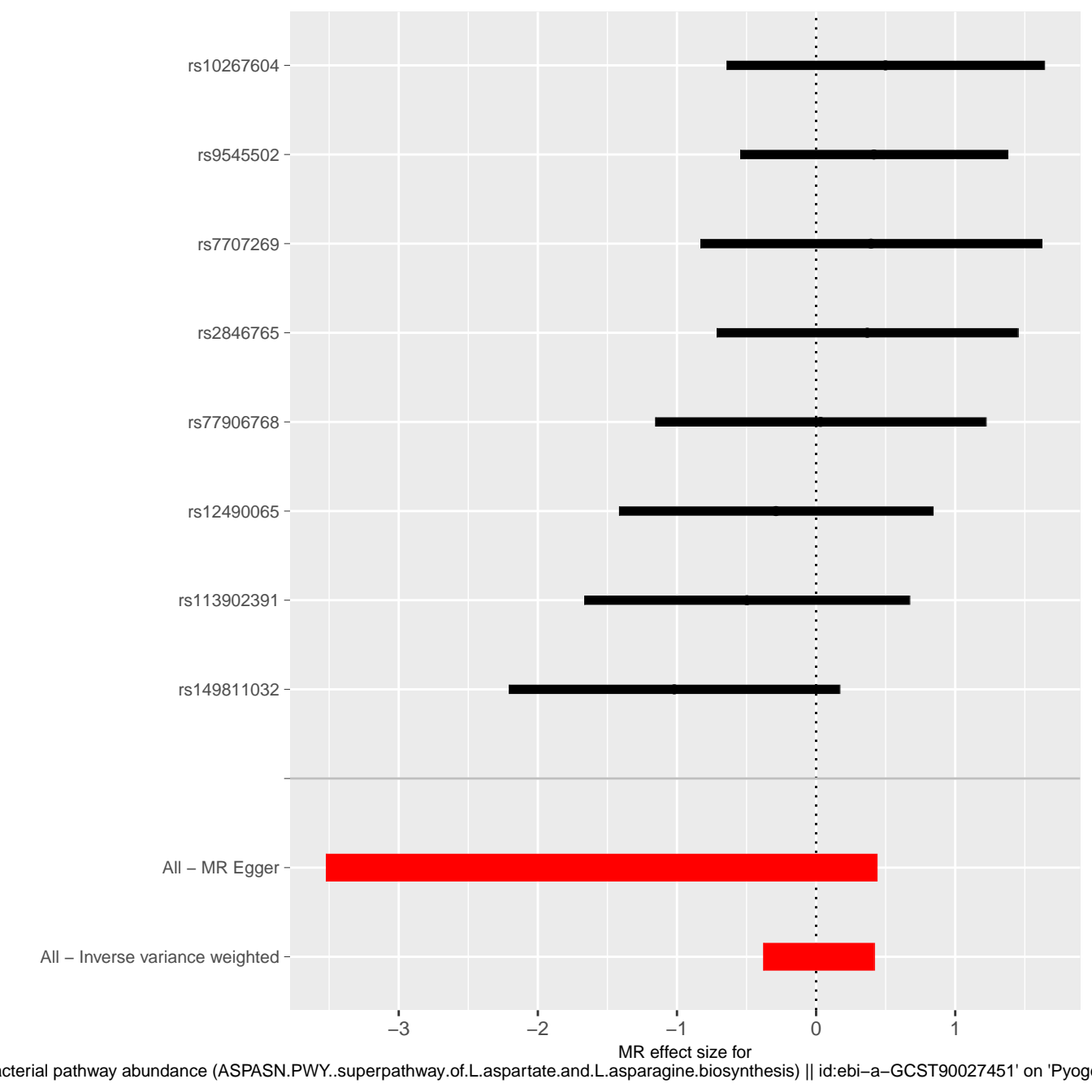

Supplement: Supplementary file 1 [file DataSheet1.zip › mendelian test/ebi-a-GCST90027451.csv_forest.pdf]

# MR Method

- Inverse variance weighted
- MR Egger

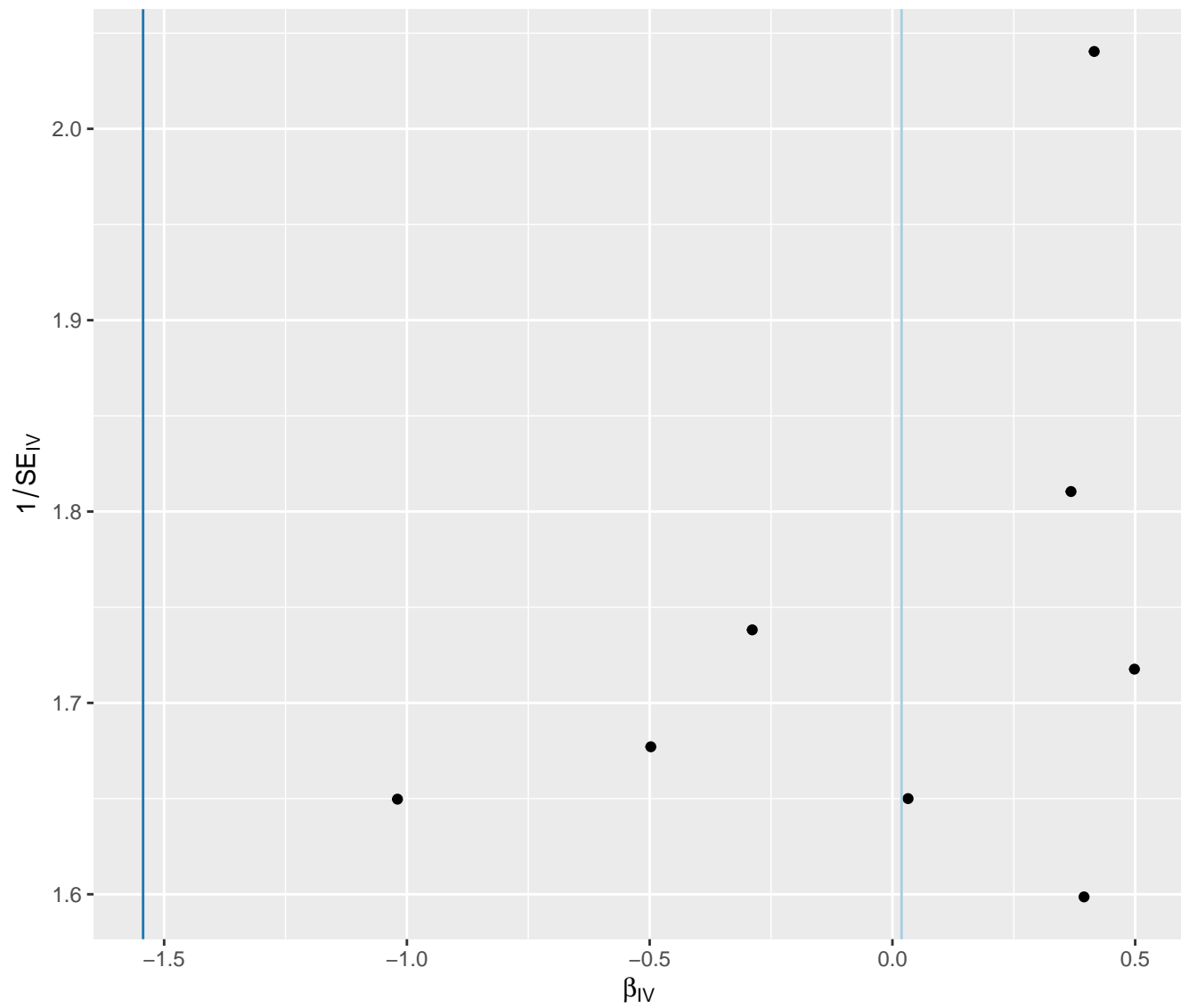

Supplement: Supplementary file 1 [file DataSheet1.zip › mendelian test/ebi-a-GCST90027451.csv_funnel.pdf]

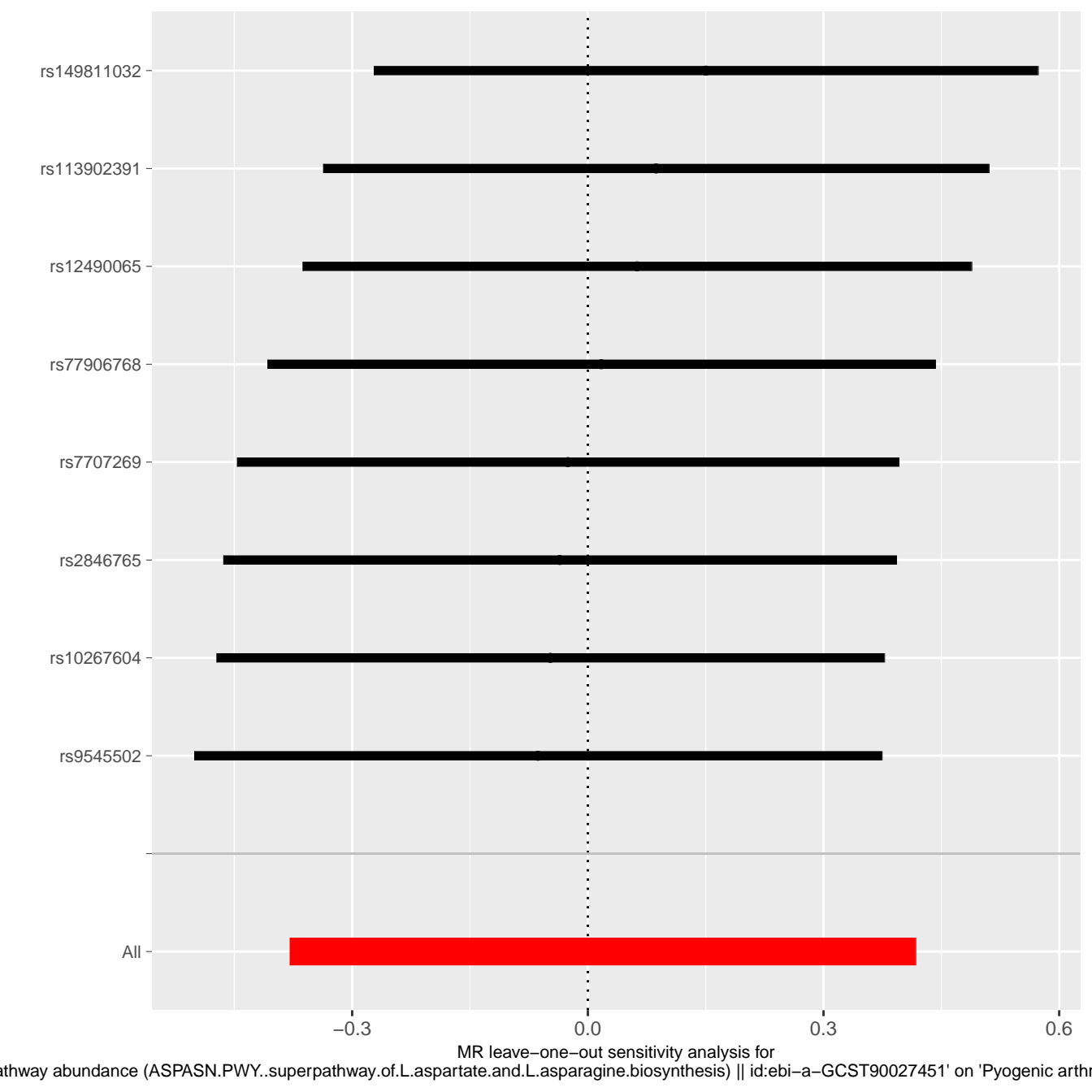

Supplement: Supplementary file 1 [file DataSheet1.zip › mendelian test/ebi-a-GCST90027451.csv_leave_one_out.pdf]

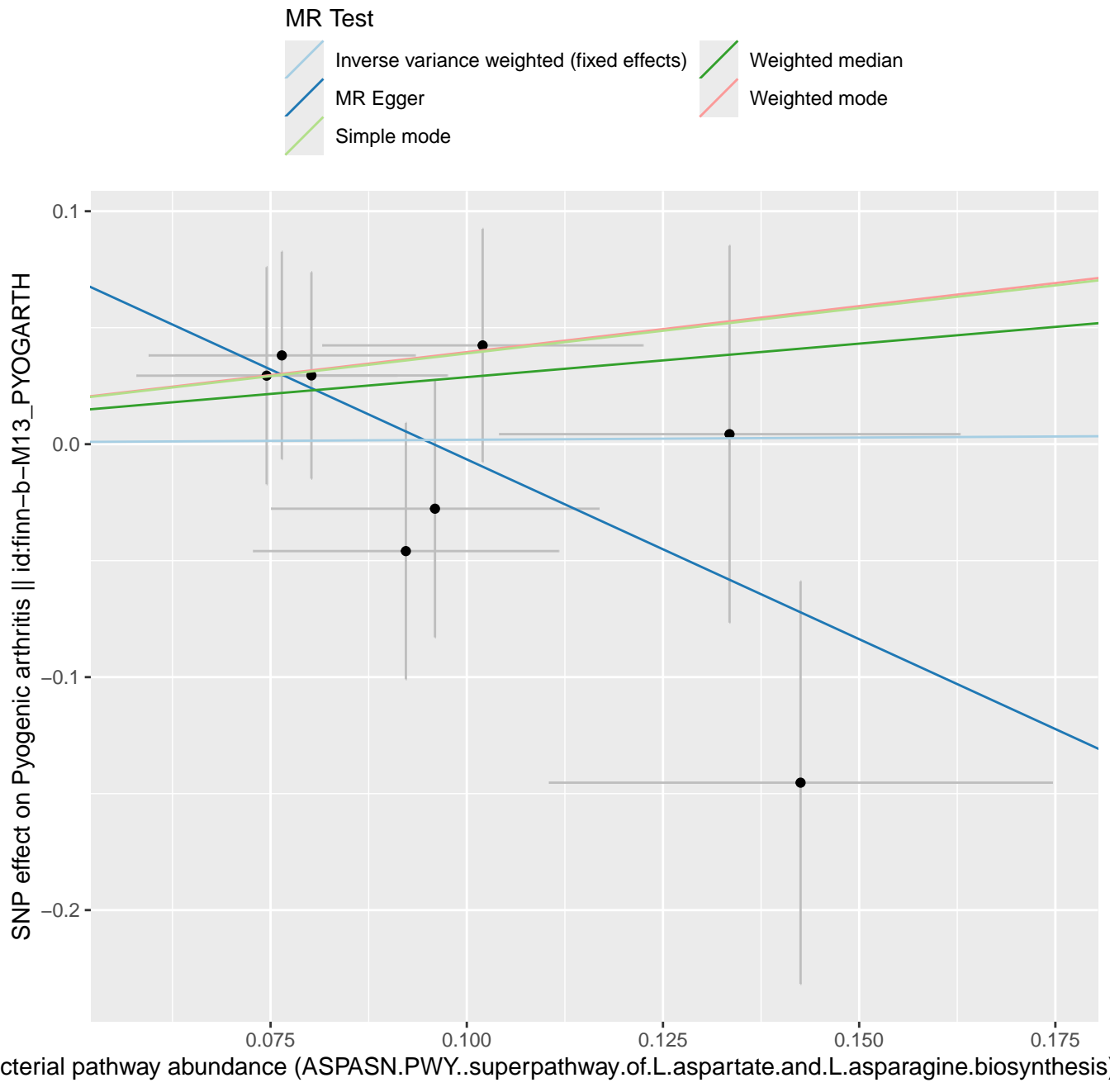

Supplement: Supplementary file 1 [file DataSheet1.zip › mendelian test/ebi-a-GCST90027451.csv_scatter.pdf]

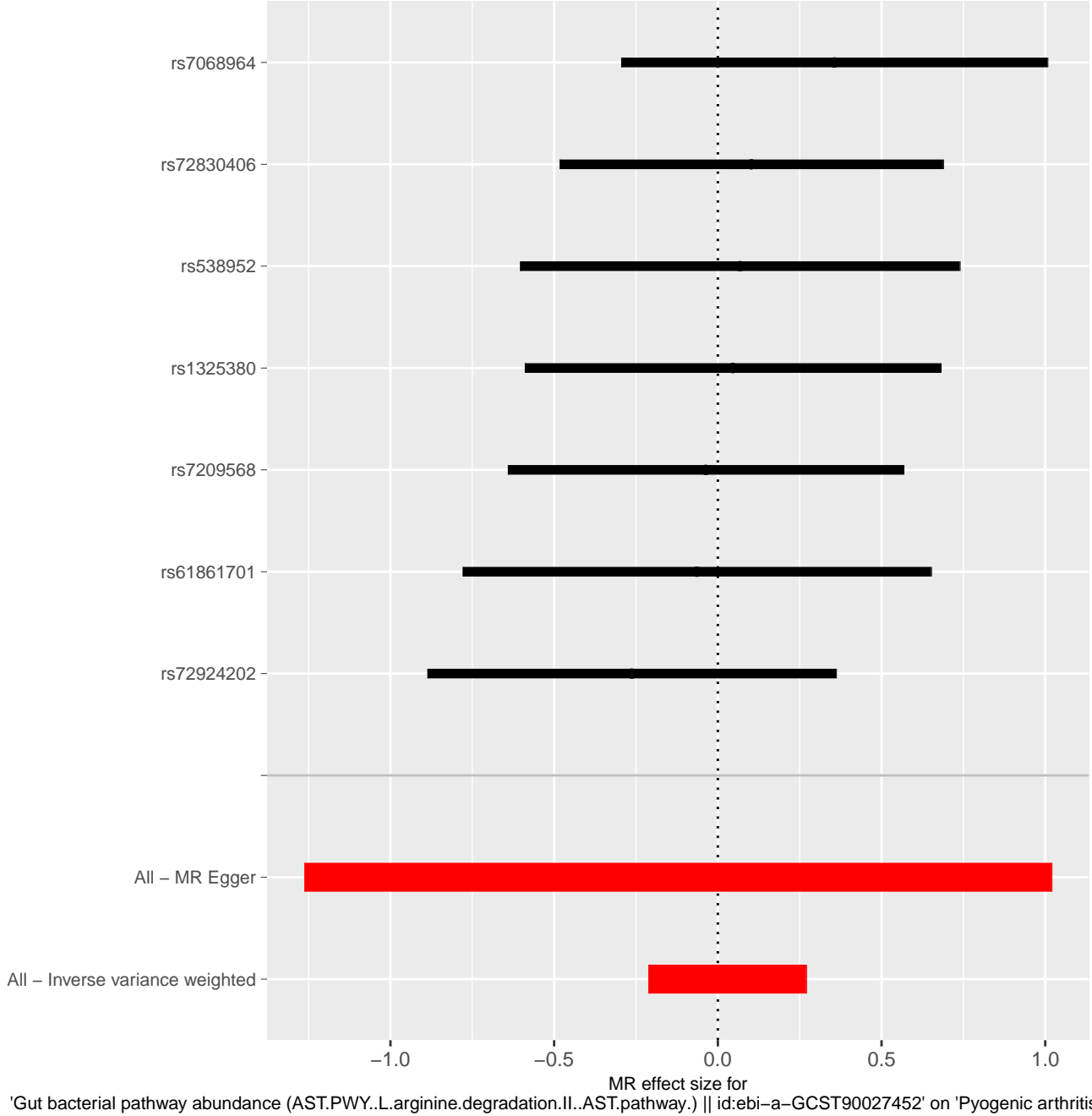

Supplement: Supplementary file 1 [file DataSheet1.zip › mendelian test/ebi-a-GCST90027452.csv_forest.pdf]

# MR Method

- Inverse variance weighted
- MR Egger

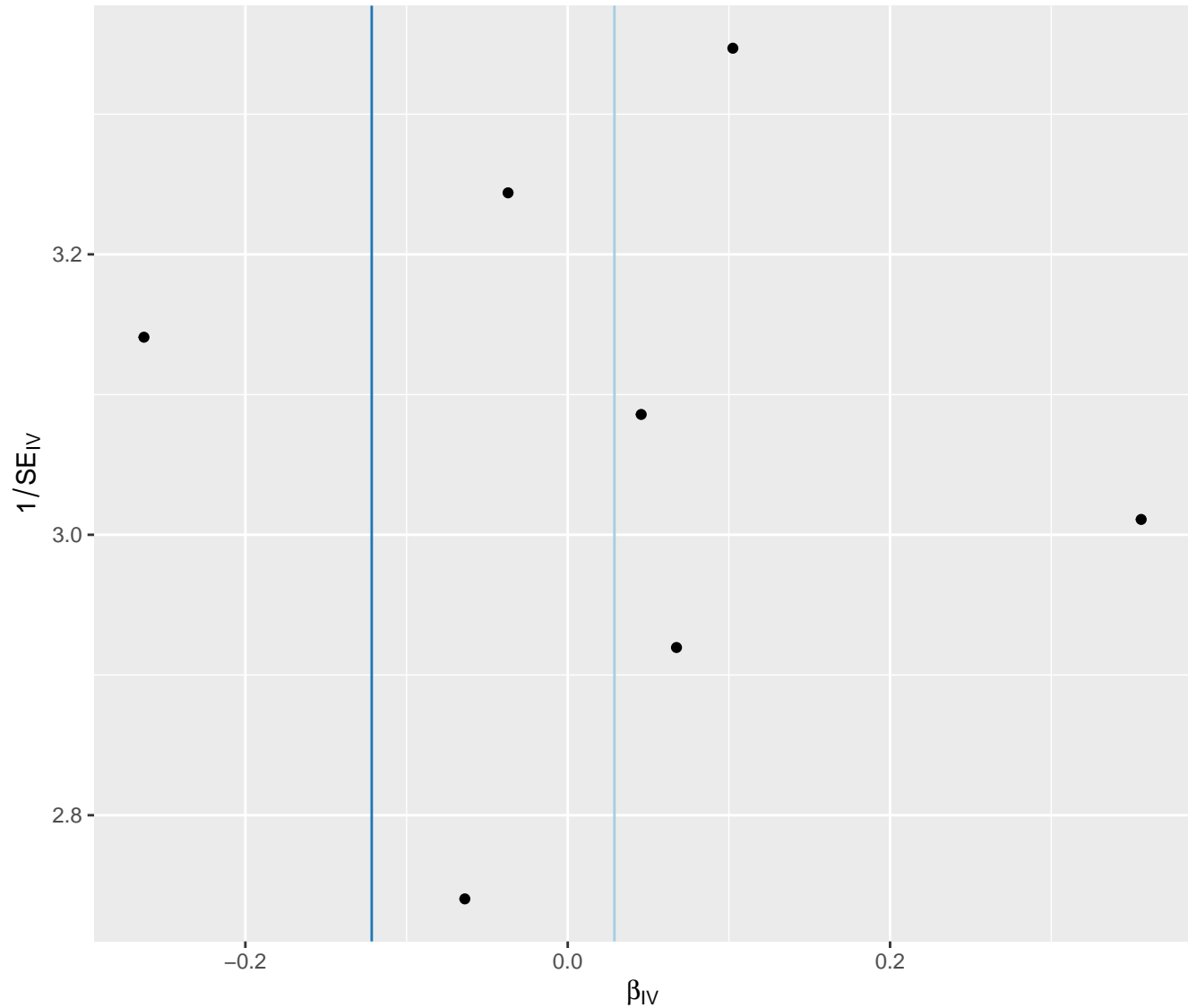

Supplement: Supplementary file 1 [file DataSheet1.zip › mendelian test/ebi-a-GCST90027452.csv_funnel.pdf]

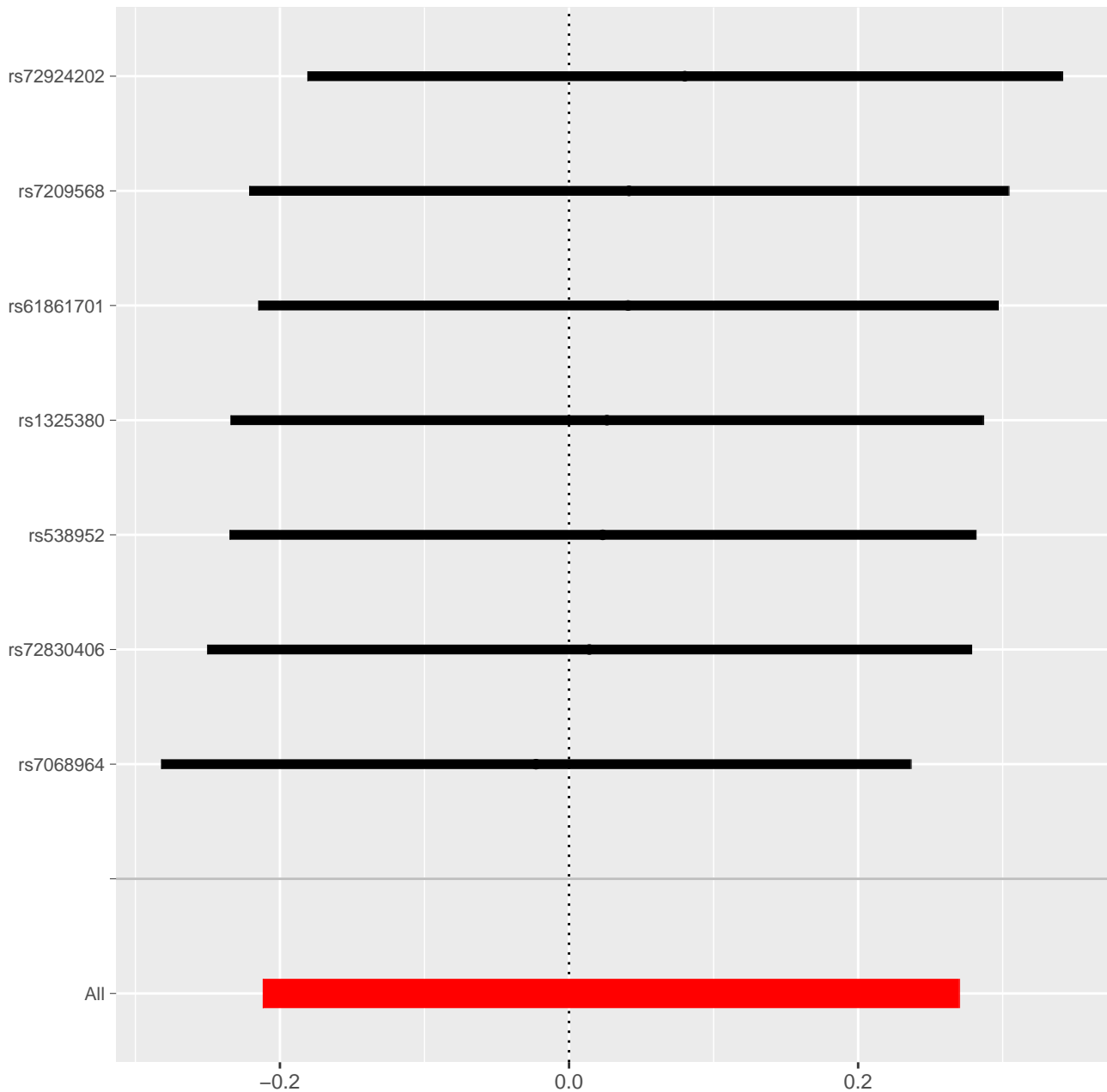

Supplement: Supplementary file 1 [file DataSheet1.zip › mendelian test/ebi-a-GCST90027452.csv_leave_one_out.pdf]

# MR Method

- Inverse variance weighted
- MR Egger

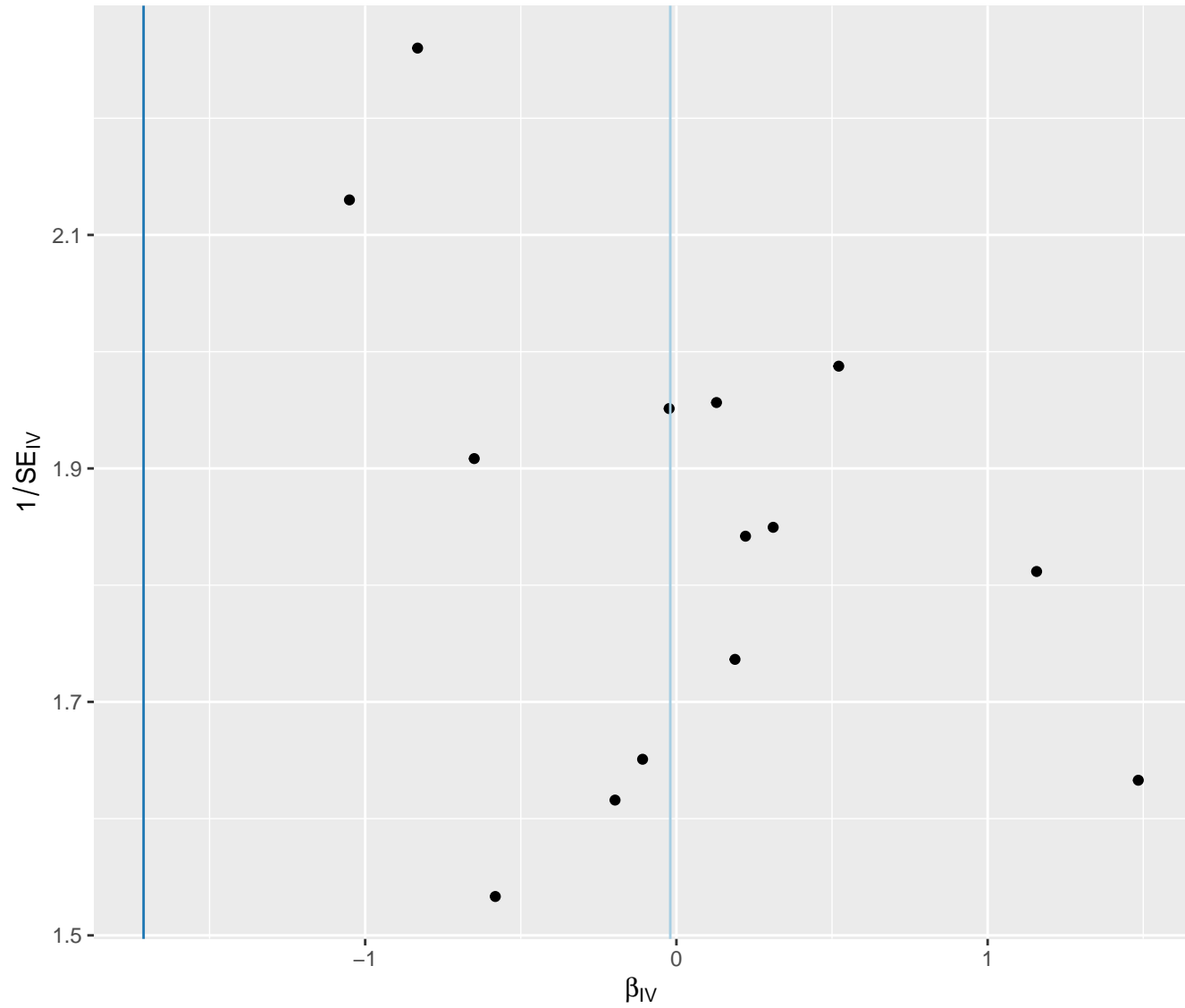

Supplement: Supplementary file 1 [file DataSheet1.zip › mendelian test/ebi-a-GCST90027453.csv_funnel.pdf]

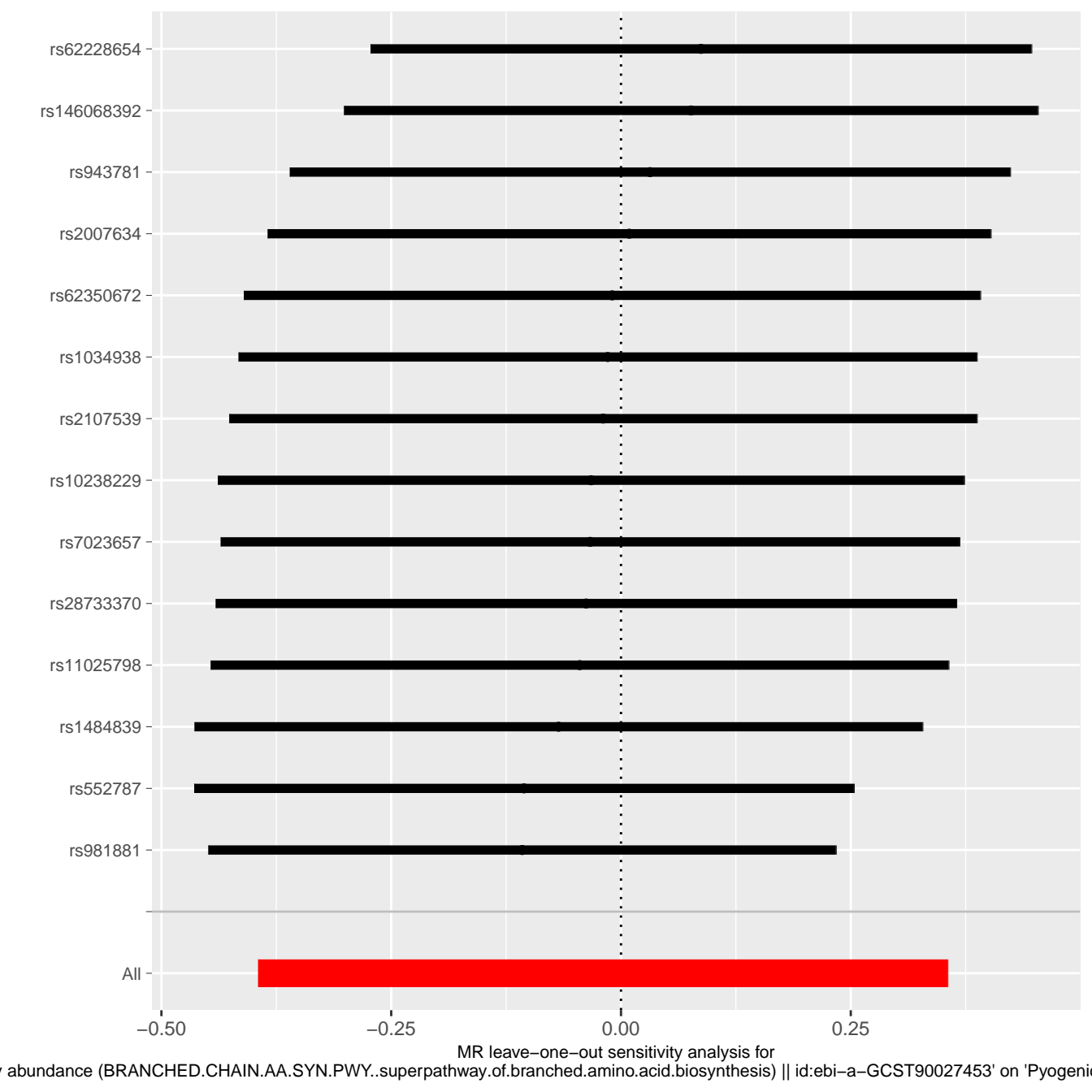

Supplement: Supplementary file 1 [file DataSheet1.zip › mendelian test/ebi-a-GCST90027453.csv_leave_one_out.pdf]

# MR Test

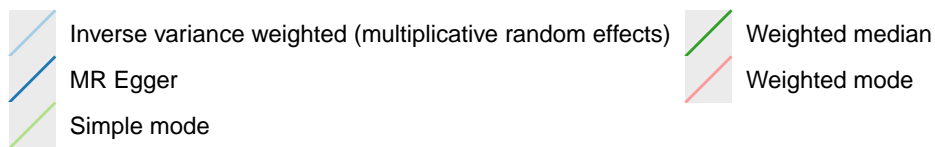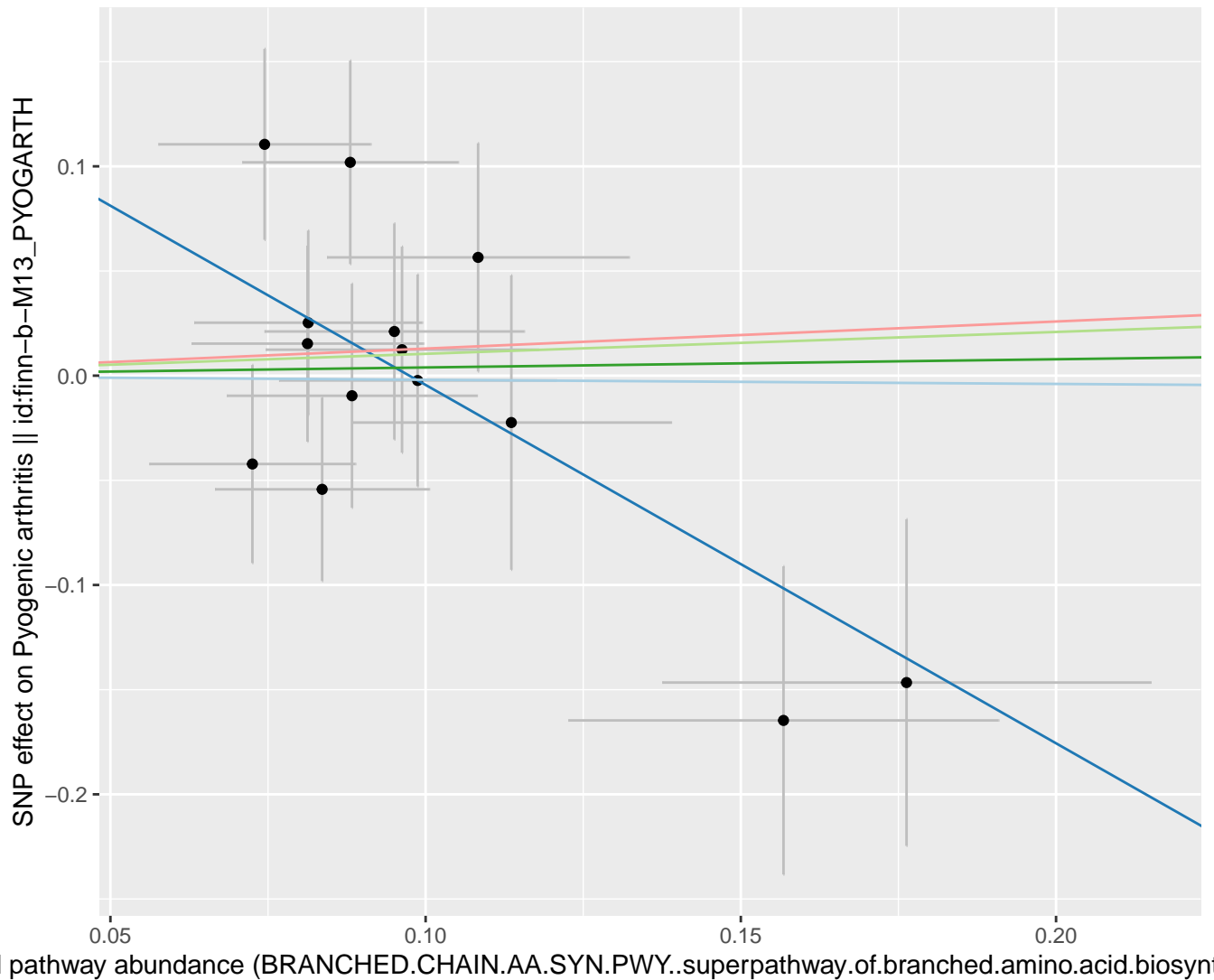

Supplement: Supplementary file 1 [file DataSheet1.zip › mendelian test/ebi-a-GCST90027453.csv_scatter.pdf]

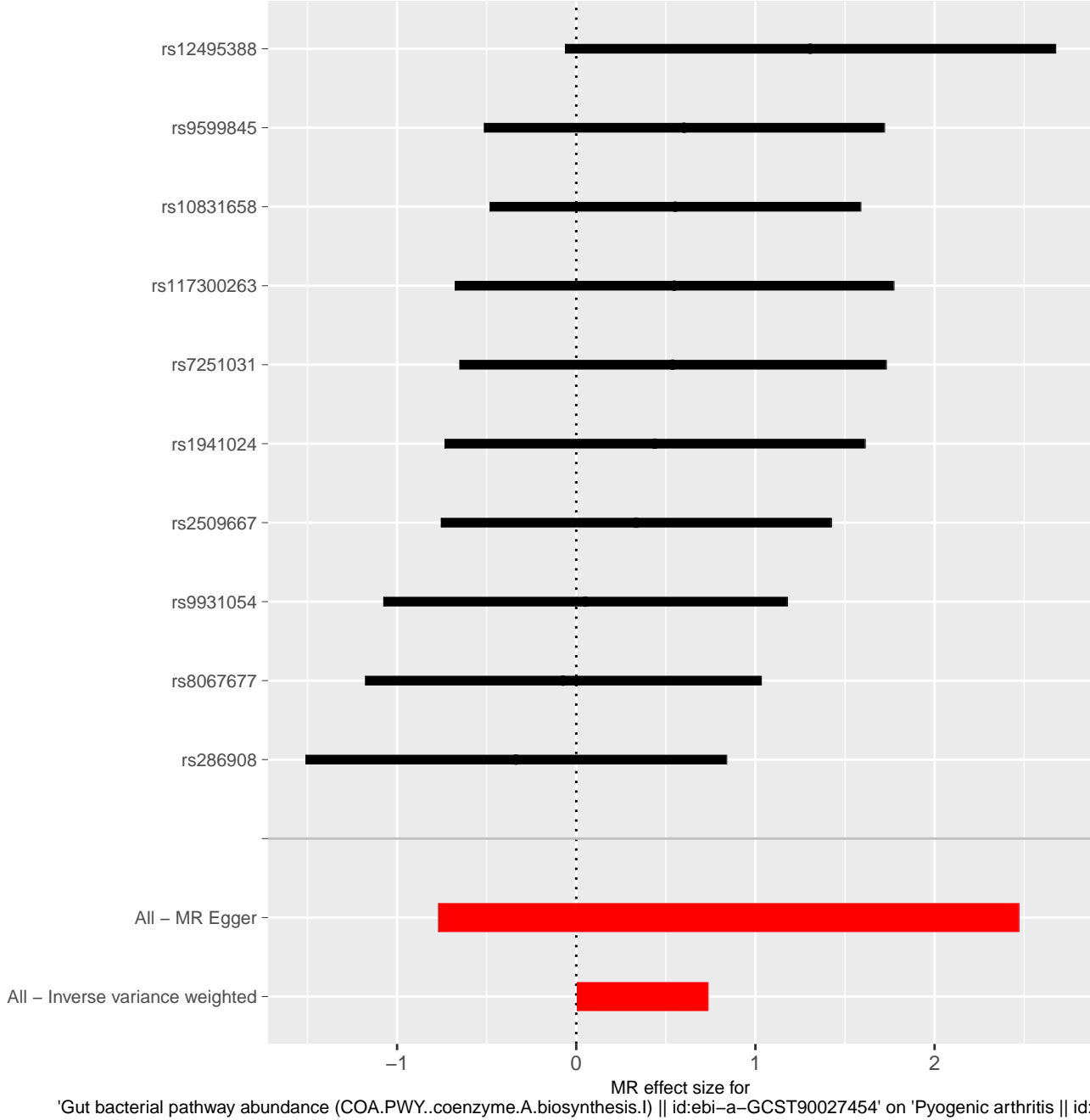

Supplement: Supplementary file 1 [file DataSheet1.zip › mendelian test/ebi-a-GCST90027454.csv_forest.pdf]

# MR Method

- Inverse variance weighted
- MR Egger

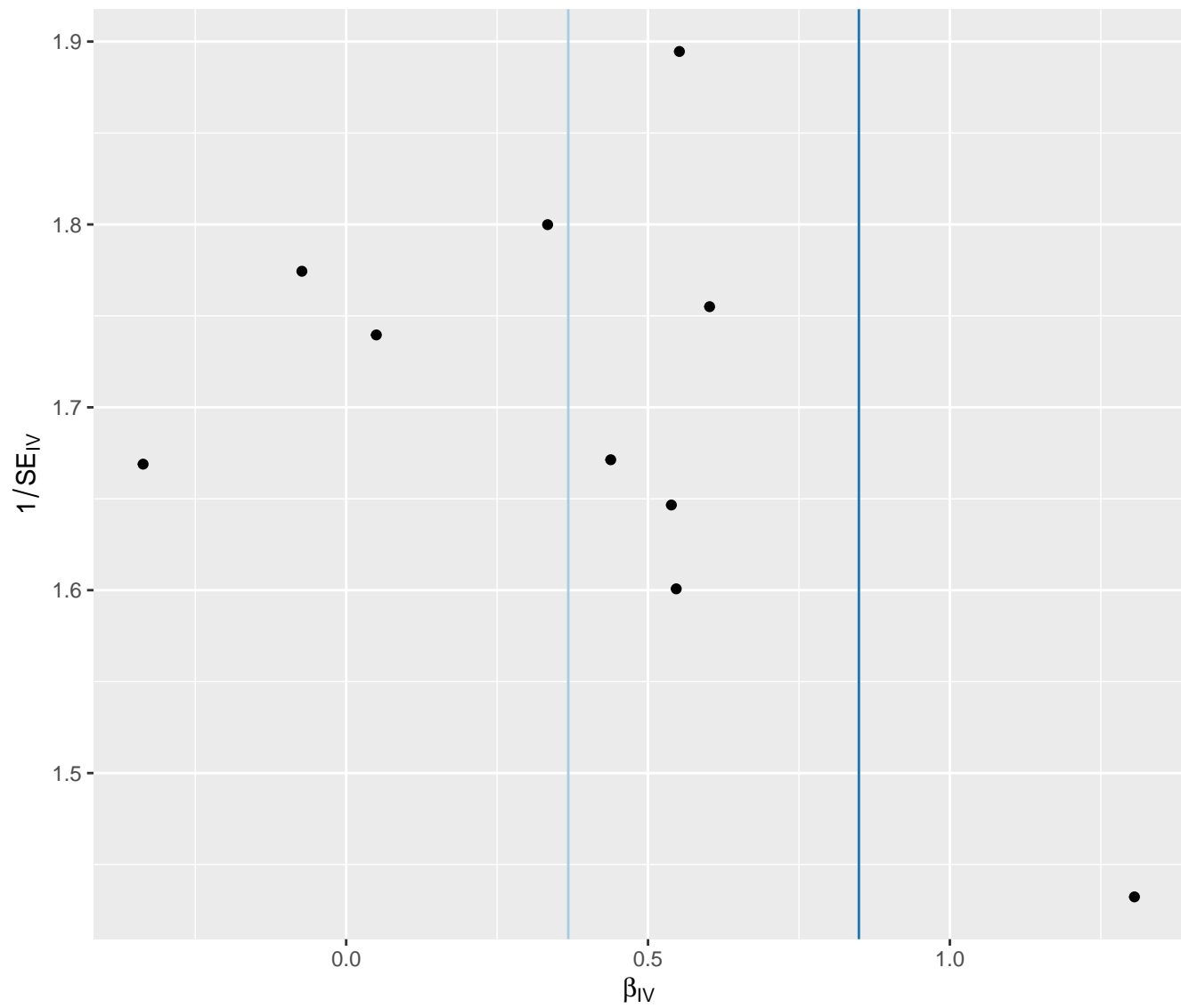

Supplement: Supplementary file 1 [file DataSheet1.zip › mendelian test/ebi-a-GCST90027454.csv_funnel.pdf]

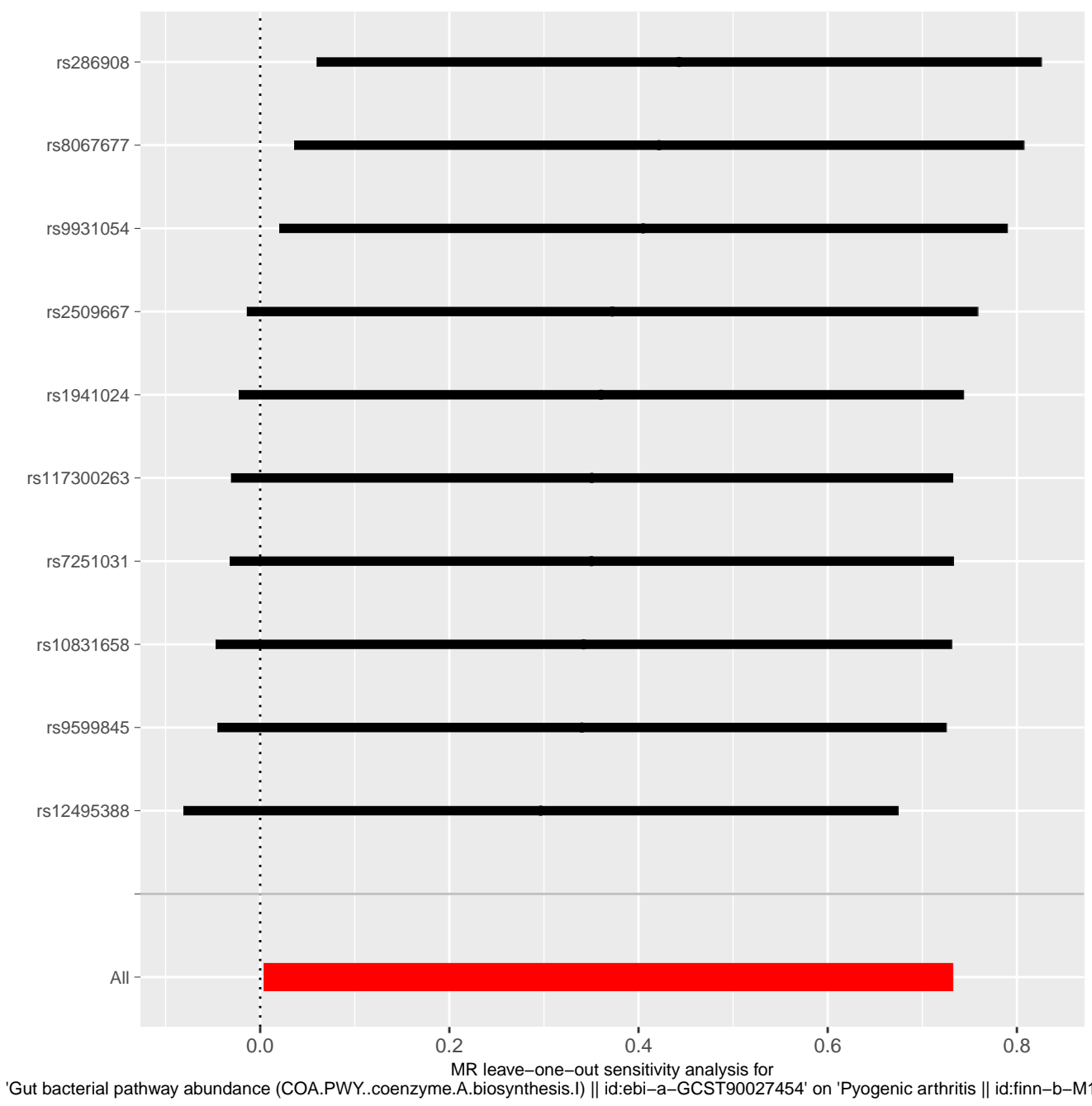

Supplement: Supplementary file 1 [file DataSheet1.zip › mendelian test/ebi-a-GCST90027454.csv_leave_one_out.pdf]

# MR Test

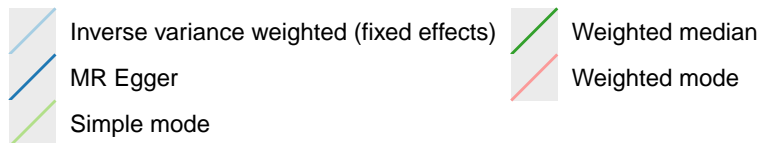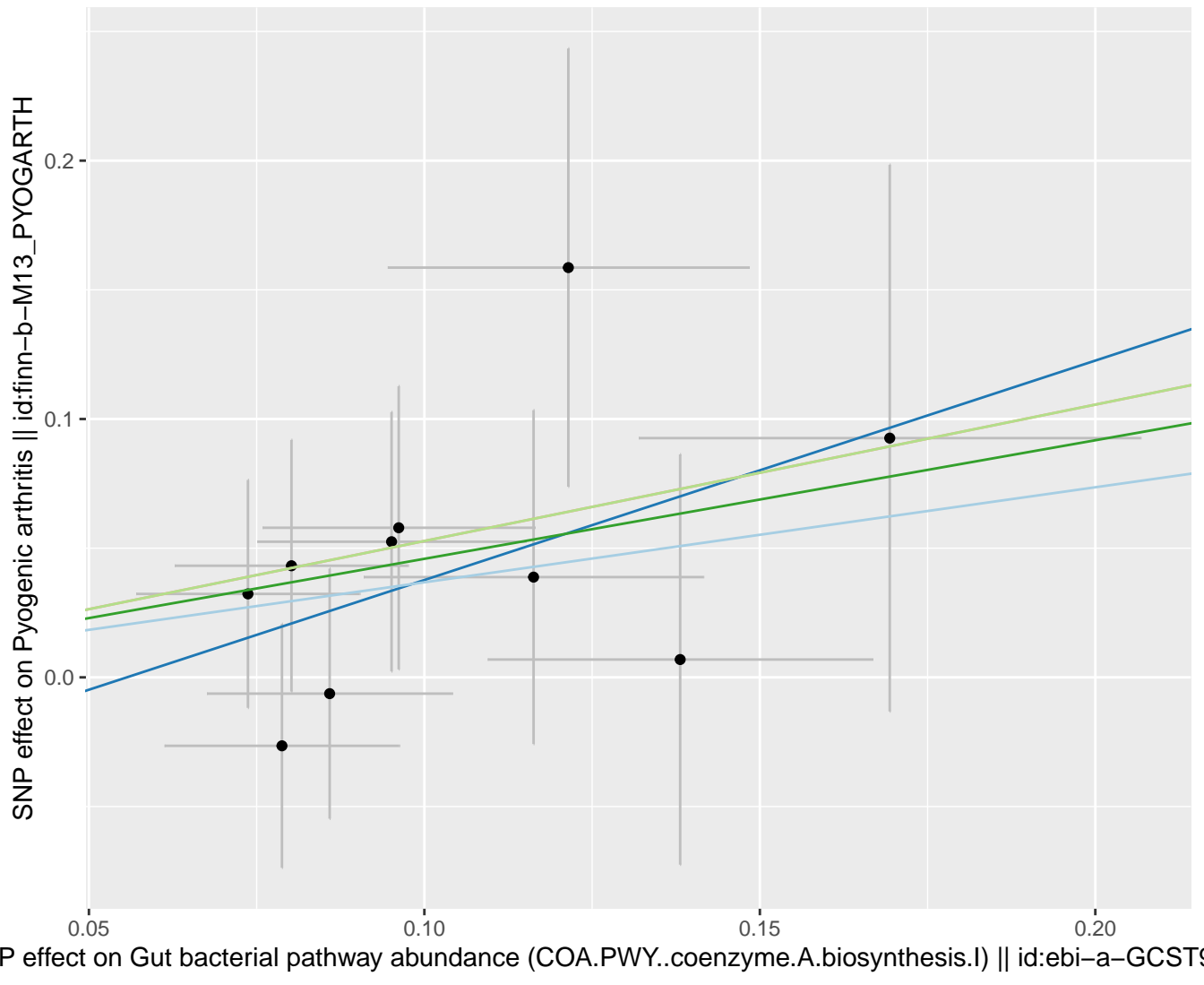

Supplement: Supplementary file 1 [file DataSheet1.zip › mendelian test/ebi-a-GCST90027454.csv_scatter.pdf]

# MR Method

- Inverse variance weighted
- MR Egger

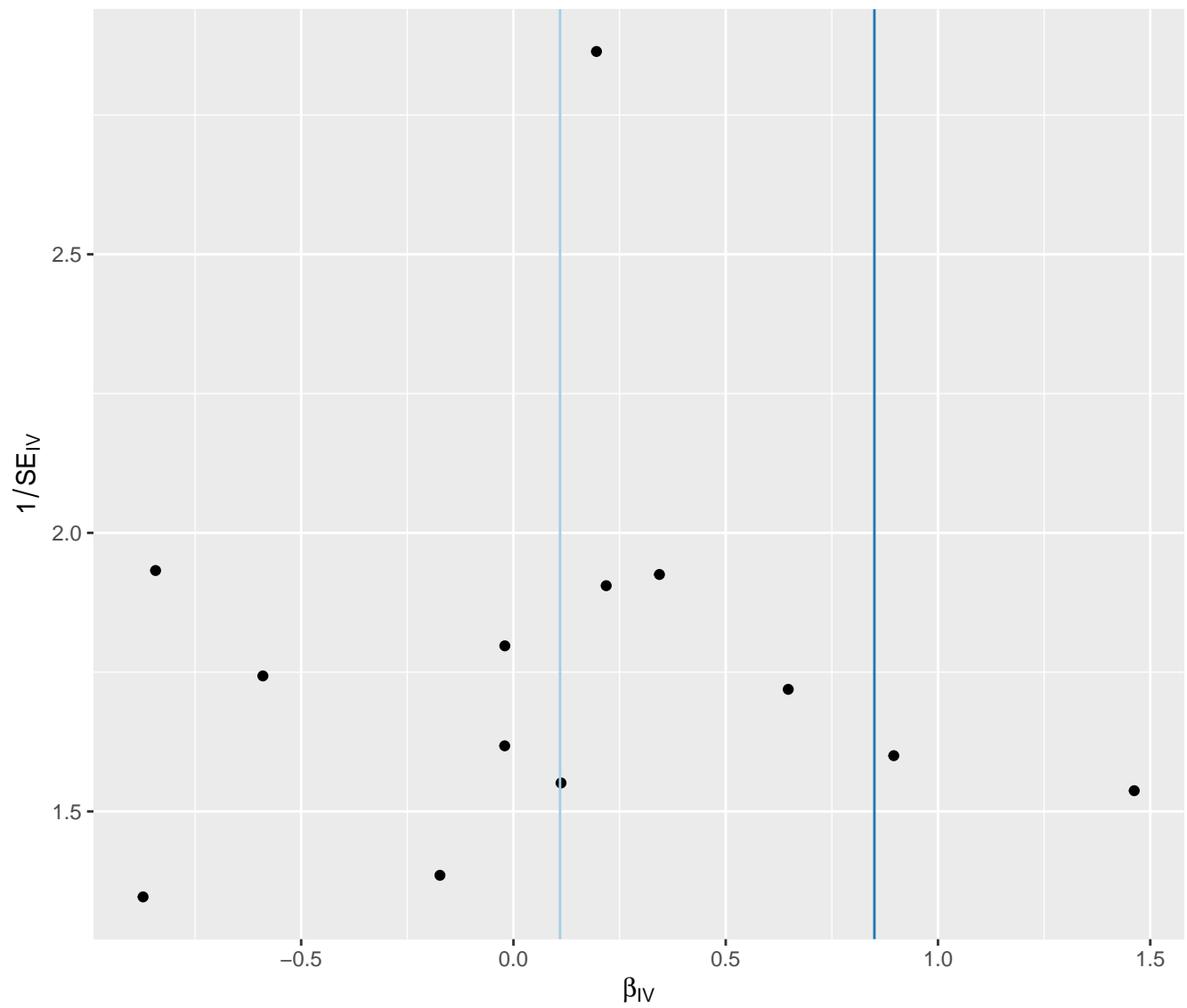

Supplement: Supplementary file 1 [file DataSheet1.zip › mendelian test/ebi-a-GCST90027455.csv_funnel.pdf]

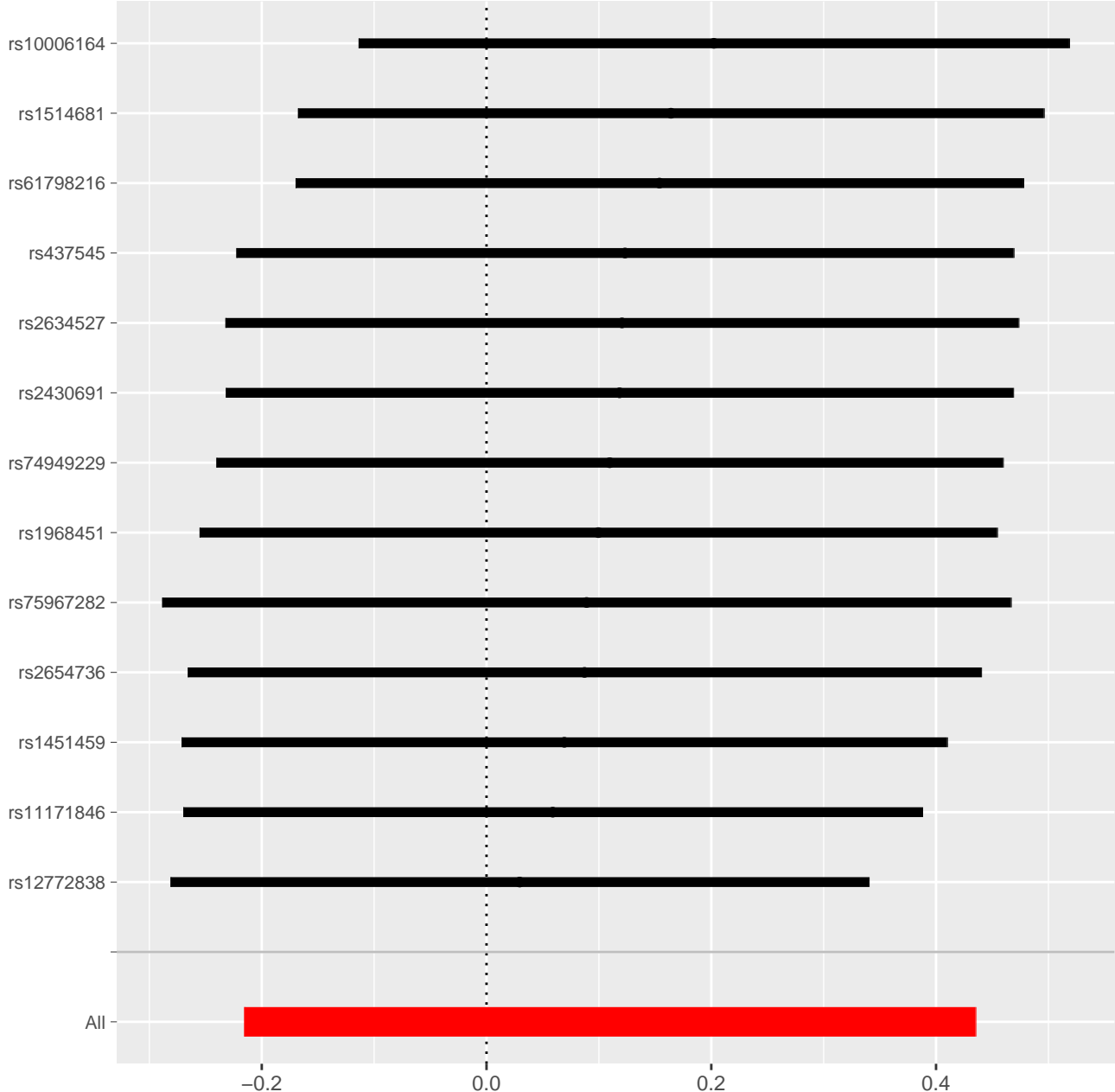

Supplement: Supplementary file 1 [file DataSheet1.zip › mendelian test/ebi-a-GCST90027455.csv_leave_one_out.pdf]

# MR Method

- Inverse variance weighted
- MR Egger

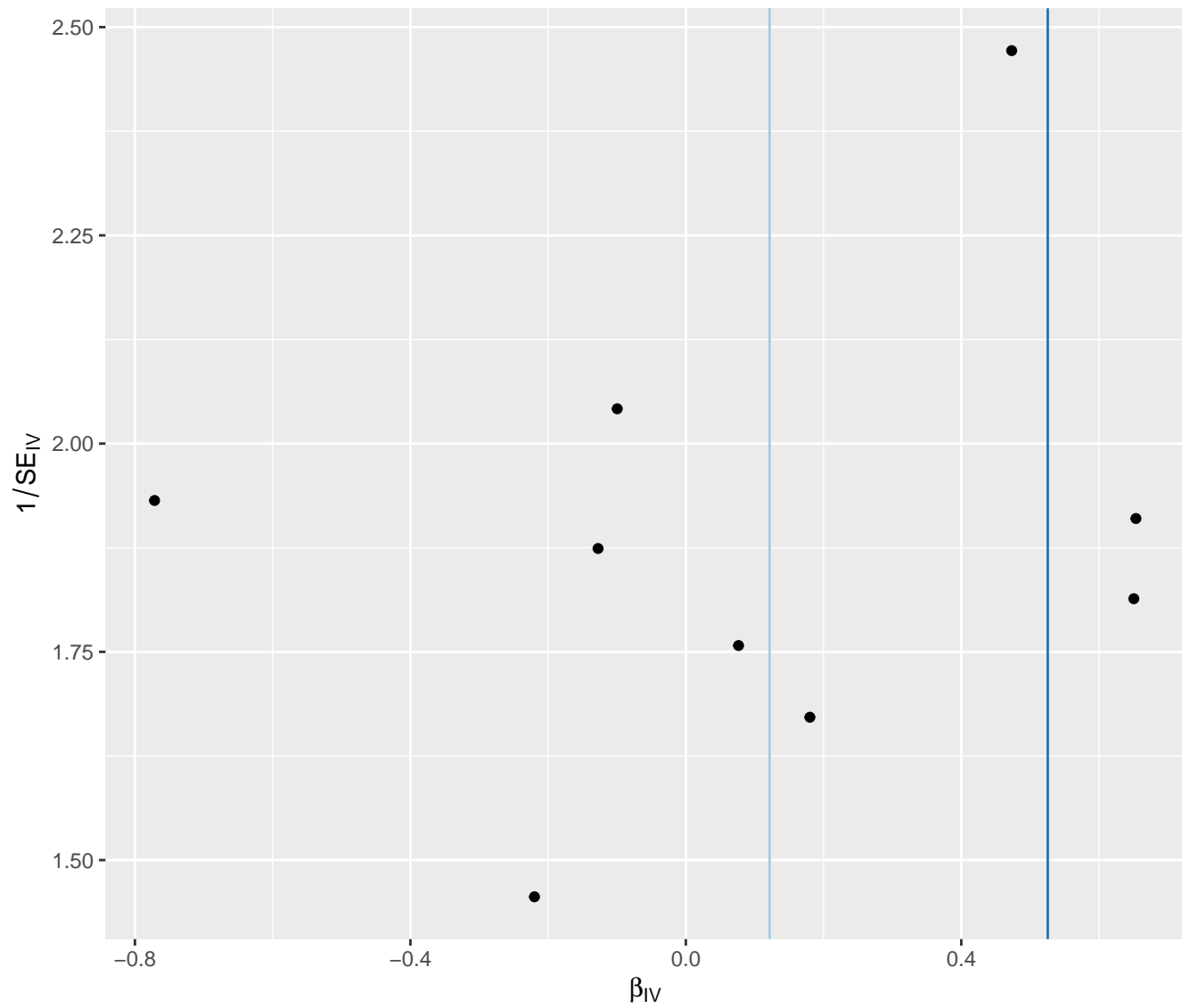

Supplement: Supplementary file 1 [file DataSheet1.zip › mendelian test/ebi-a-GCST90027457.csv_funnel.pdf]

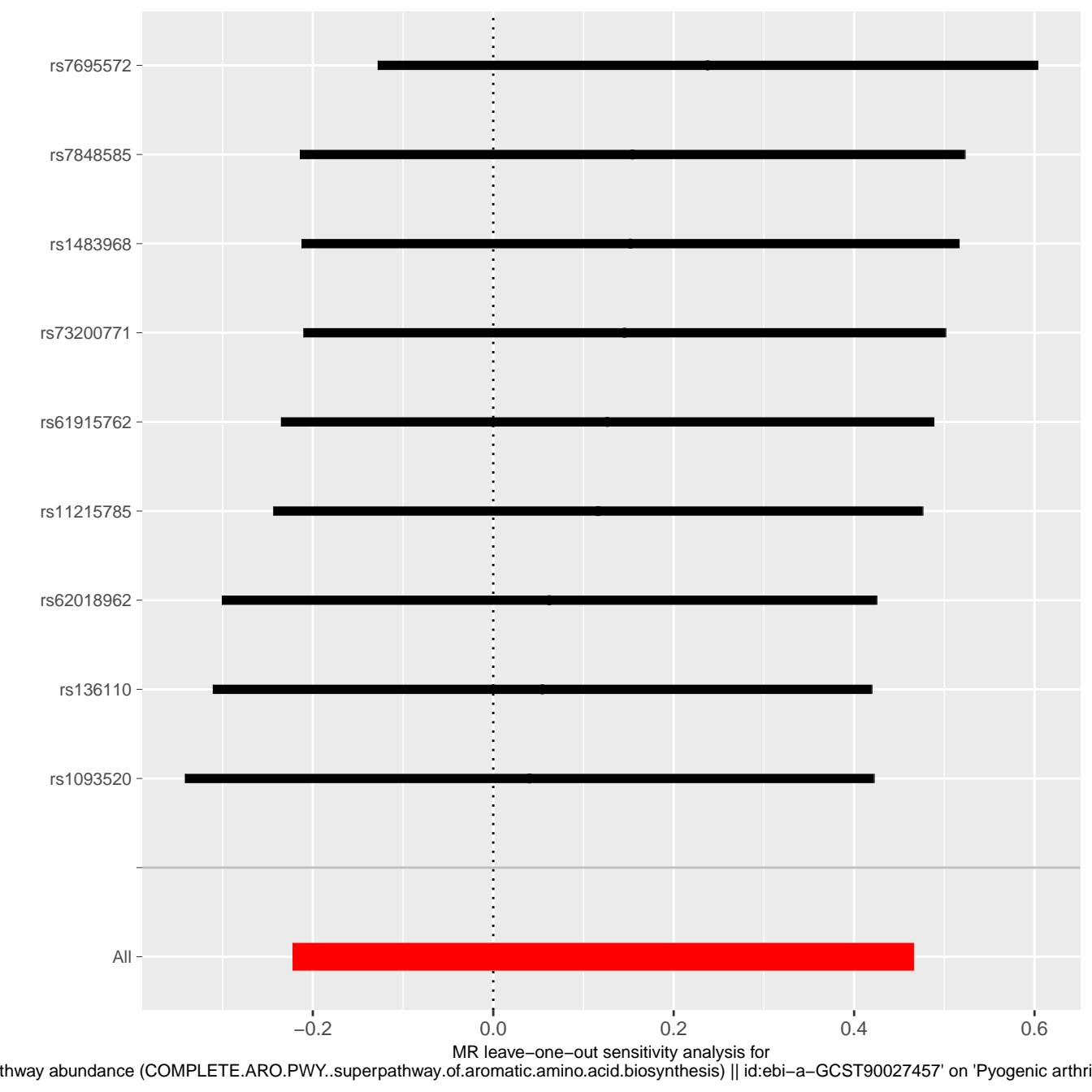

Supplement: Supplementary file 1 [file DataSheet1.zip › mendelian test/ebi-a-GCST90027457.csv_leave_one_out.pdf]

# MR Test

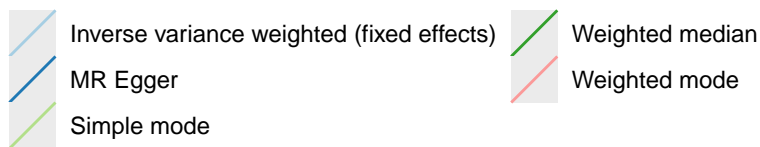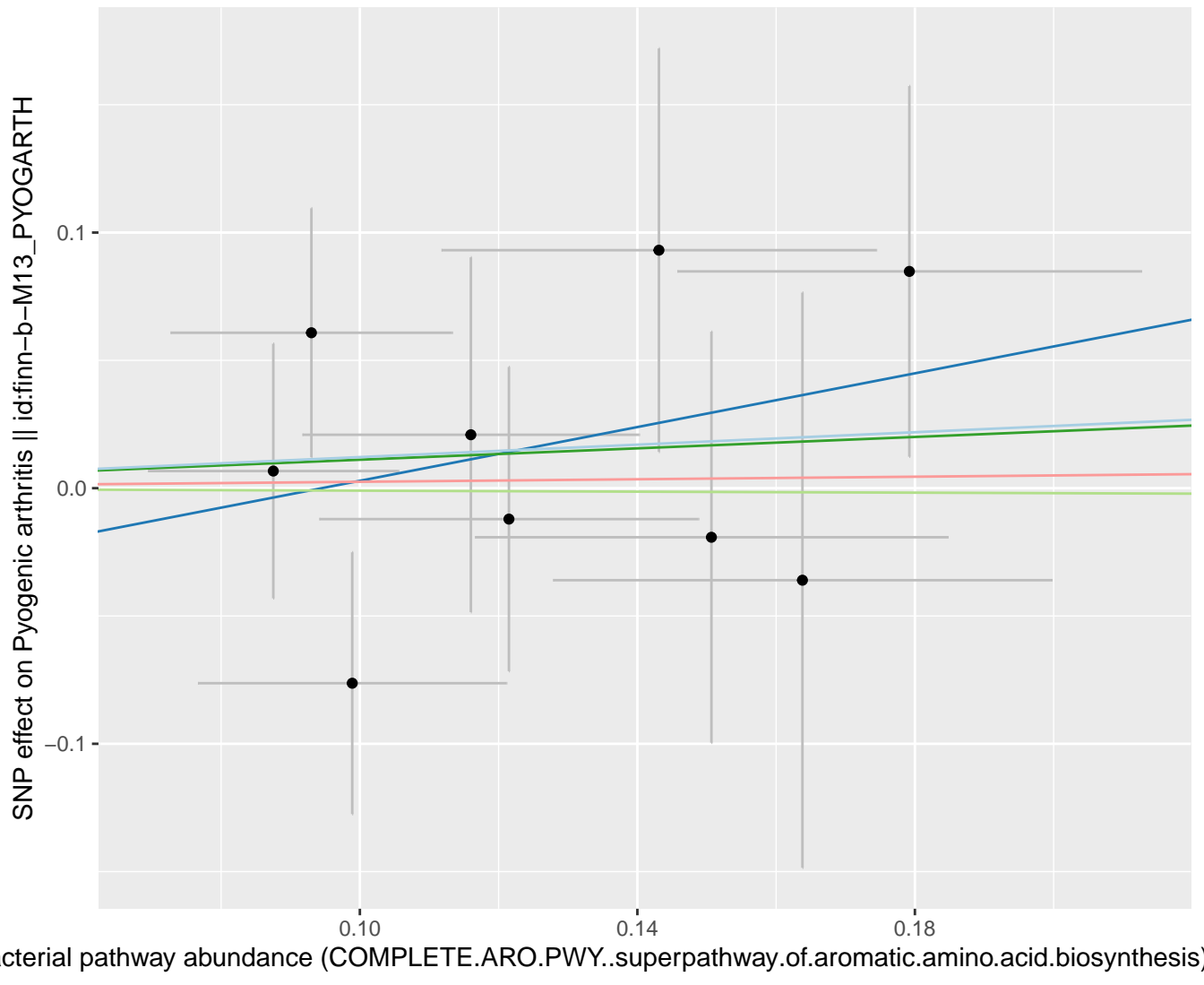

Supplement: Supplementary file 1 [file DataSheet1.zip › mendelian test/ebi-a-GCST90027457.csv_scatter.pdf]

# MR Method

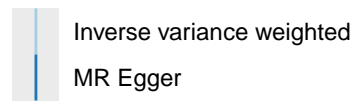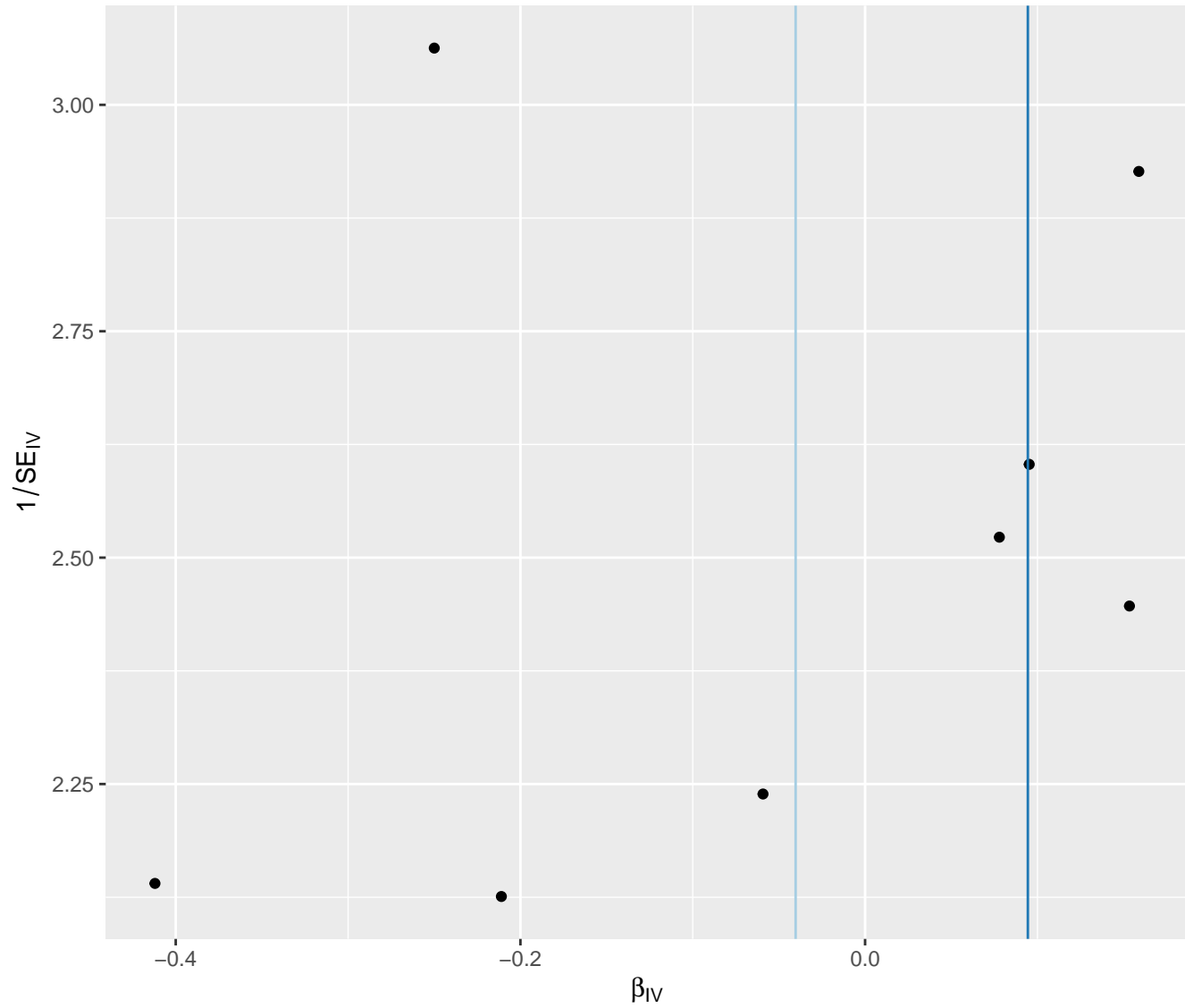

Supplement: Supplementary file 1 [file DataSheet1.zip › mendelian test/ebi-a-GCST90027458.csv_funnel.pdf]

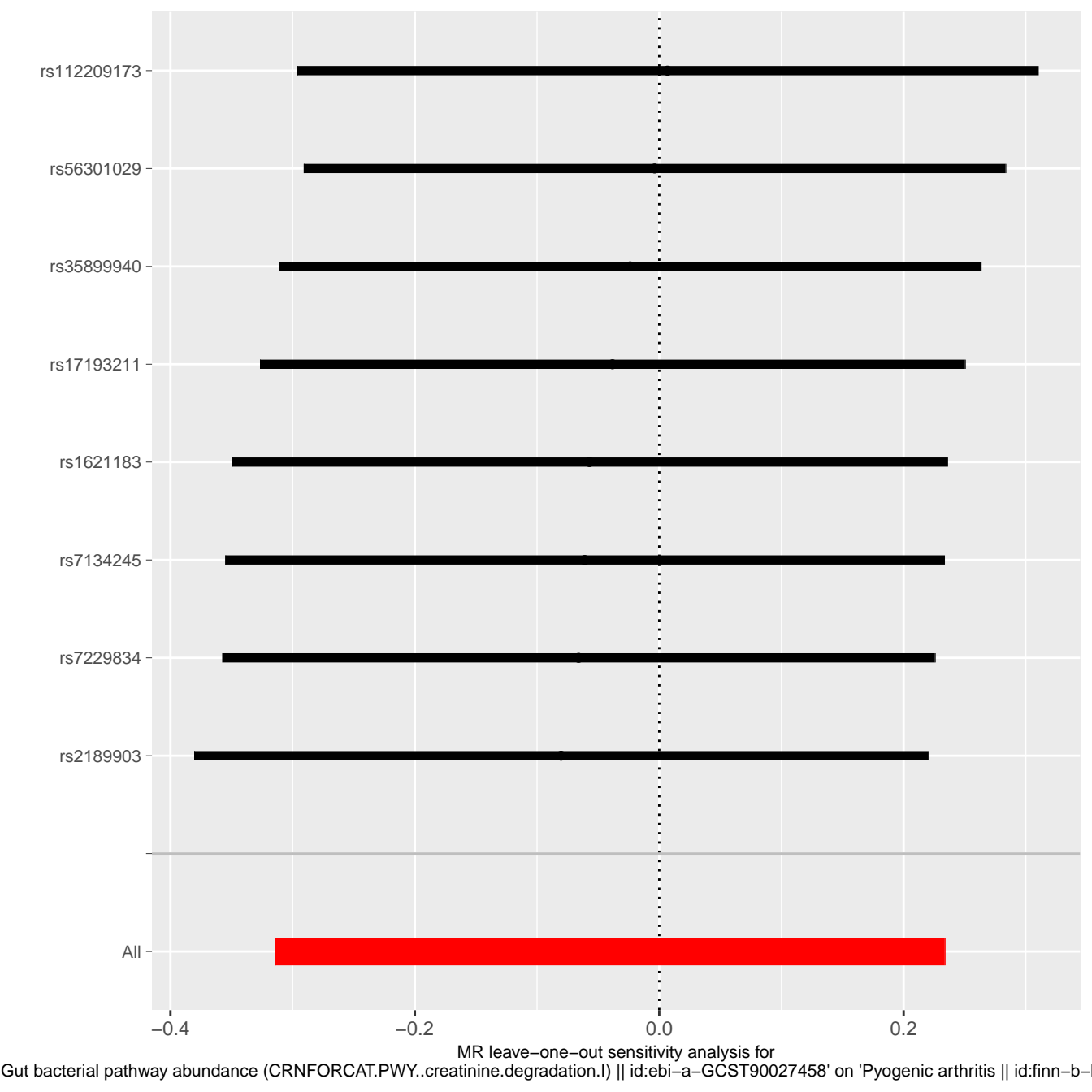

Supplement: Supplementary file 1 [file DataSheet1.zip › mendelian test/ebi-a-GCST90027458.csv_leave_one_out.pdf]

# MR Method

- Inverse variance weighted
- MR Egger

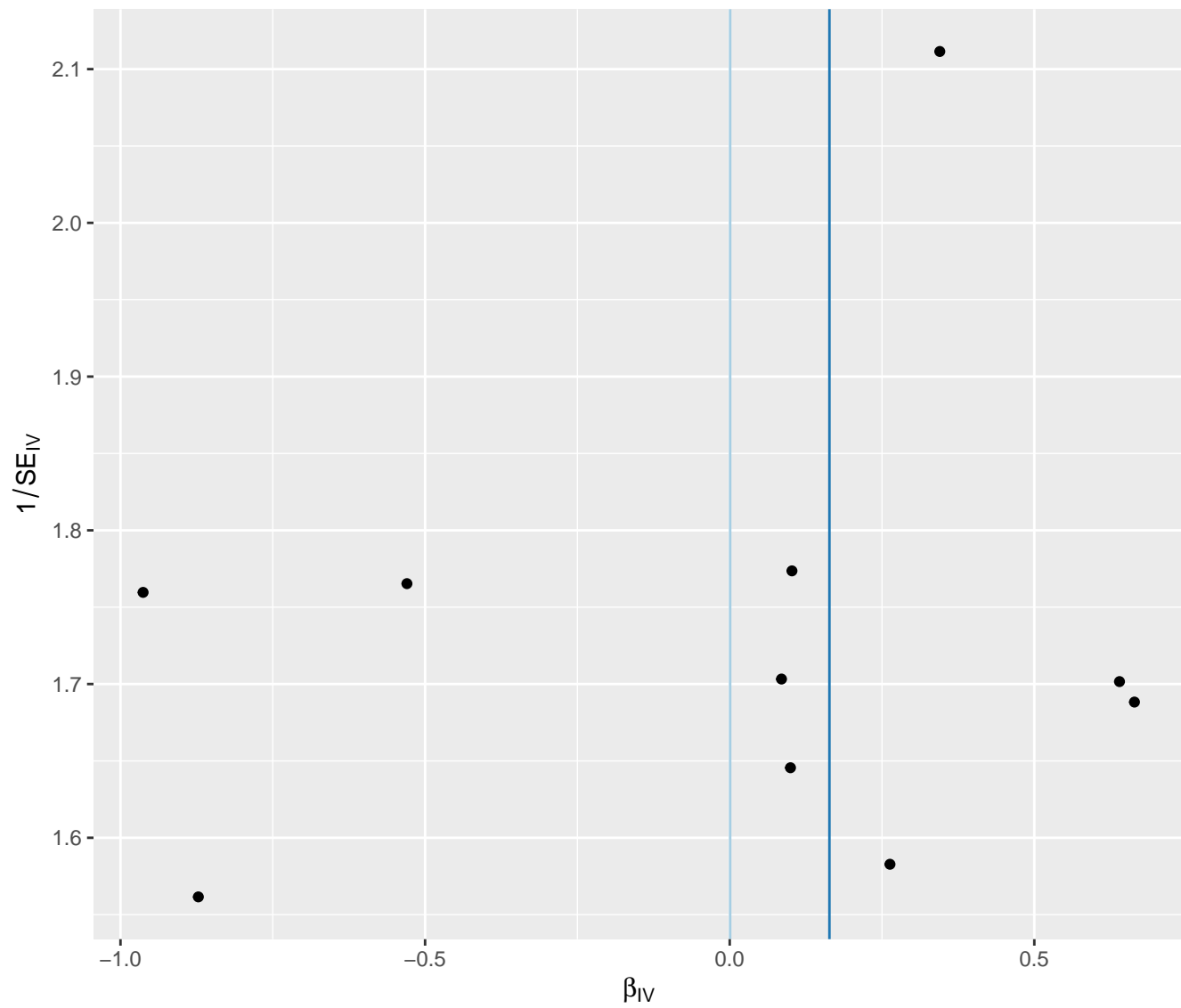

Supplement: Supplementary file 1 [file DataSheet1.zip › mendelian test/ebi-a-GCST90027459.csv_funnel.pdf]

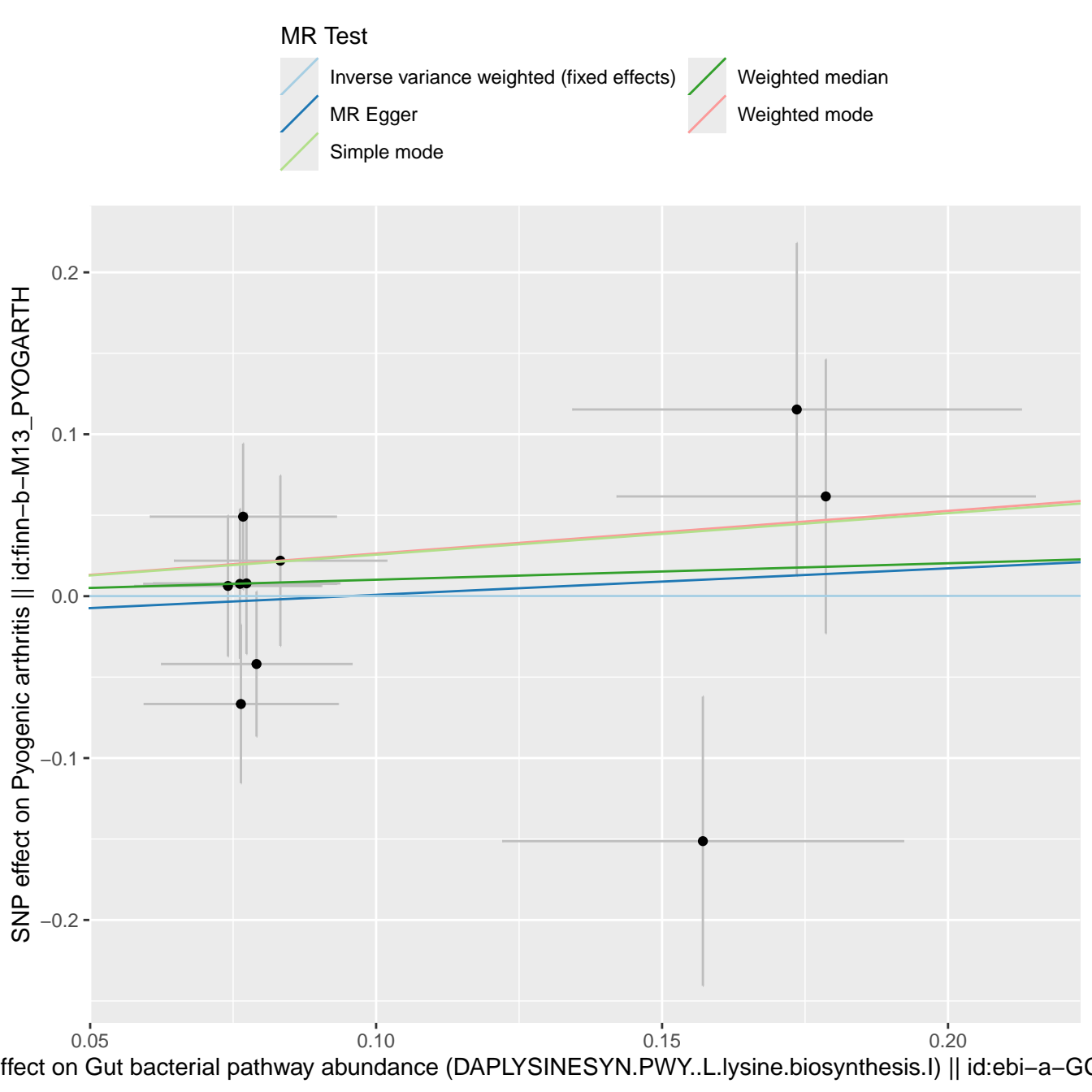

Supplement: Supplementary file 1 [file DataSheet1.zip › mendelian test/ebi-a-GCST90027459.csv_scatter.pdf]

# MR Method

- Inverse variance weighted
- MR Egger

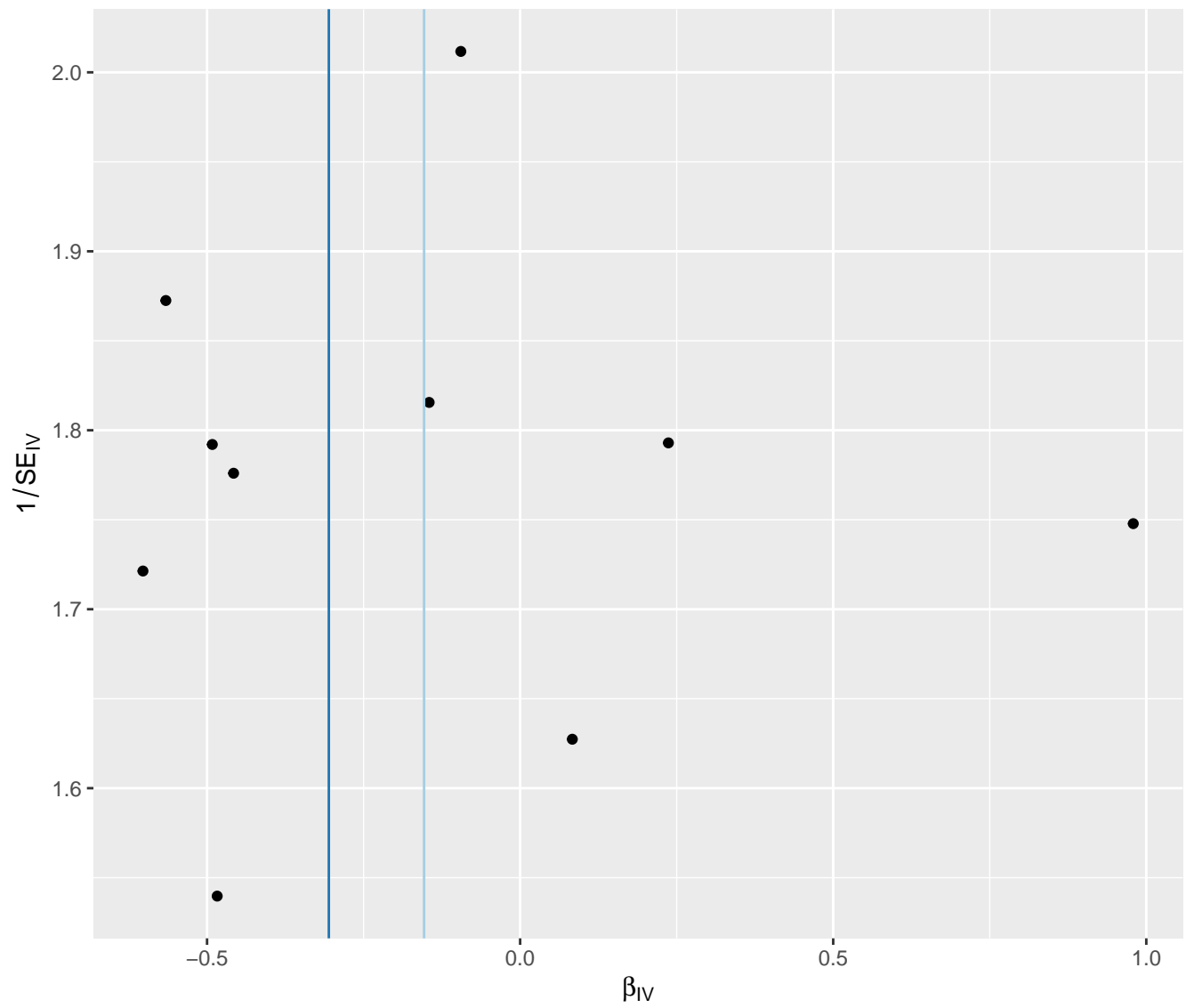

Supplement: Supplementary file 1 [file DataSheet1.zip › mendelian test/ebi-a-GCST90027460.csv_funnel.pdf]

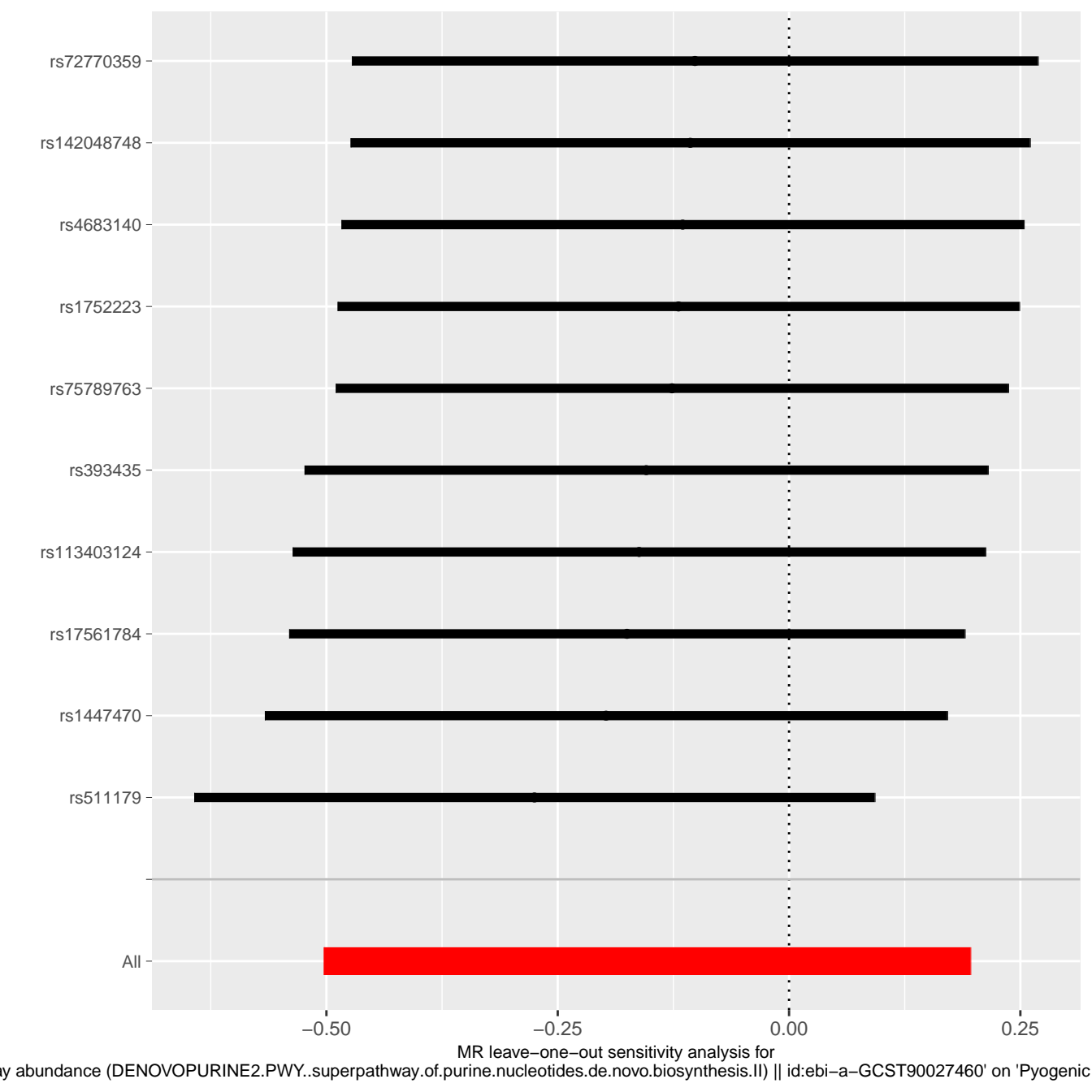

Supplement: Supplementary file 1 [file DataSheet1.zip › mendelian test/ebi-a-GCST90027460.csv_leave_one_out.pdf]

# MR Test

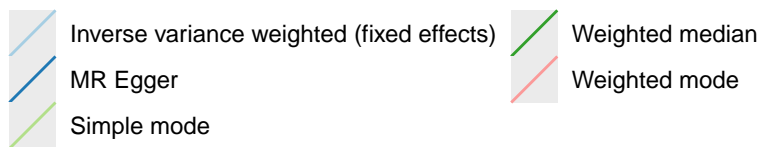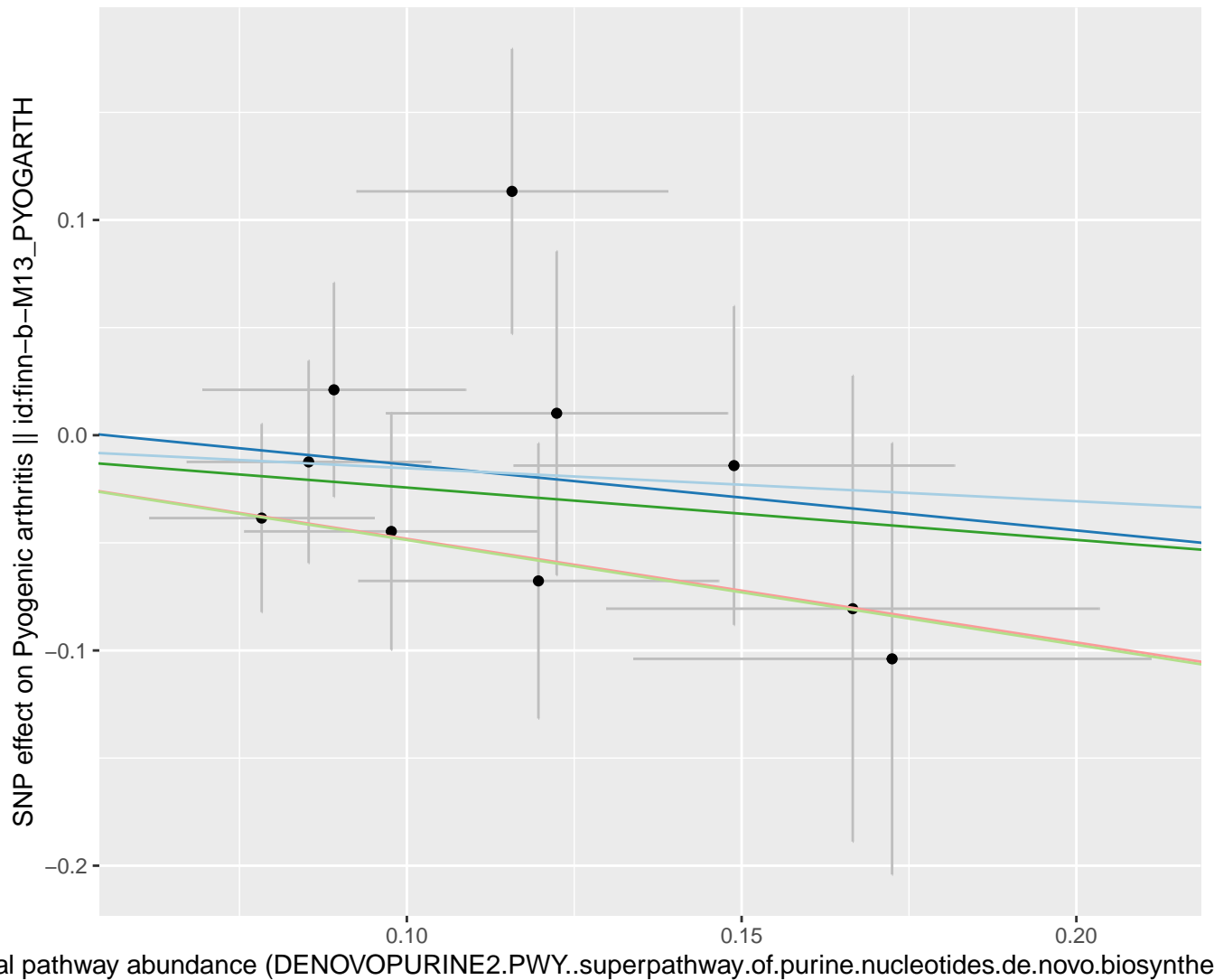

Supplement: Supplementary file 1 [file DataSheet1.zip › mendelian test/ebi-a-GCST90027460.csv_scatter.pdf]

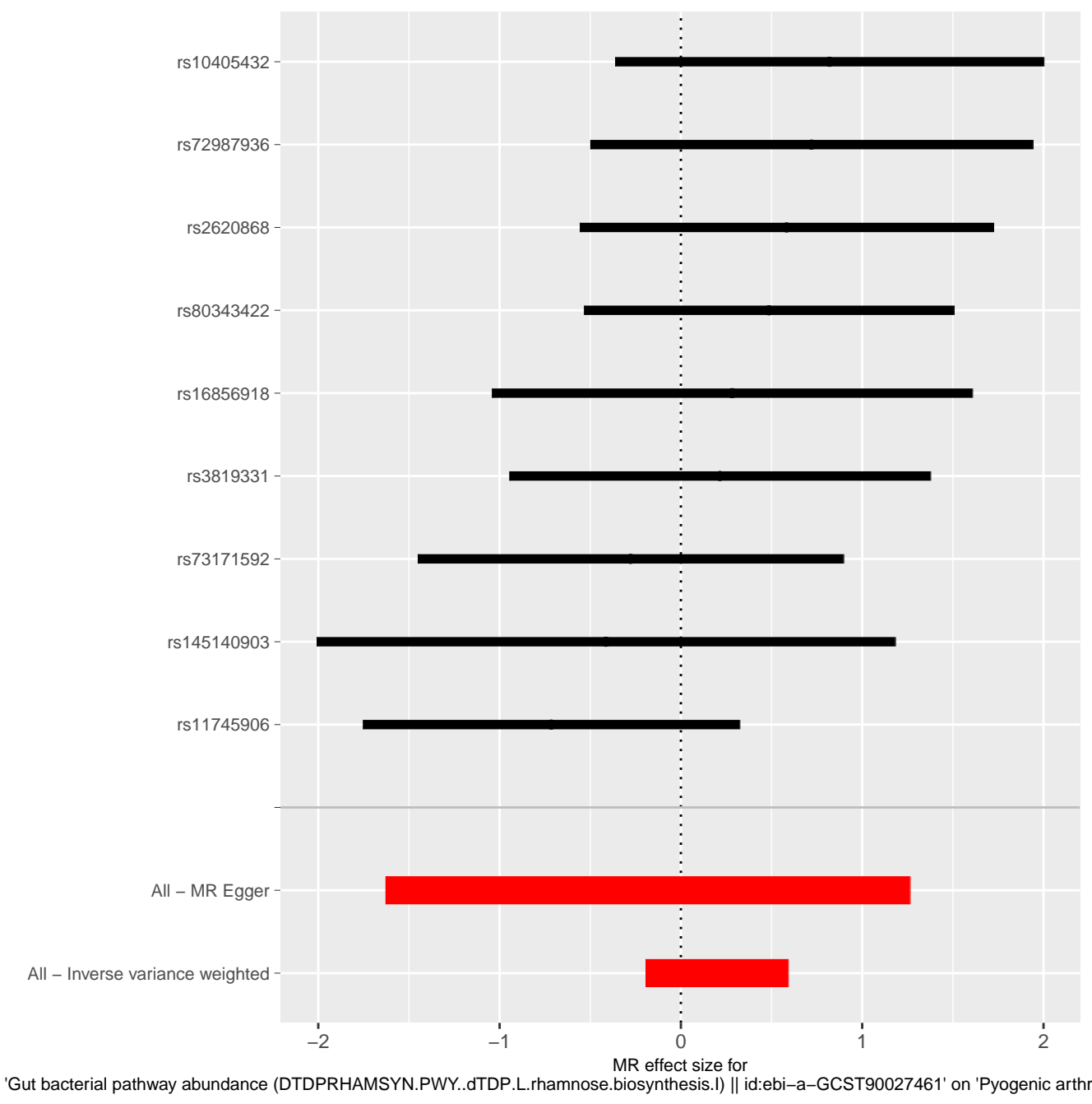

Supplement: Supplementary file 1 [file DataSheet1.zip › mendelian test/ebi-a-GCST90027461.csv_forest.pdf]

# MR Method

- Inverse variance weighted
- MR Egger

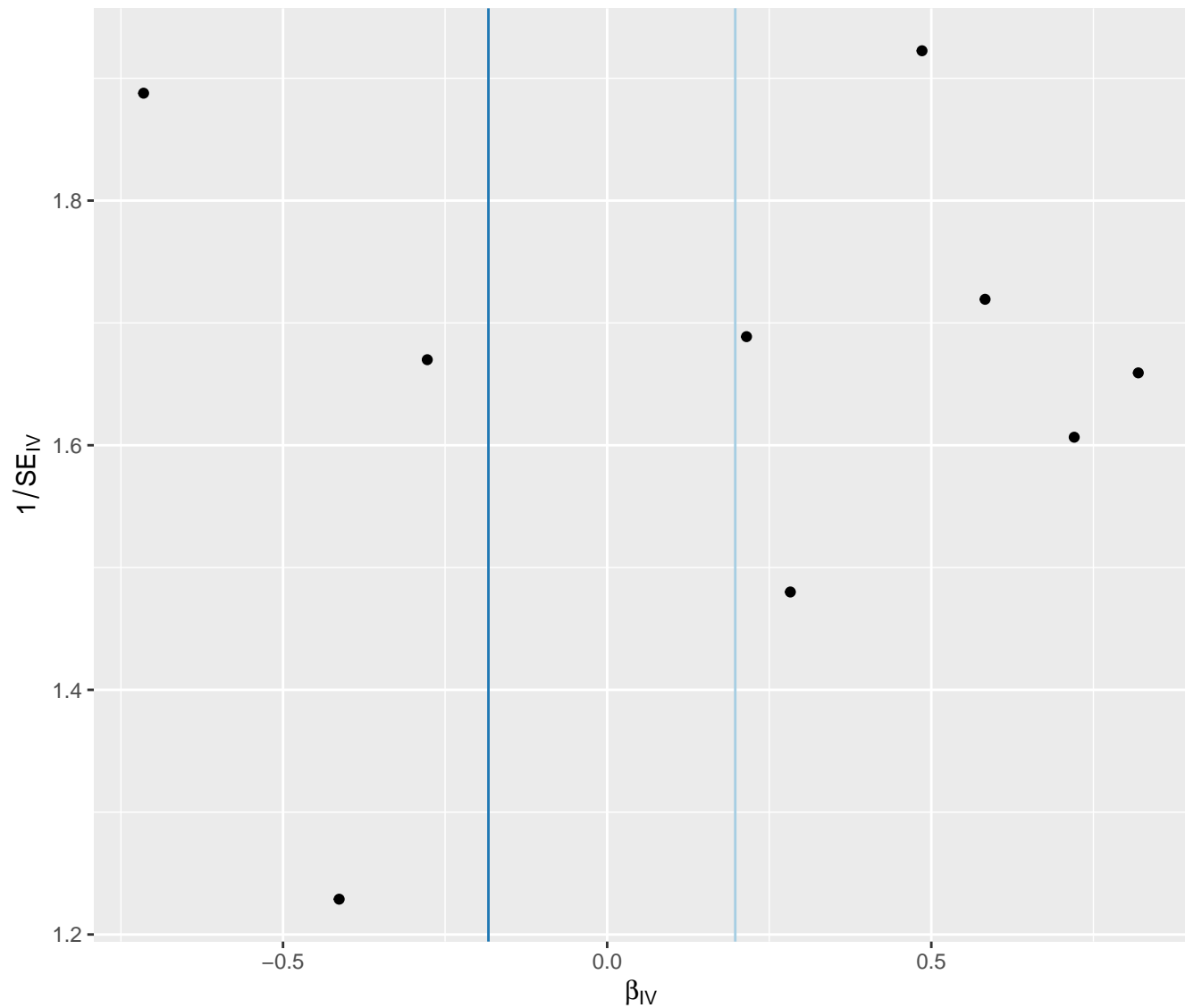

Supplement: Supplementary file 1 [file DataSheet1.zip › mendelian test/ebi-a-GCST90027461.csv_funnel.pdf]

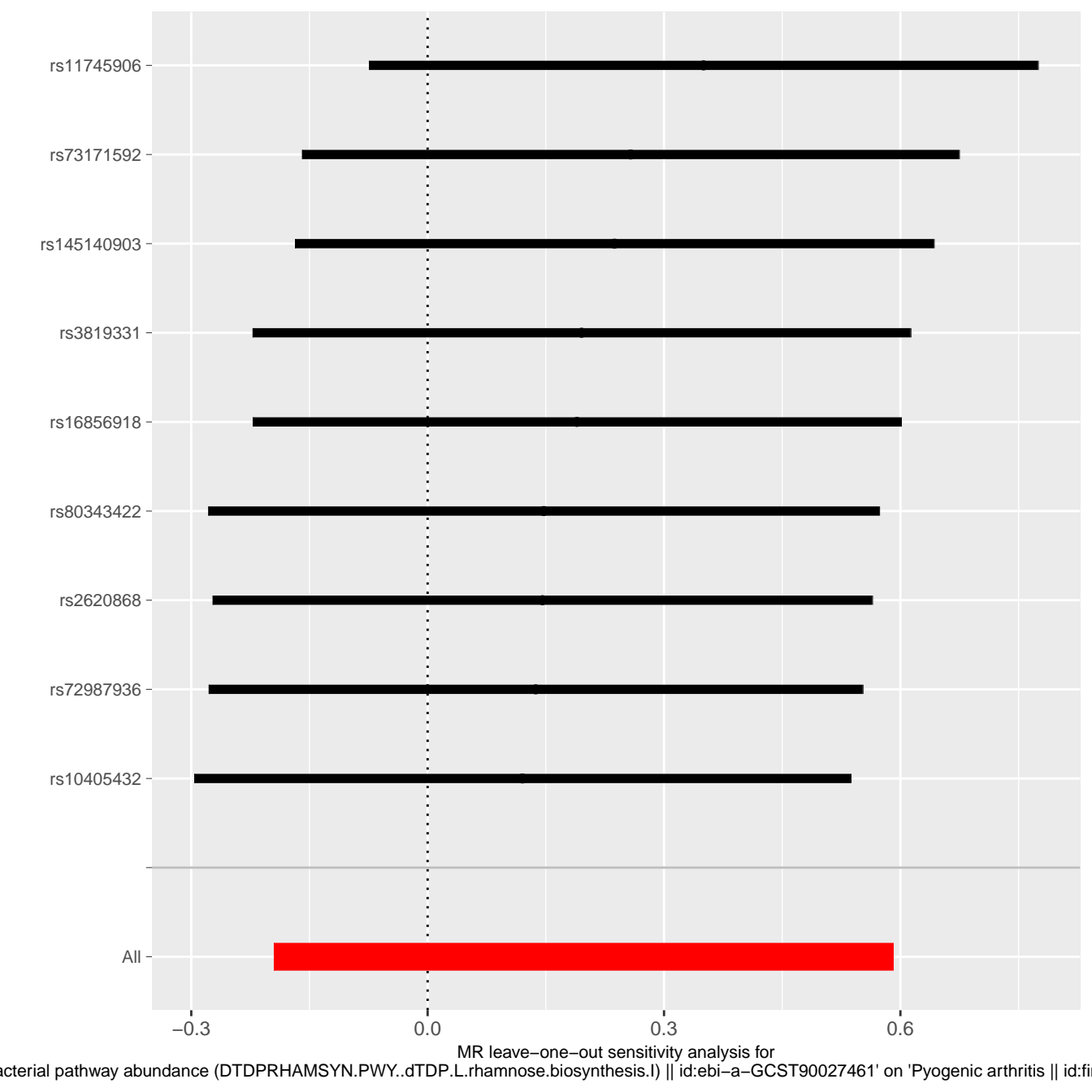

Supplement: Supplementary file 1 [file DataSheet1.zip › mendelian test/ebi-a-GCST90027461.csv_leave_one_out.pdf]

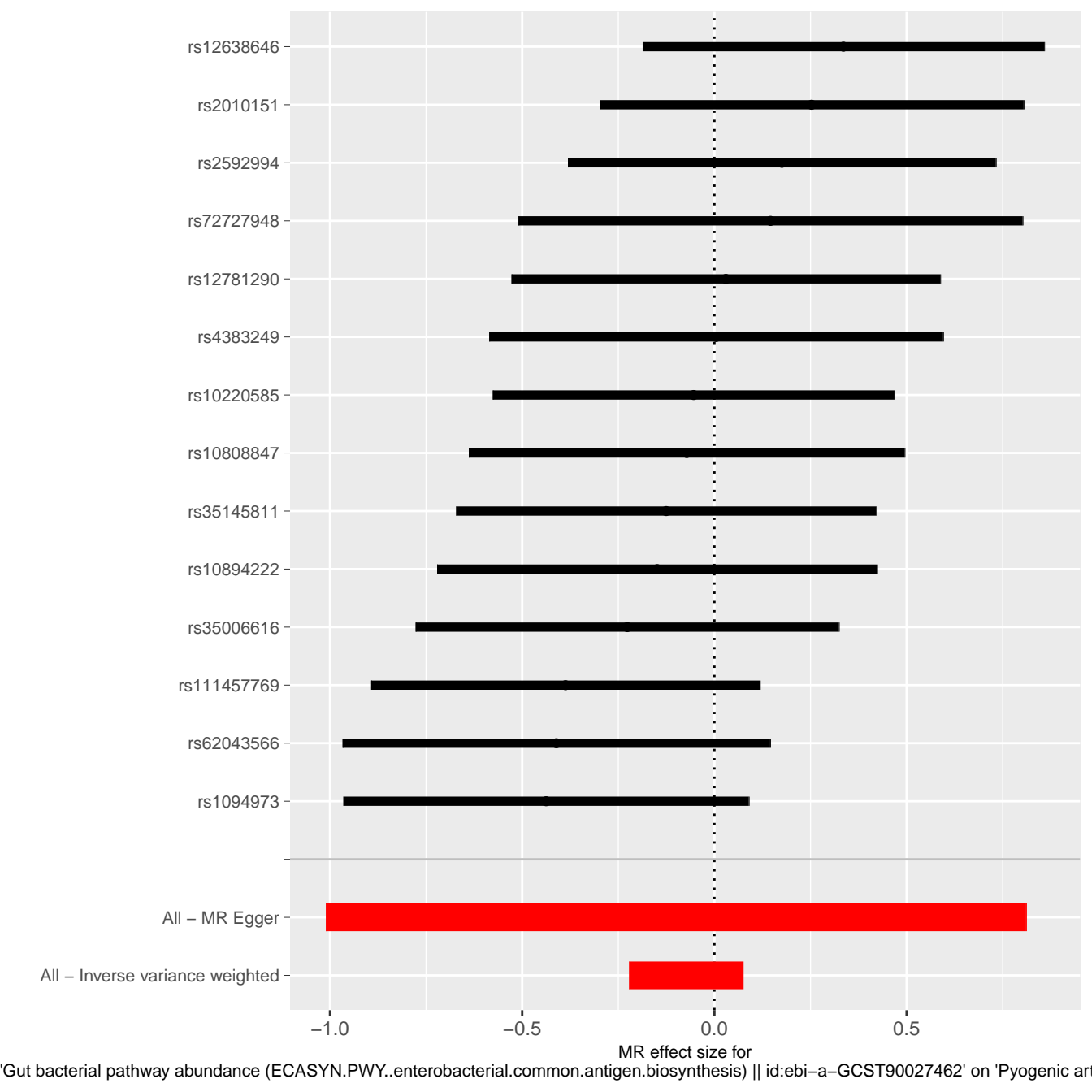

Supplement: Supplementary file 1 [file DataSheet1.zip › mendelian test/ebi-a-GCST90027462.csv_forest.pdf]

# MR Method

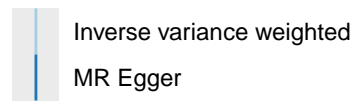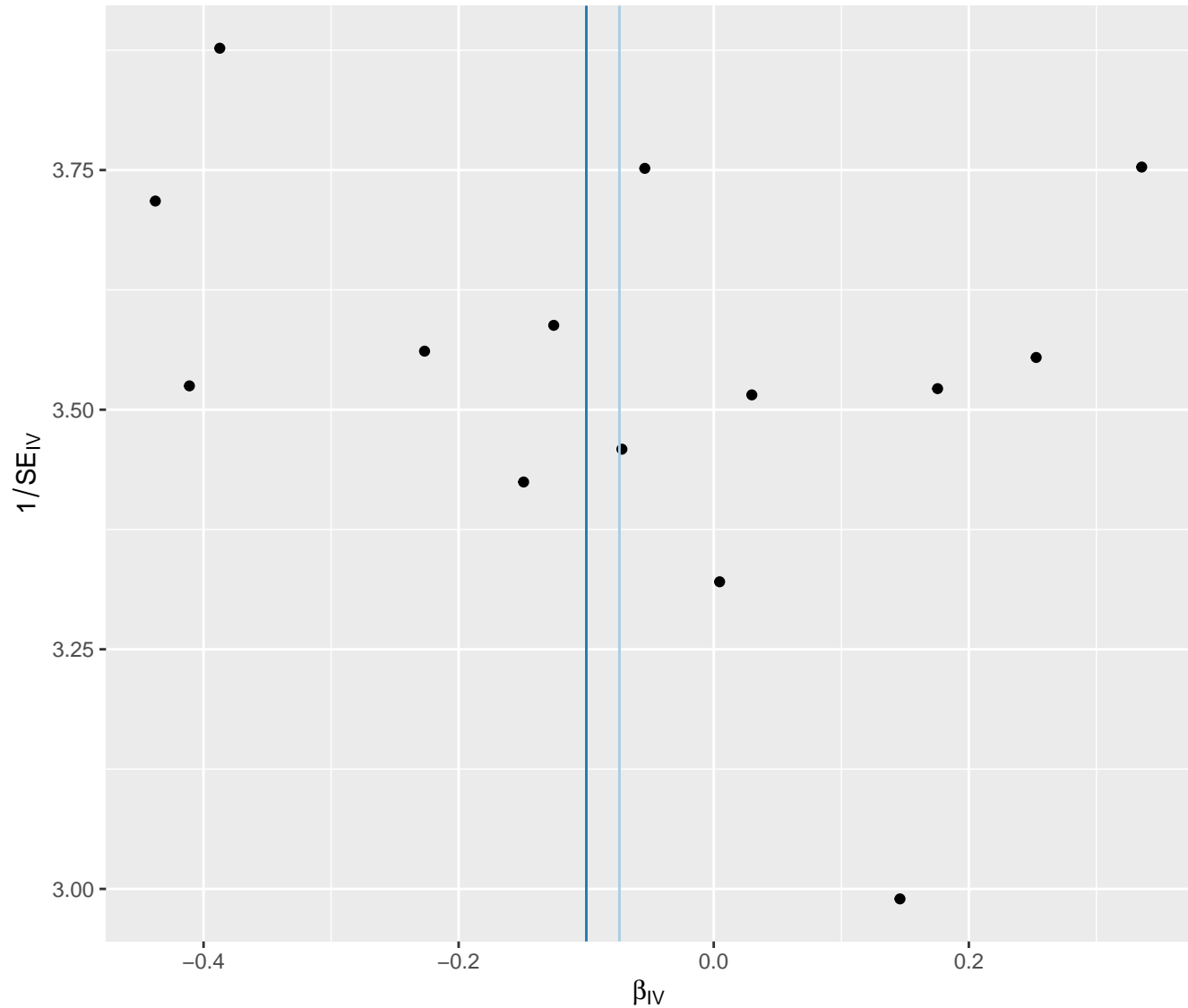

Supplement: Supplementary file 1 [file DataSheet1.zip › mendelian test/ebi-a-GCST90027462.csv_funnel.pdf]

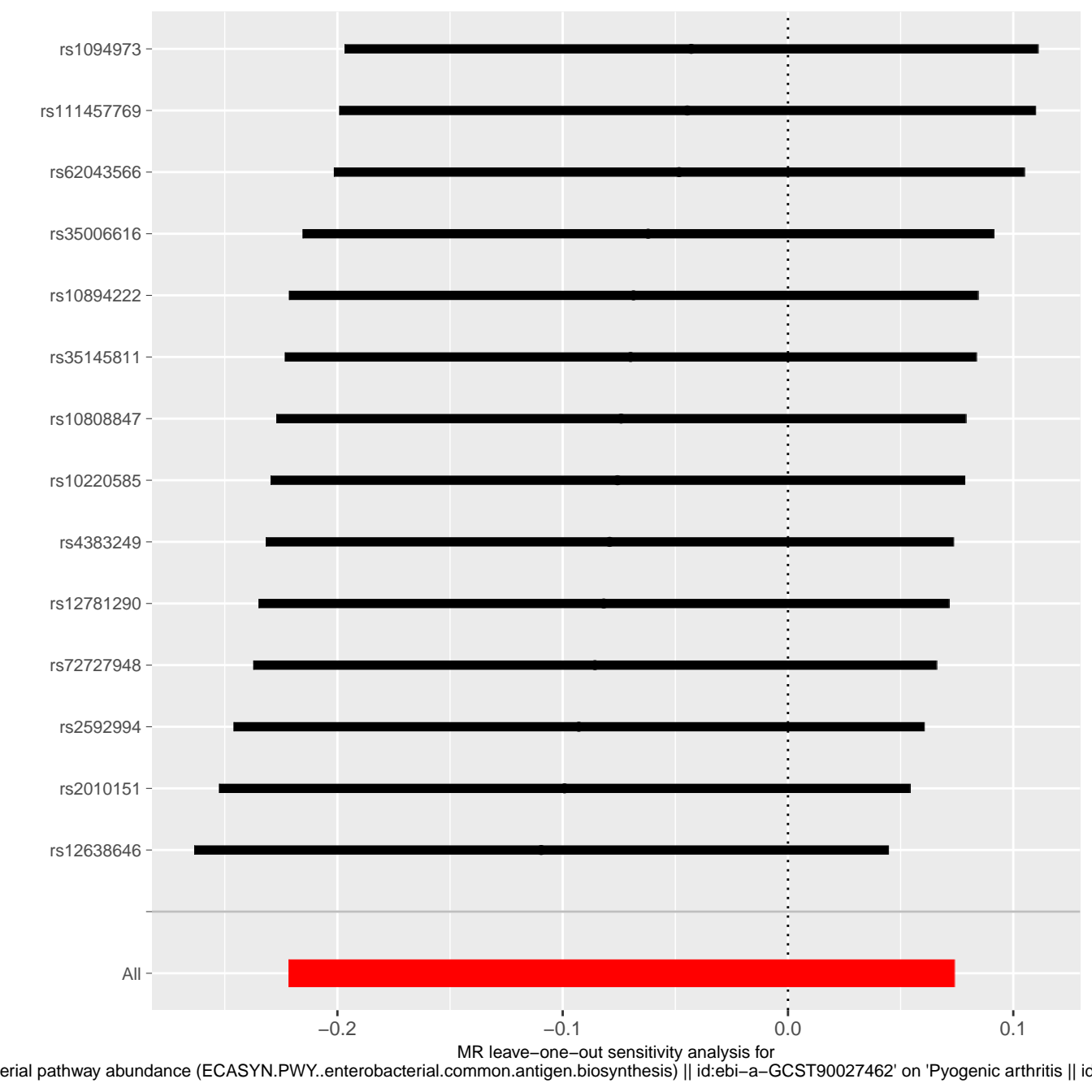

Supplement: Supplementary file 1 [file DataSheet1.zip › mendelian test/ebi-a-GCST90027462.csv_leave_one_out.pdf]

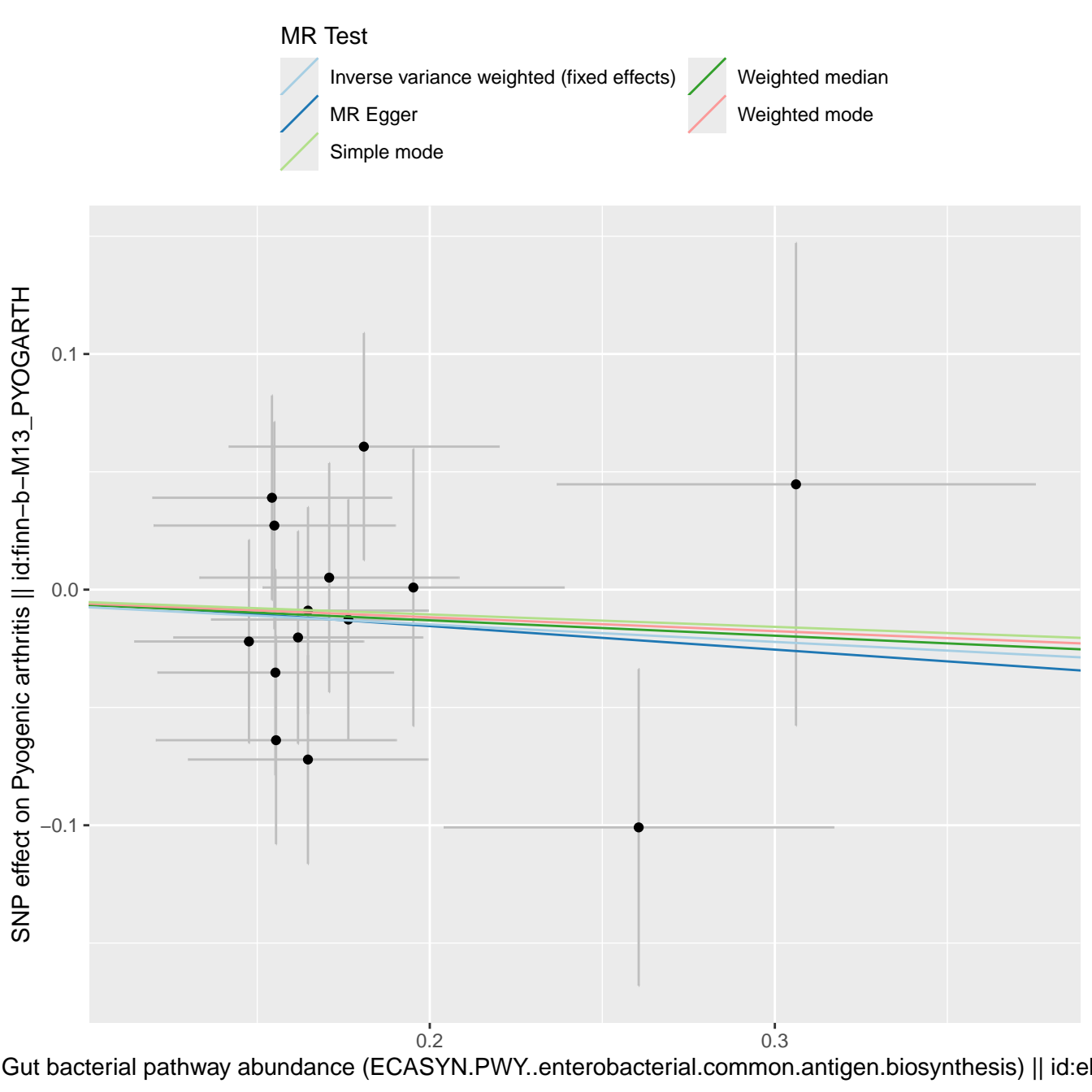

Supplement: Supplementary file 1 [file DataSheet1.zip › mendelian test/ebi-a-GCST90027462.csv_scatter.pdf]

# MR Method

- Inverse variance weighted
- MR Egger

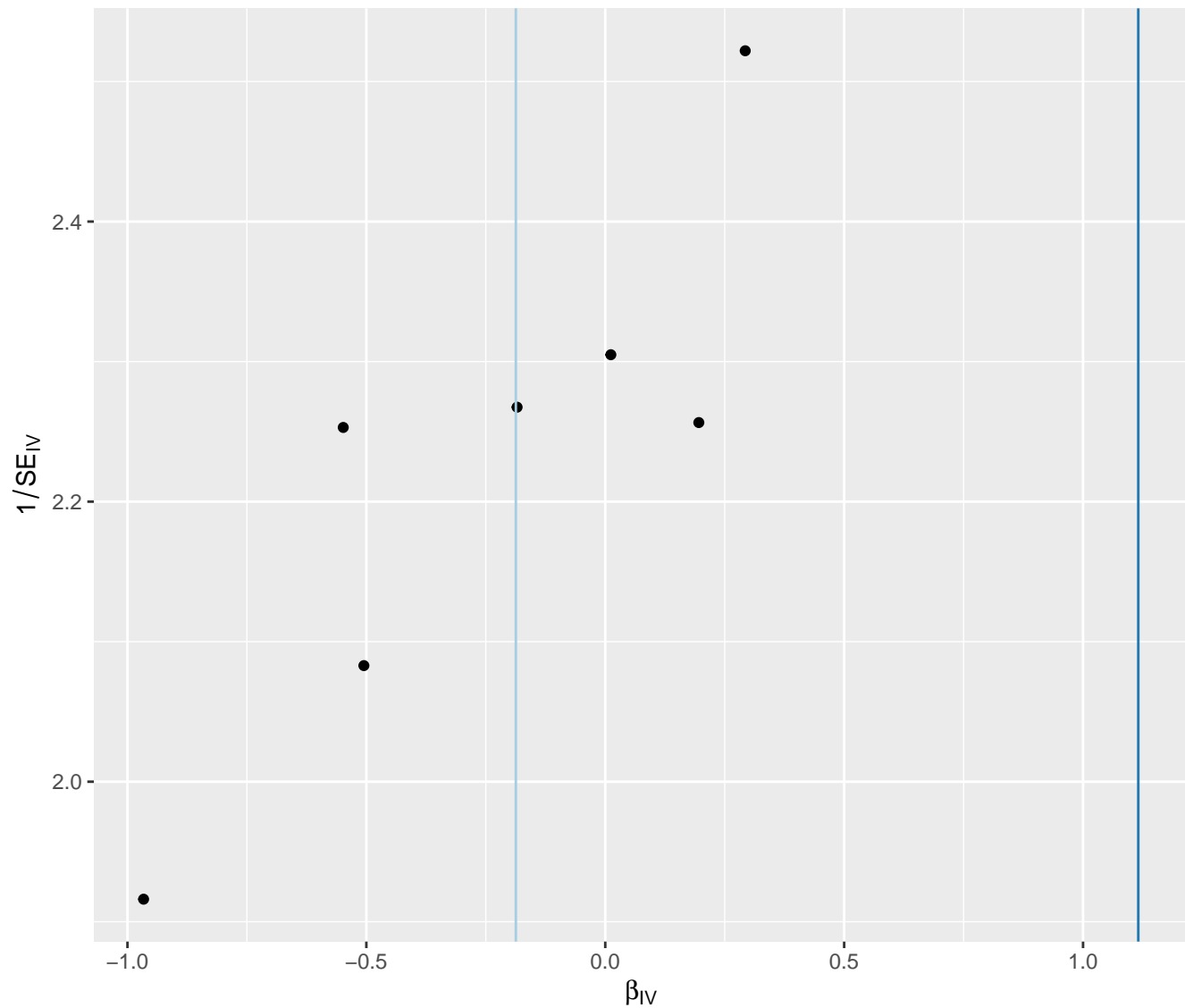

Supplement: Supplementary file 1 [file DataSheet1.zip › mendelian test/ebi-a-GCST90027464.csv_funnel.pdf]

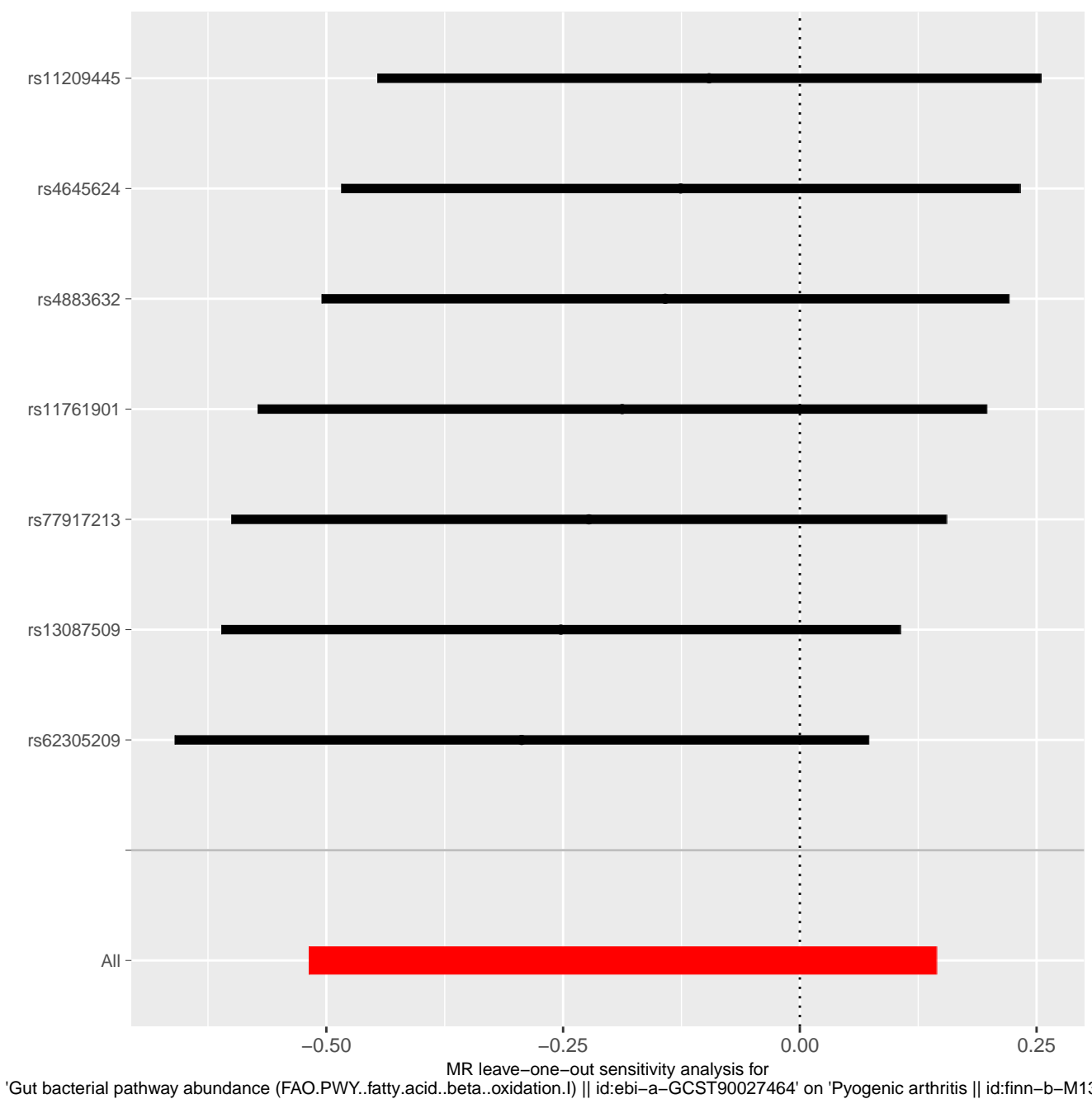

Supplement: Supplementary file 1 [file DataSheet1.zip › mendelian test/ebi-a-GCST90027464.csv_leave_one_out.pdf]

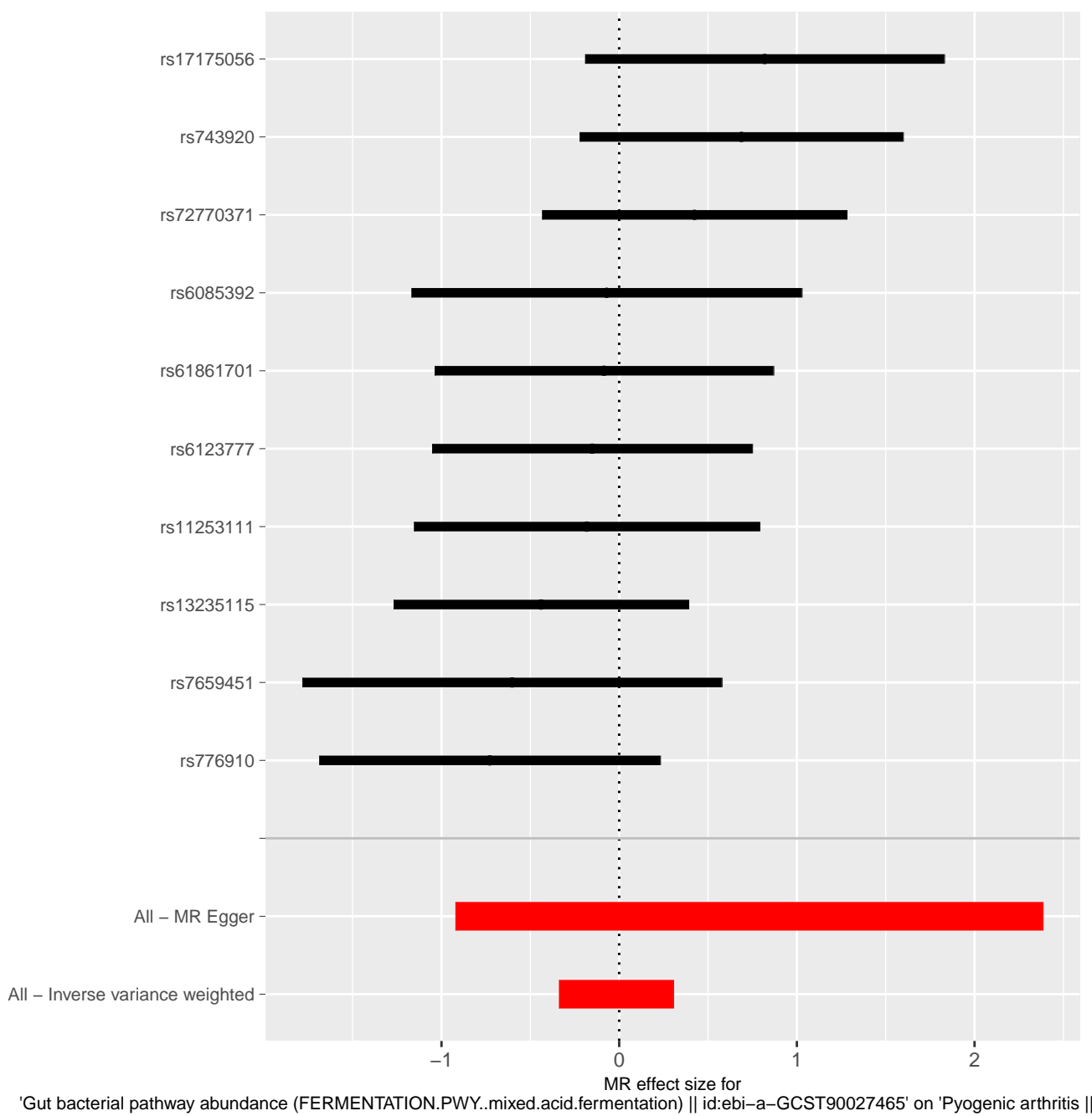

Supplement: Supplementary file 1 [file DataSheet1.zip › mendelian test/ebi-a-GCST90027465.csv_forest.pdf]

# MR Method

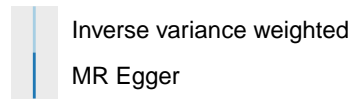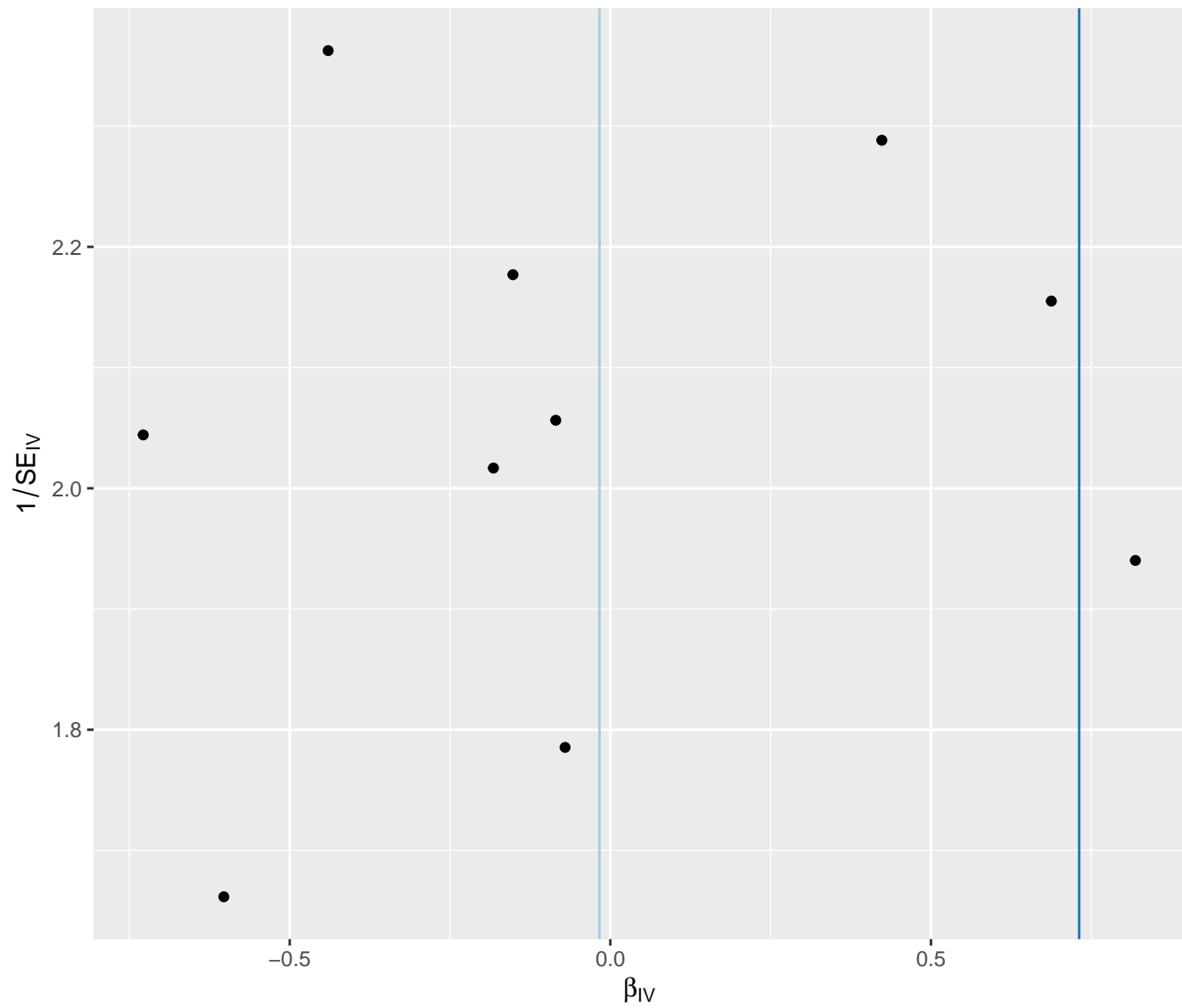

Supplement: Supplementary file 1 [file DataSheet1.zip › mendelian test/ebi-a-GCST90027465.csv_funnel.pdf]

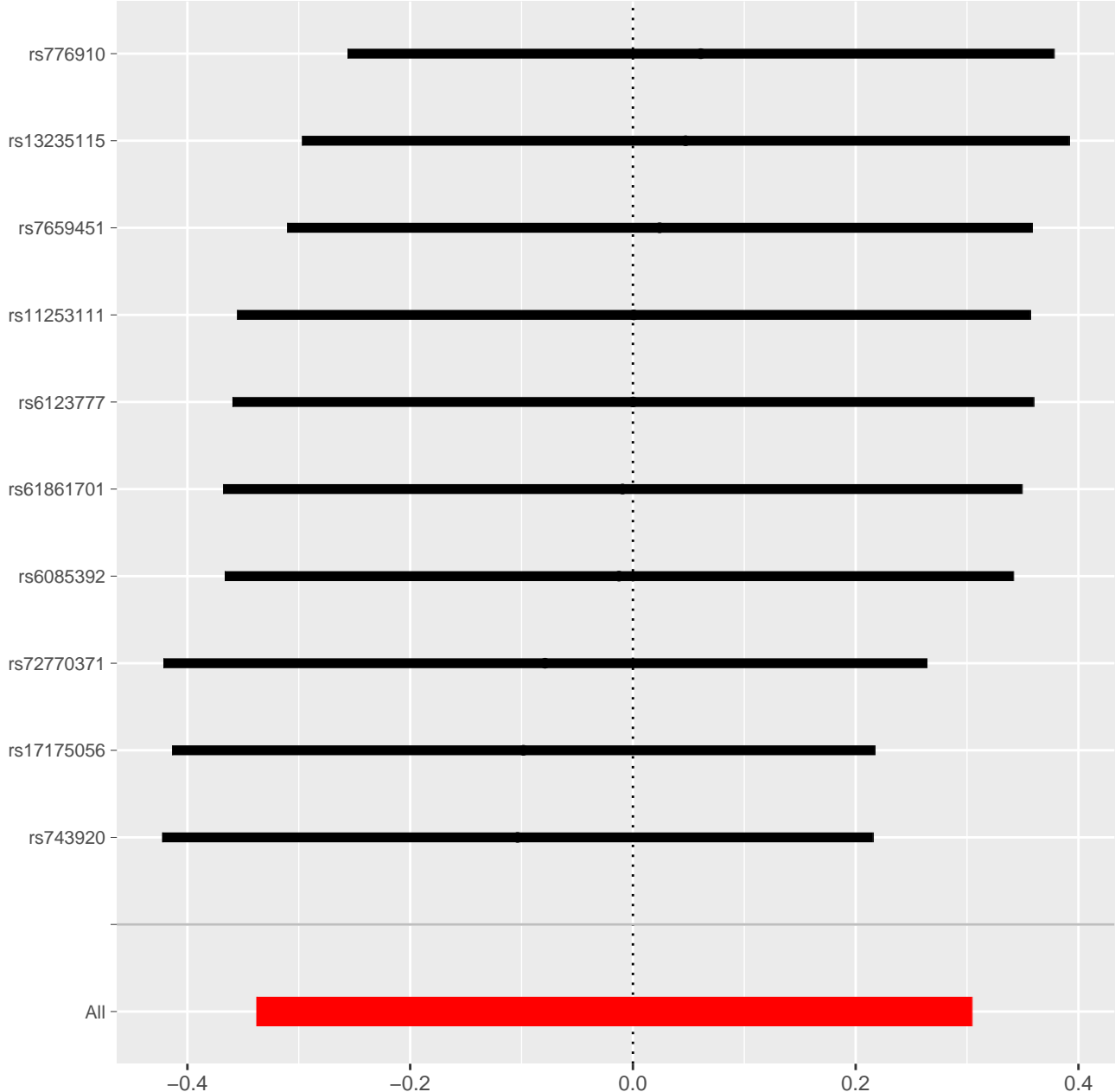

Supplement: Supplementary file 1 [file DataSheet1.zip › mendelian test/ebi-a-GCST90027465.csv_leave_one_out.pdf]

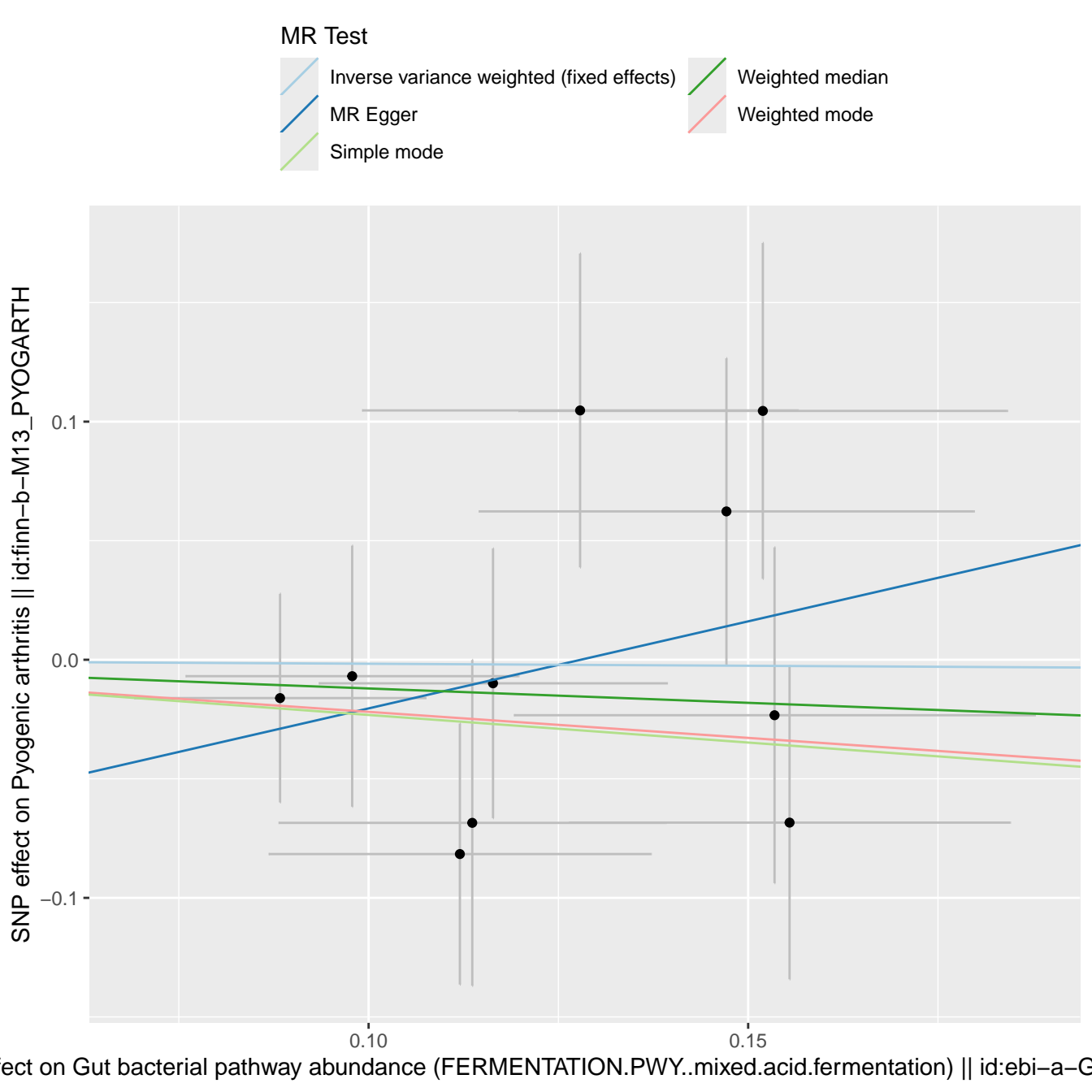

Supplement: Supplementary file 1 [file DataSheet1.zip › mendelian test/ebi-a-GCST90027465.csv_scatter.pdf]

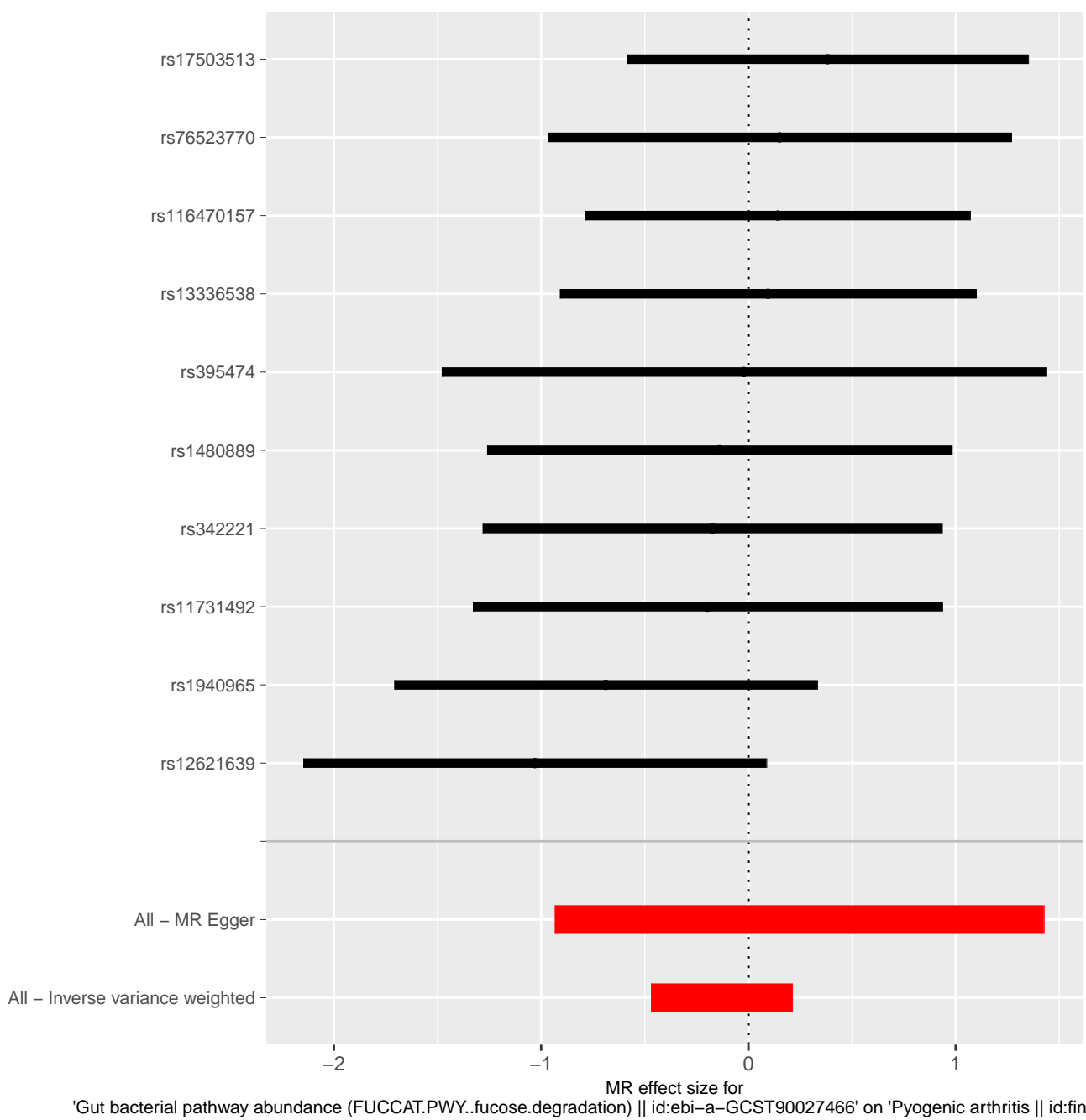

Supplement: Supplementary file 1 [file DataSheet1.zip › mendelian test/ebi-a-GCST90027466.csv_forest.pdf]

# MR Method

- Inverse variance weighted
- MR Egger

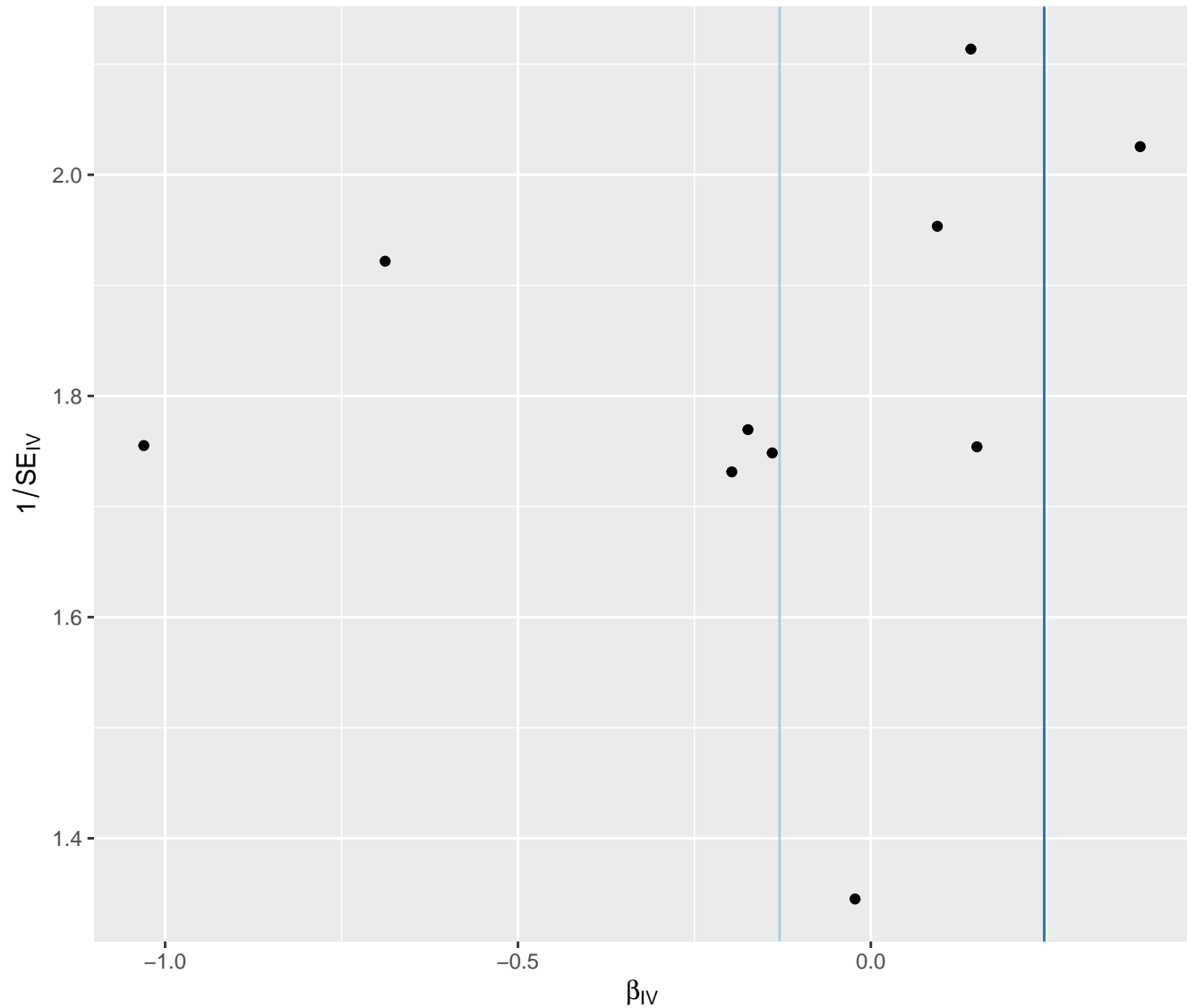

Supplement: Supplementary file 1 [file DataSheet1.zip › mendelian test/ebi-a-GCST90027466.csv_funnel.pdf]

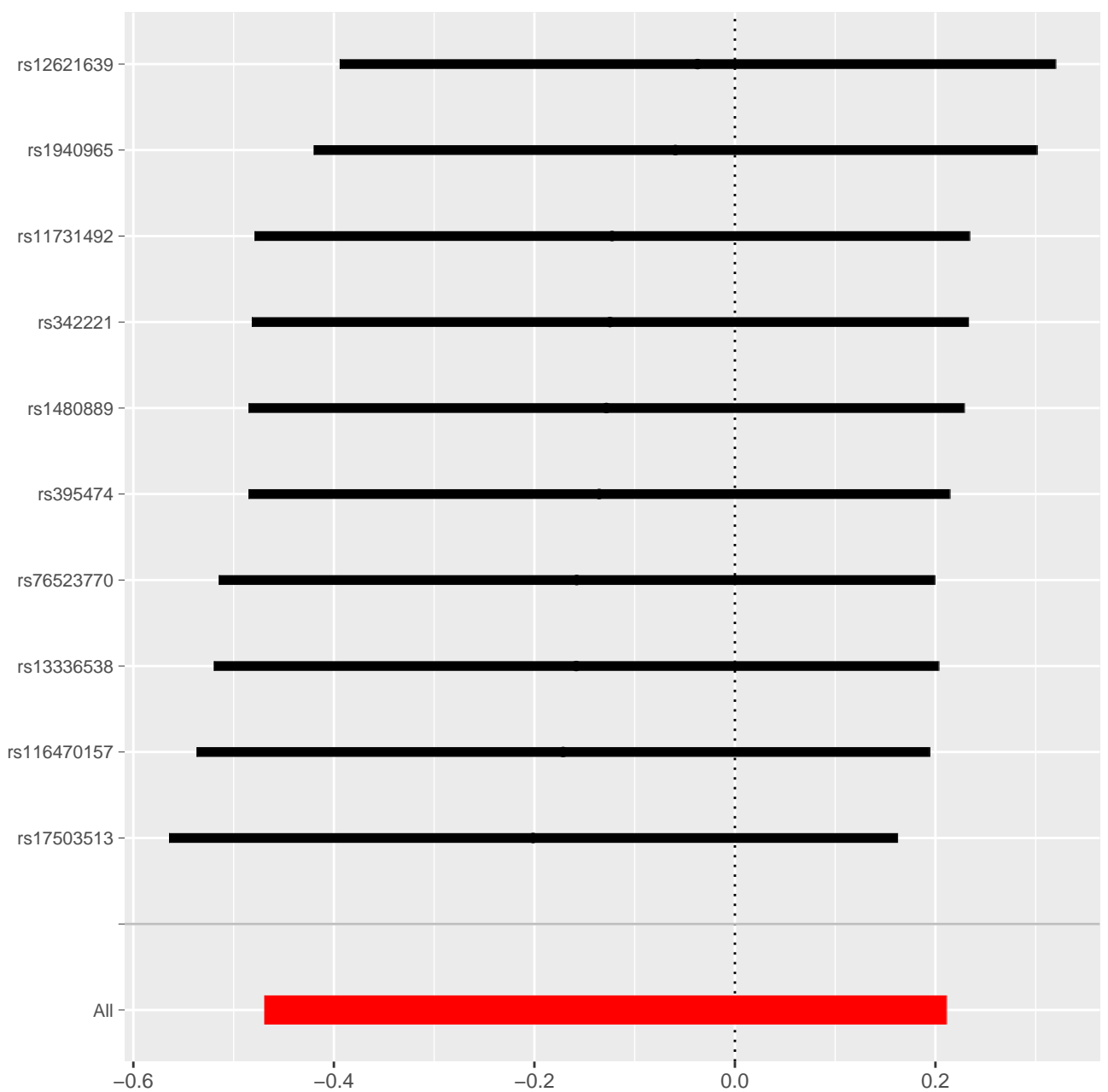

Supplement: Supplementary file 1 [file DataSheet1.zip › mendelian test/ebi-a-GCST90027466.csv_leave_one_out.pdf]

# MR Test

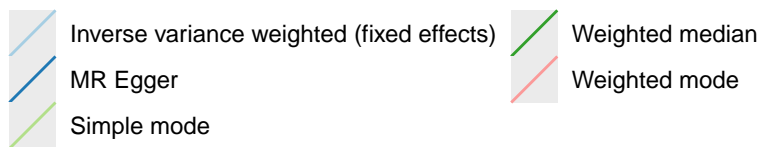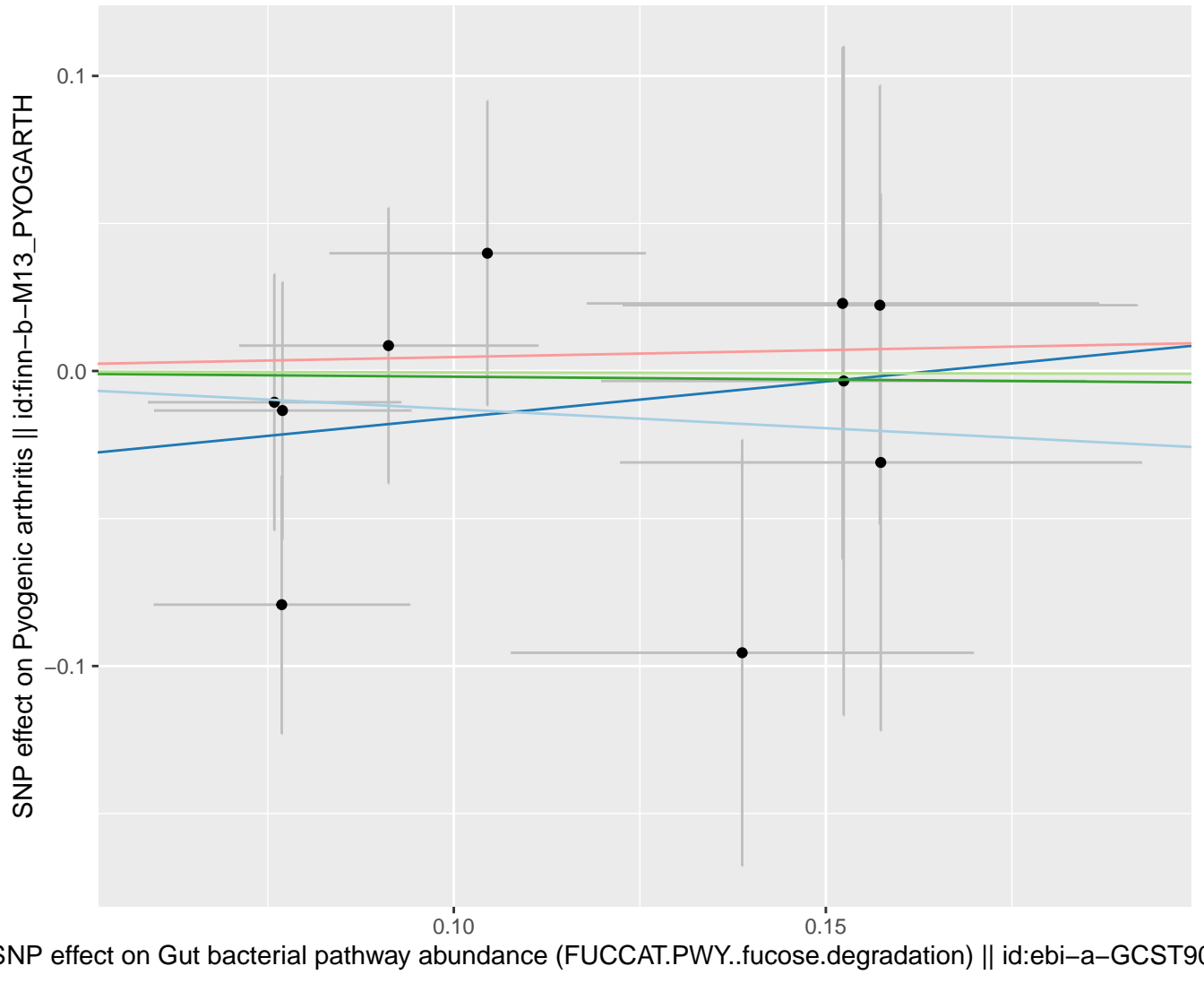

Supplement: Supplementary file 1 [file DataSheet1.zip › mendelian test/ebi-a-GCST90027466.csv_scatter.pdf]

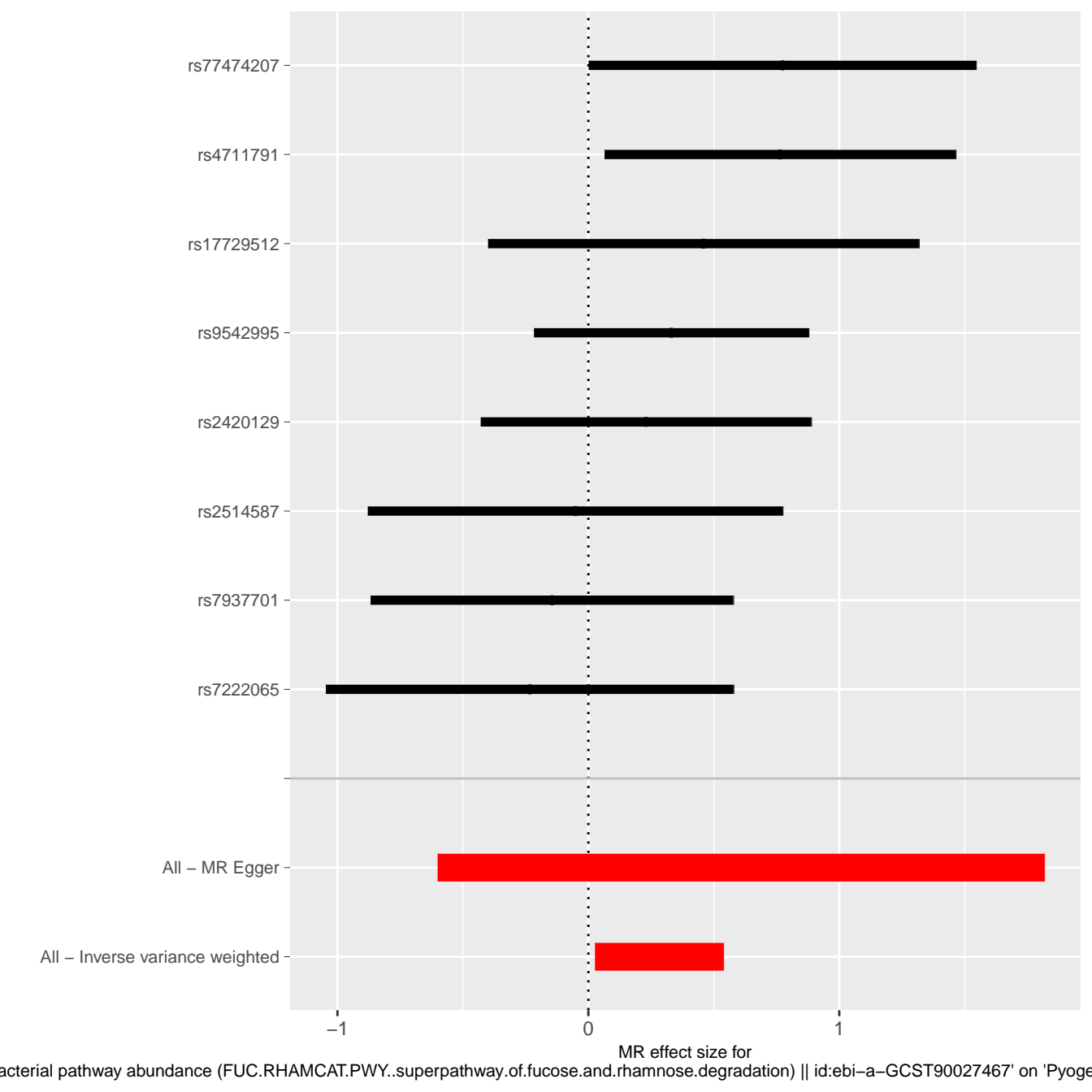

Supplement: Supplementary file 1 [file DataSheet1.zip › mendelian test/ebi-a-GCST90027467.csv_forest.pdf]

# MR Method

- Inverse variance weighted
- MR Egger

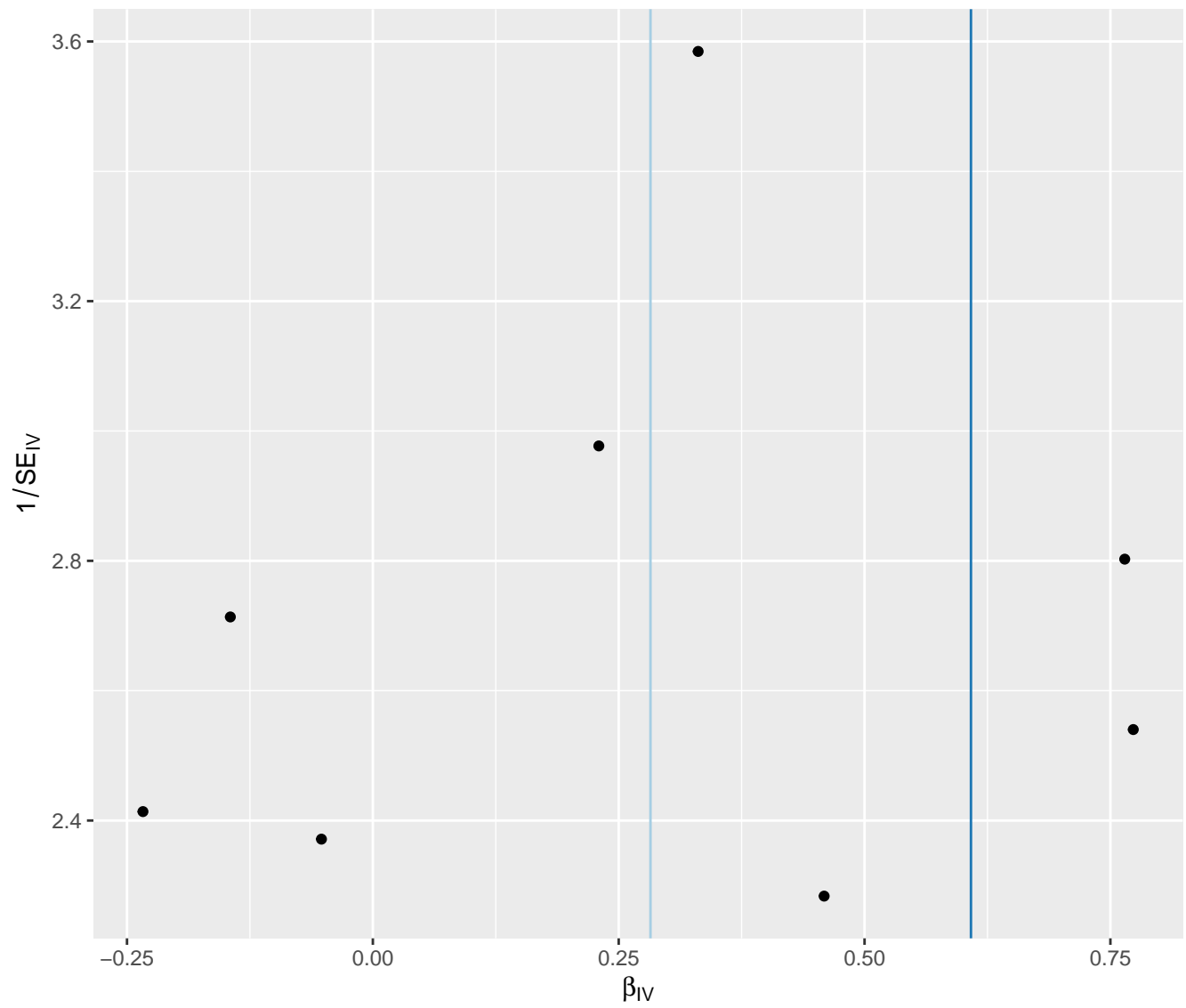

Supplement: Supplementary file 1 [file DataSheet1.zip › mendelian test/ebi-a-GCST90027467.csv_funnel.pdf]

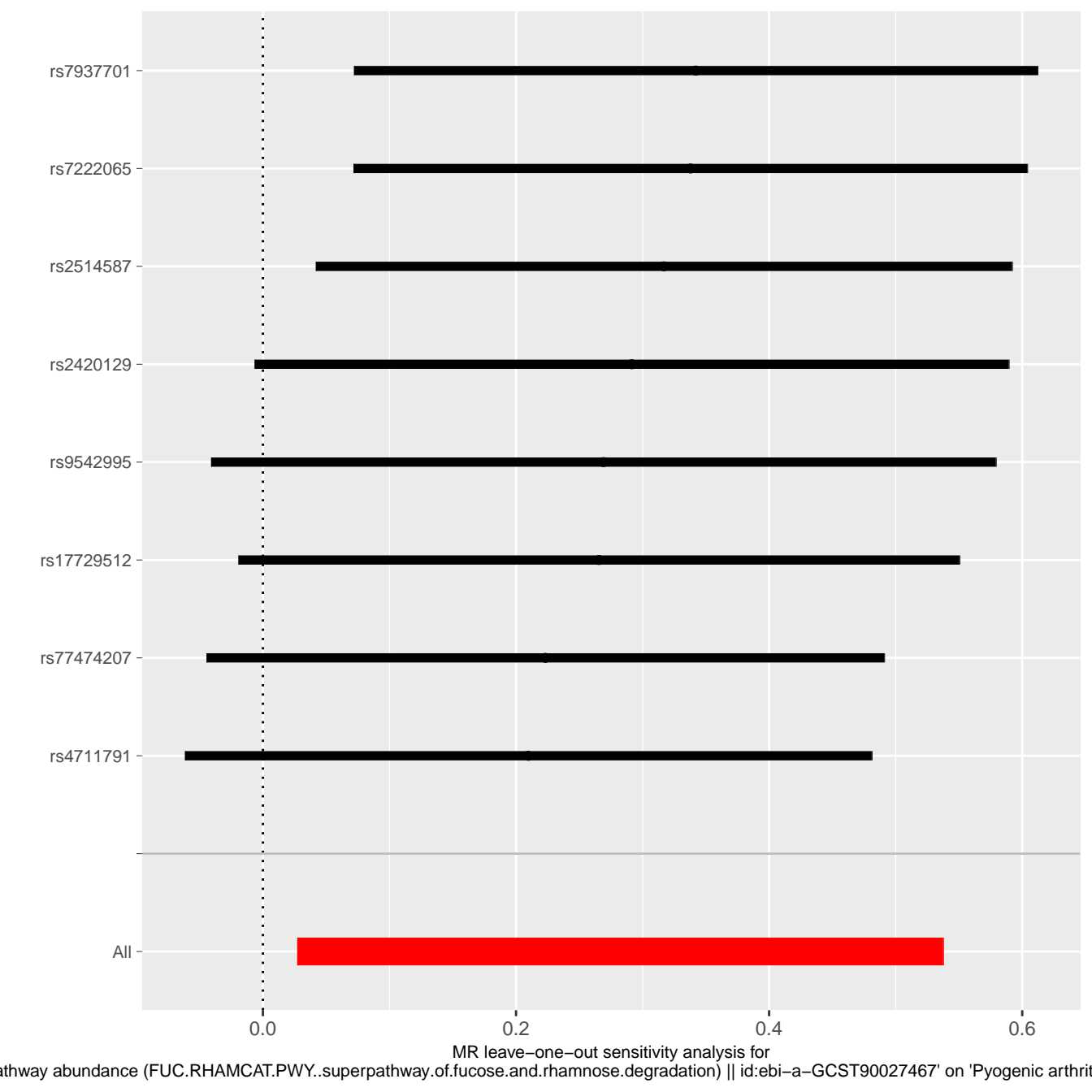

Supplement: Supplementary file 1 [file DataSheet1.zip › mendelian test/ebi-a-GCST90027467.csv_leave_one_out.pdf]

# MR Test

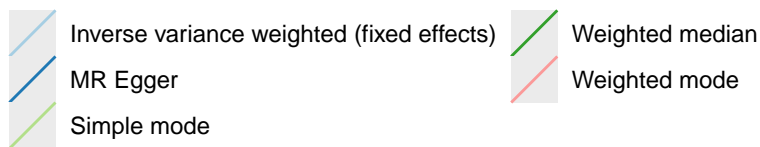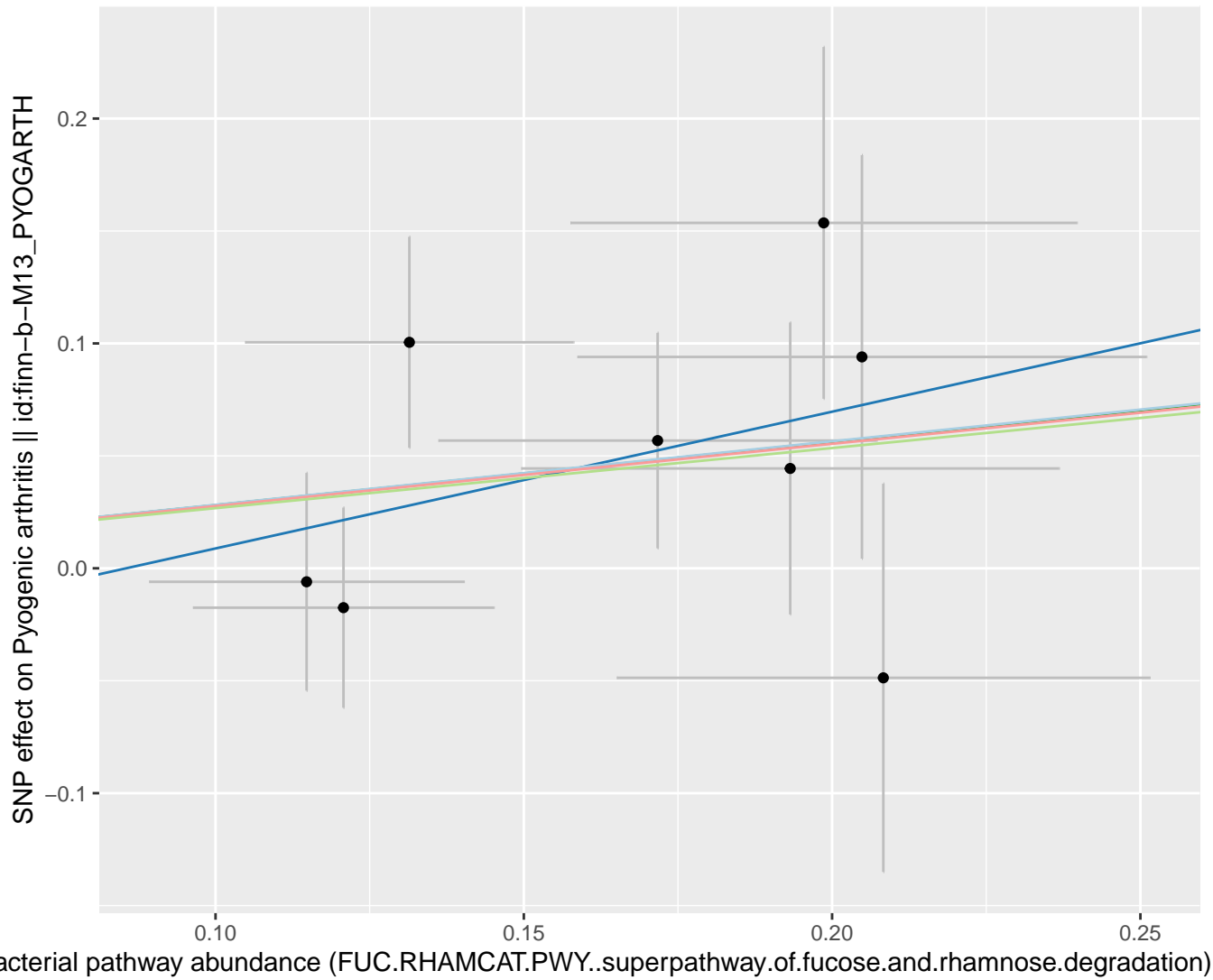

Supplement: Supplementary file 1 [file DataSheet1.zip › mendelian test/ebi-a-GCST90027467.csv_scatter.pdf]

# MR Method

- Inverse variance weighted
- MR Egger

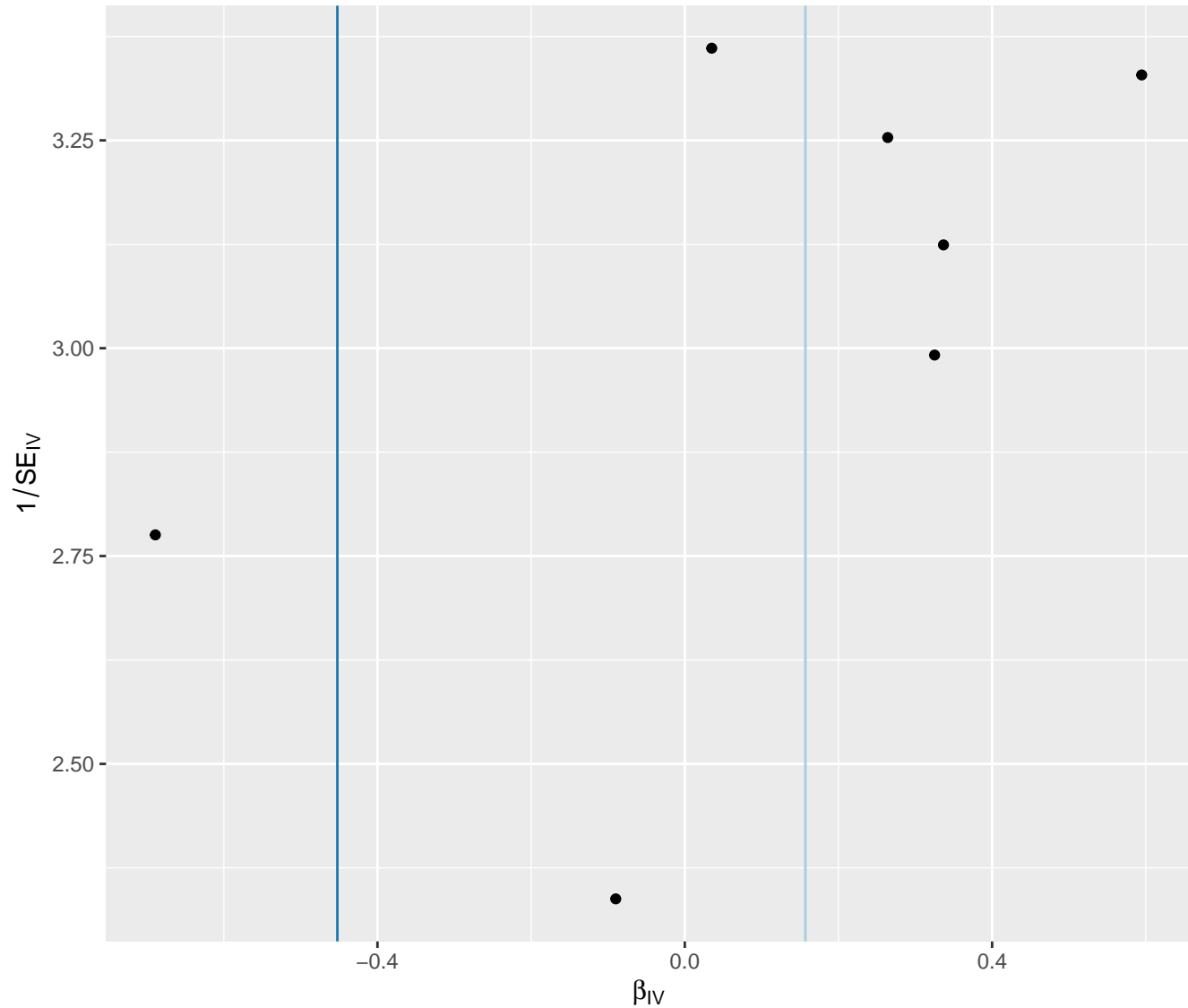

Supplement: Supplementary file 1 [file DataSheet1.zip › mendelian test/ebi-a-GCST90027468.csv_funnel.pdf]

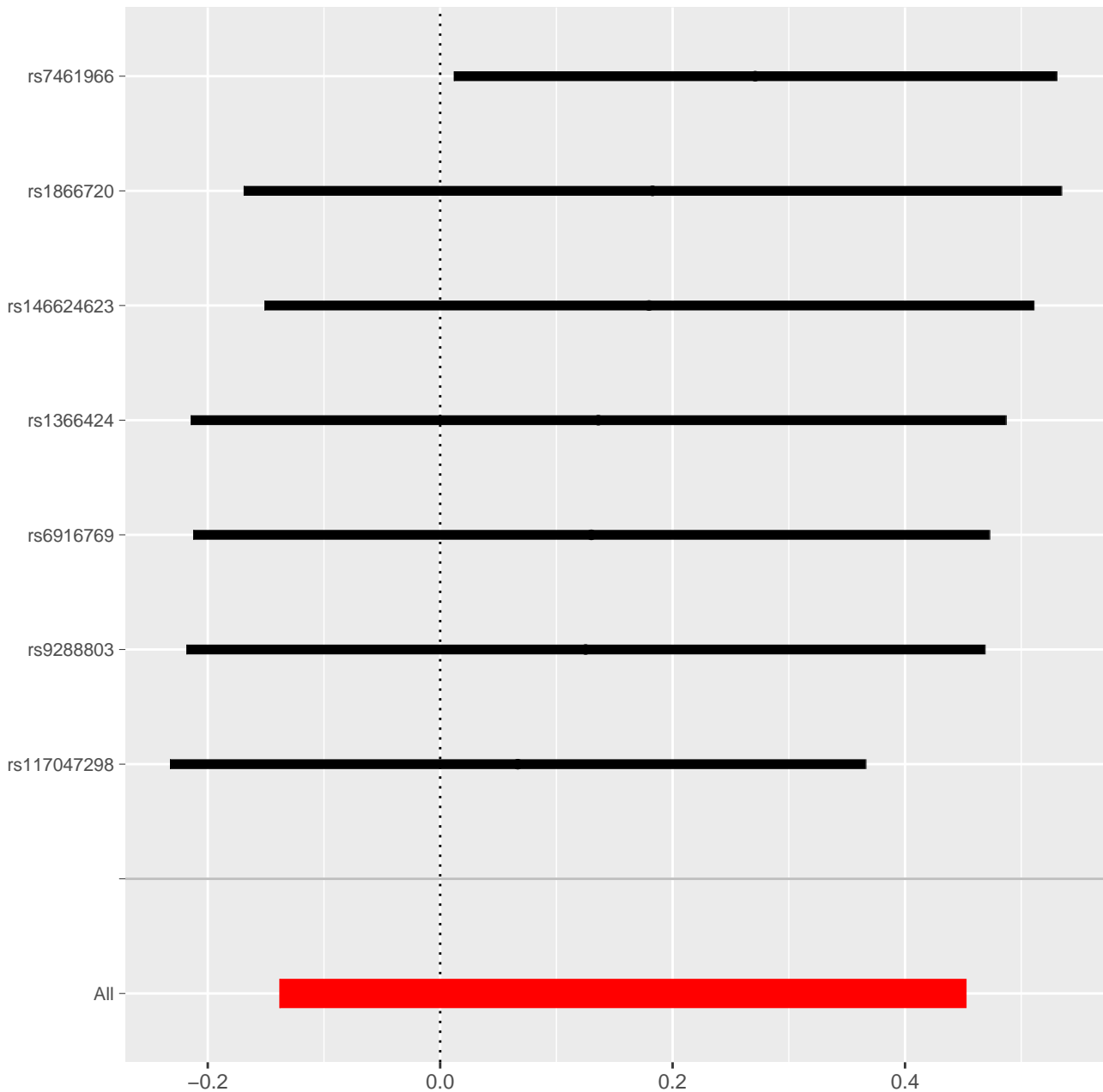

Supplement: Supplementary file 1 [file DataSheet1.zip › mendelian test/ebi-a-GCST90027468.csv_leave_one_out.pdf]

# MR Test

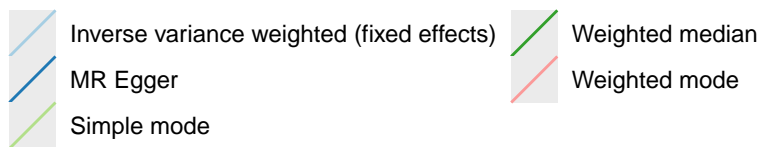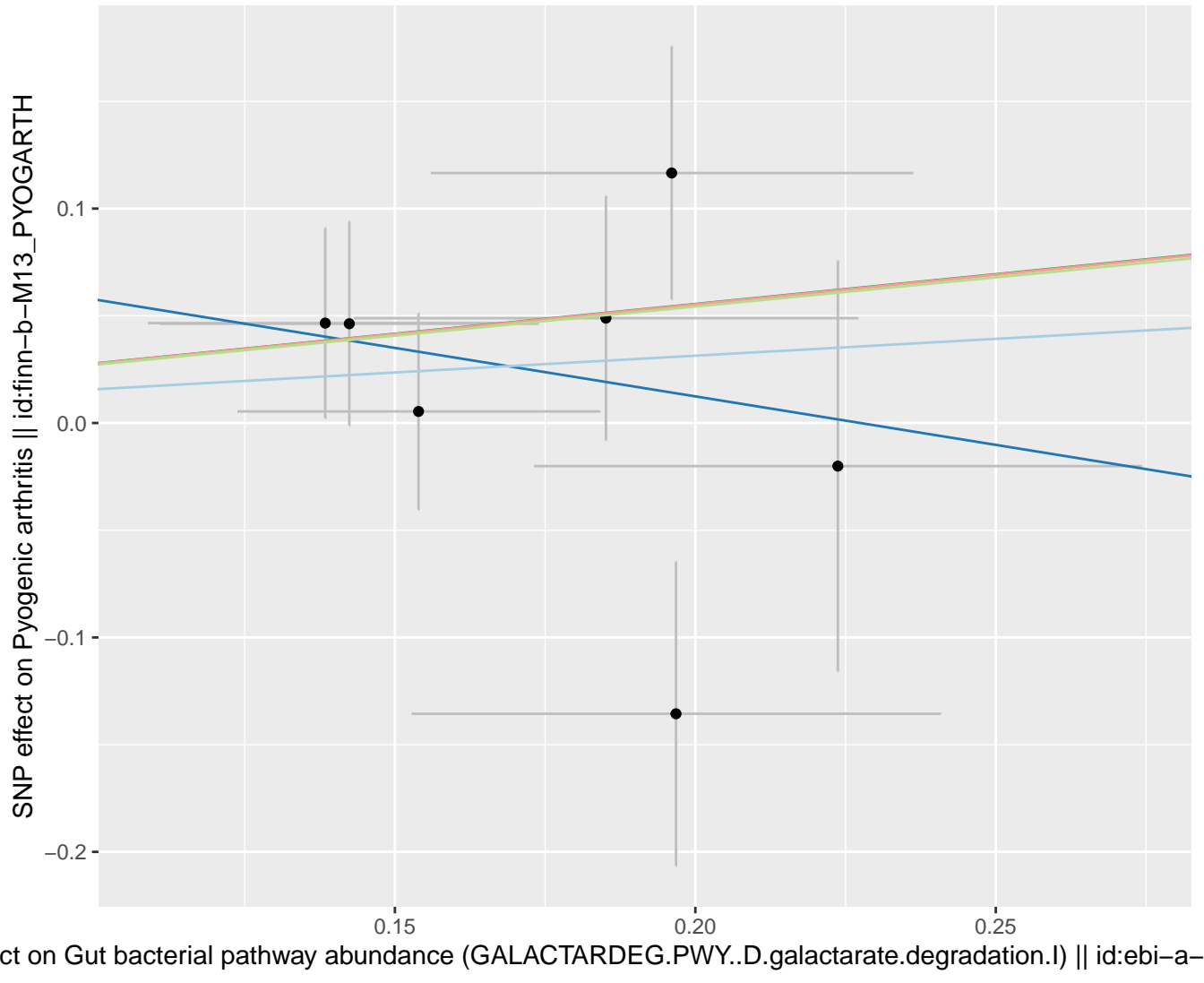

Supplement: Supplementary file 1 [file DataSheet1.zip › mendelian test/ebi-a-GCST90027468.csv_scatter.pdf]

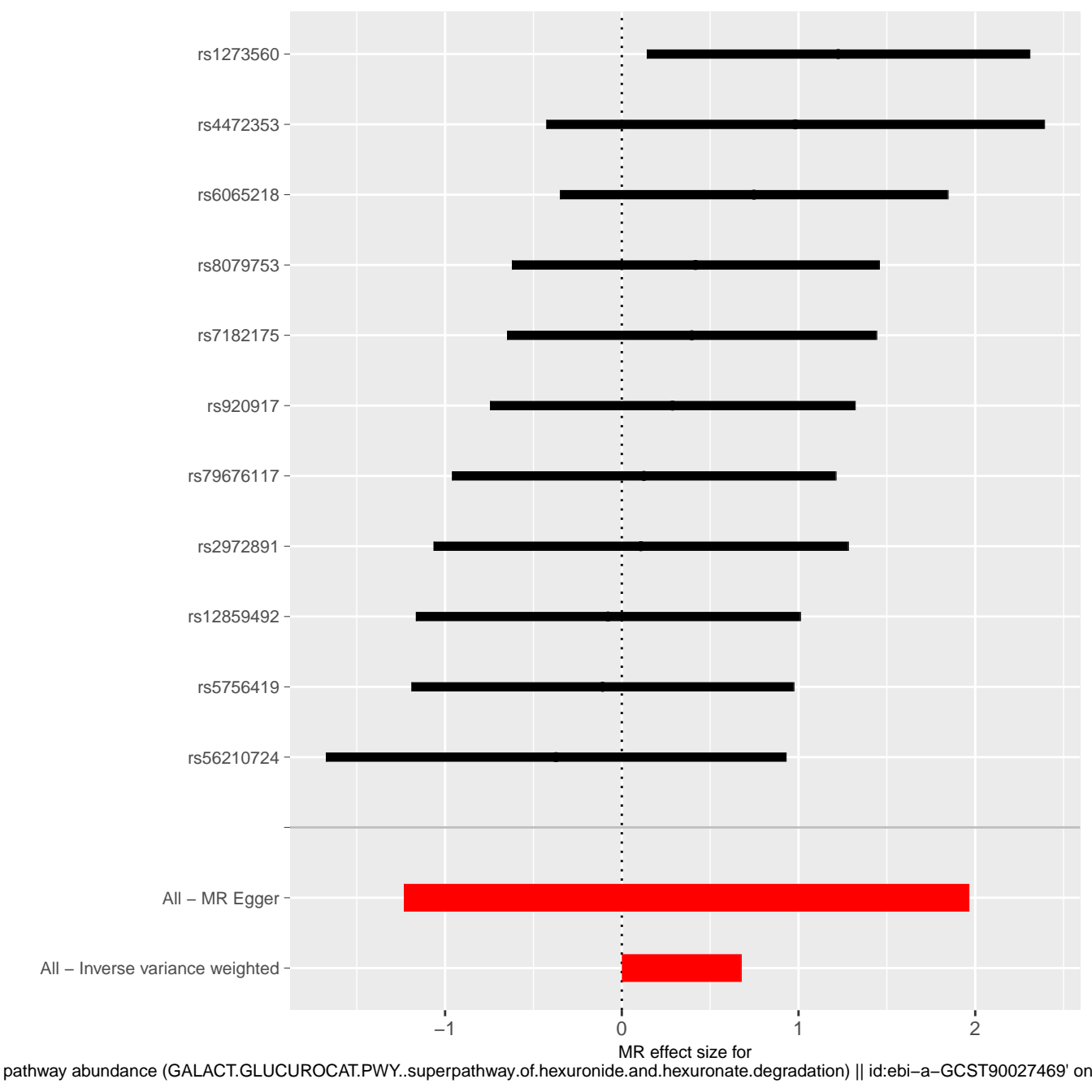

Supplement: Supplementary file 1 [file DataSheet1.zip › mendelian test/ebi-a-GCST90027469.csv_forest.pdf]

# MR Method

- Inverse variance weighted
- MR Egger

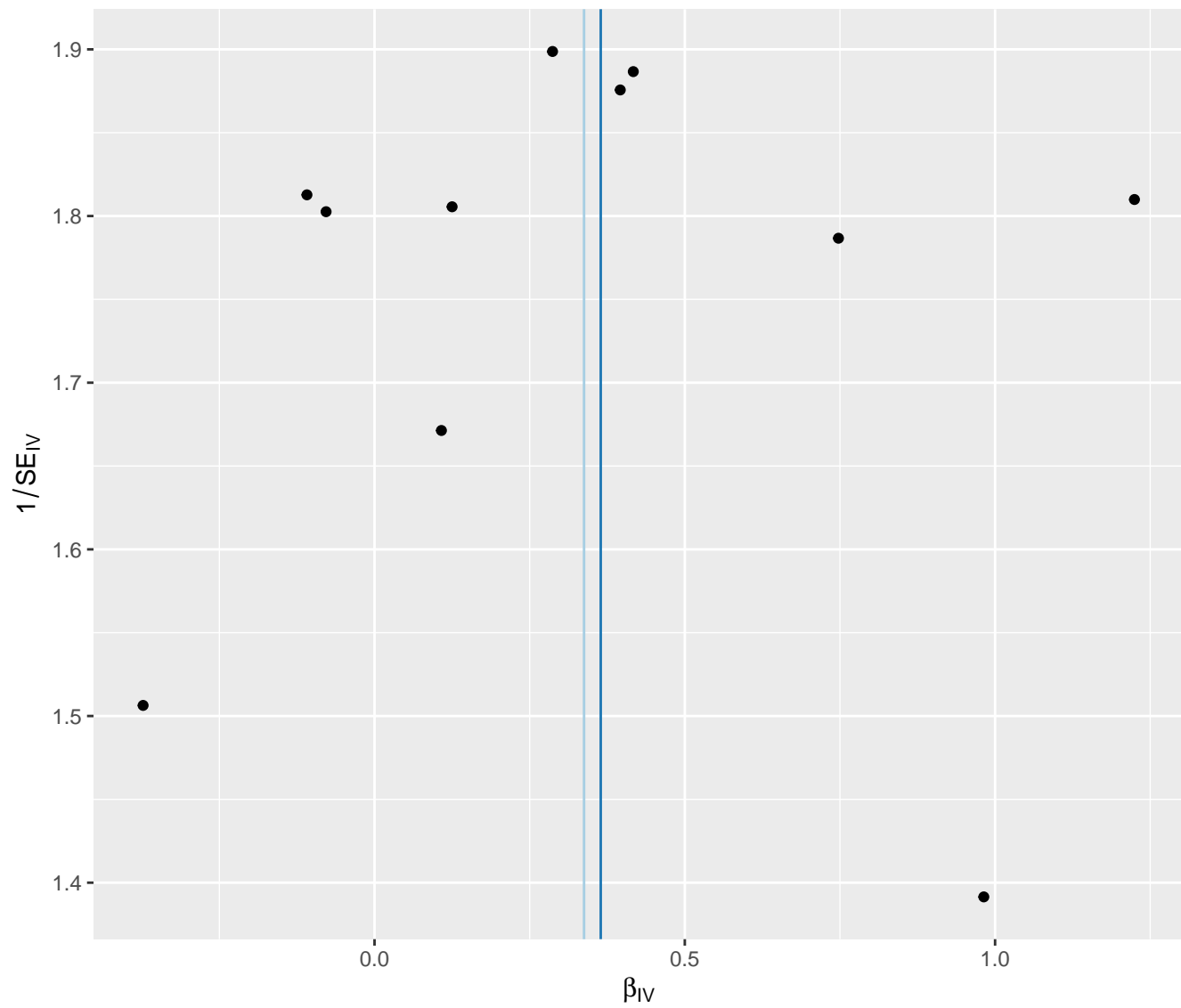

Supplement: Supplementary file 1 [file DataSheet1.zip › mendelian test/ebi-a-GCST90027469.csv_funnel.pdf]

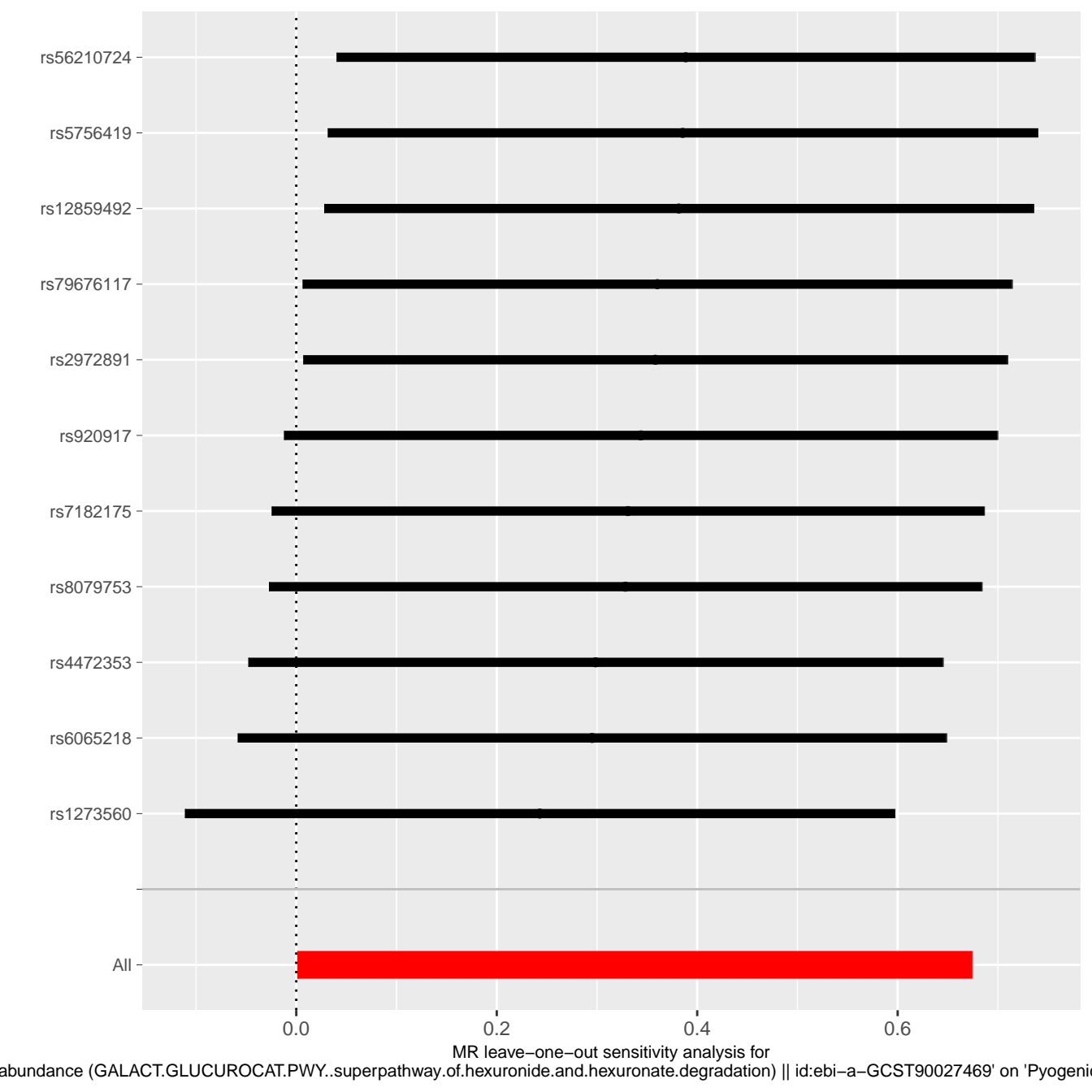

Supplement: Supplementary file 1 [file DataSheet1.zip › mendelian test/ebi-a-GCST90027469.csv_leave_one_out.pdf]

# MR Test

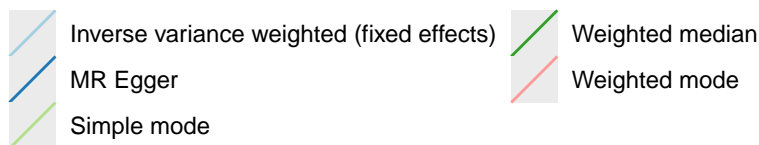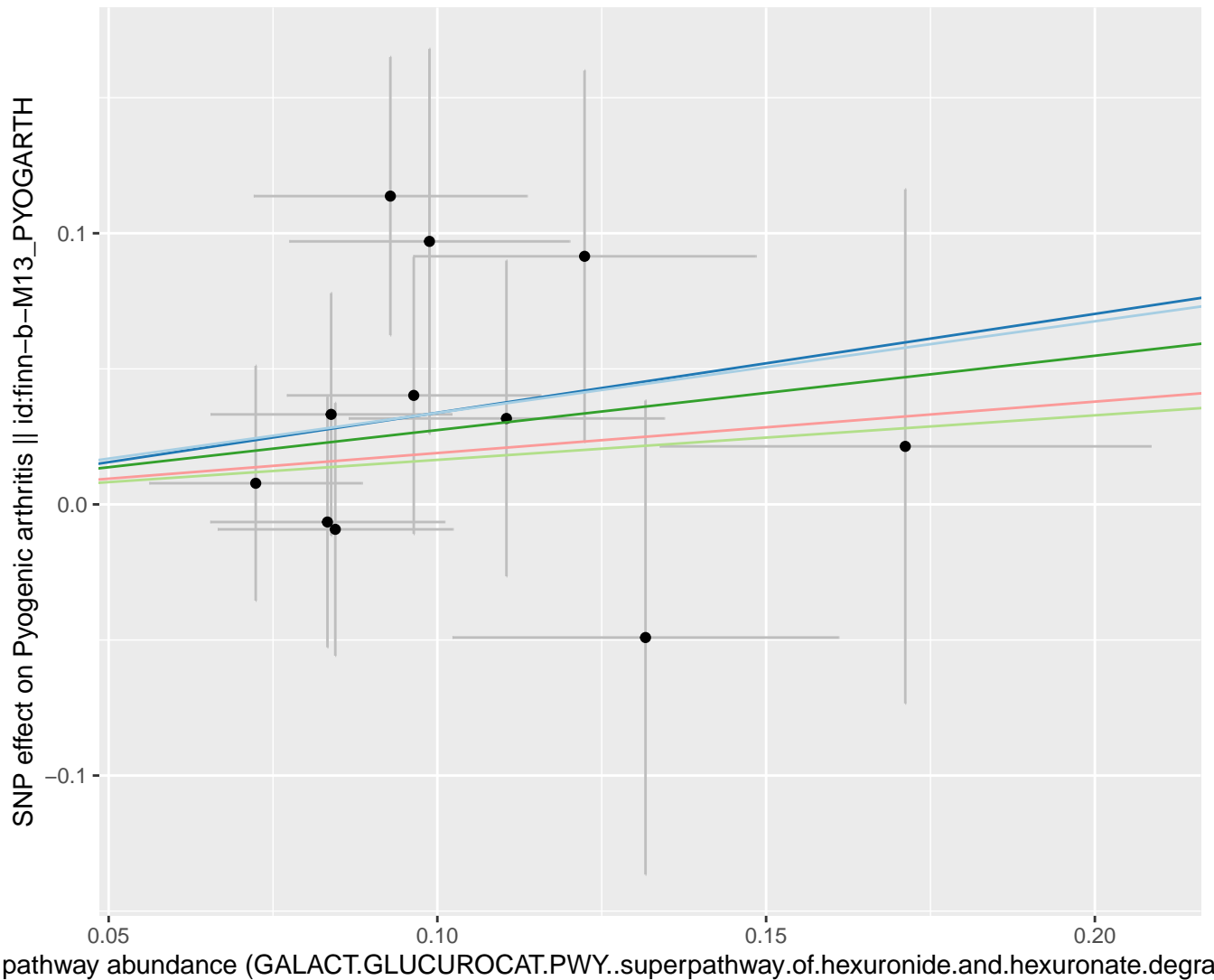

Supplement: Supplementary file 1 [file DataSheet1.zip › mendelian test/ebi-a-GCST90027469.csv_scatter.pdf]

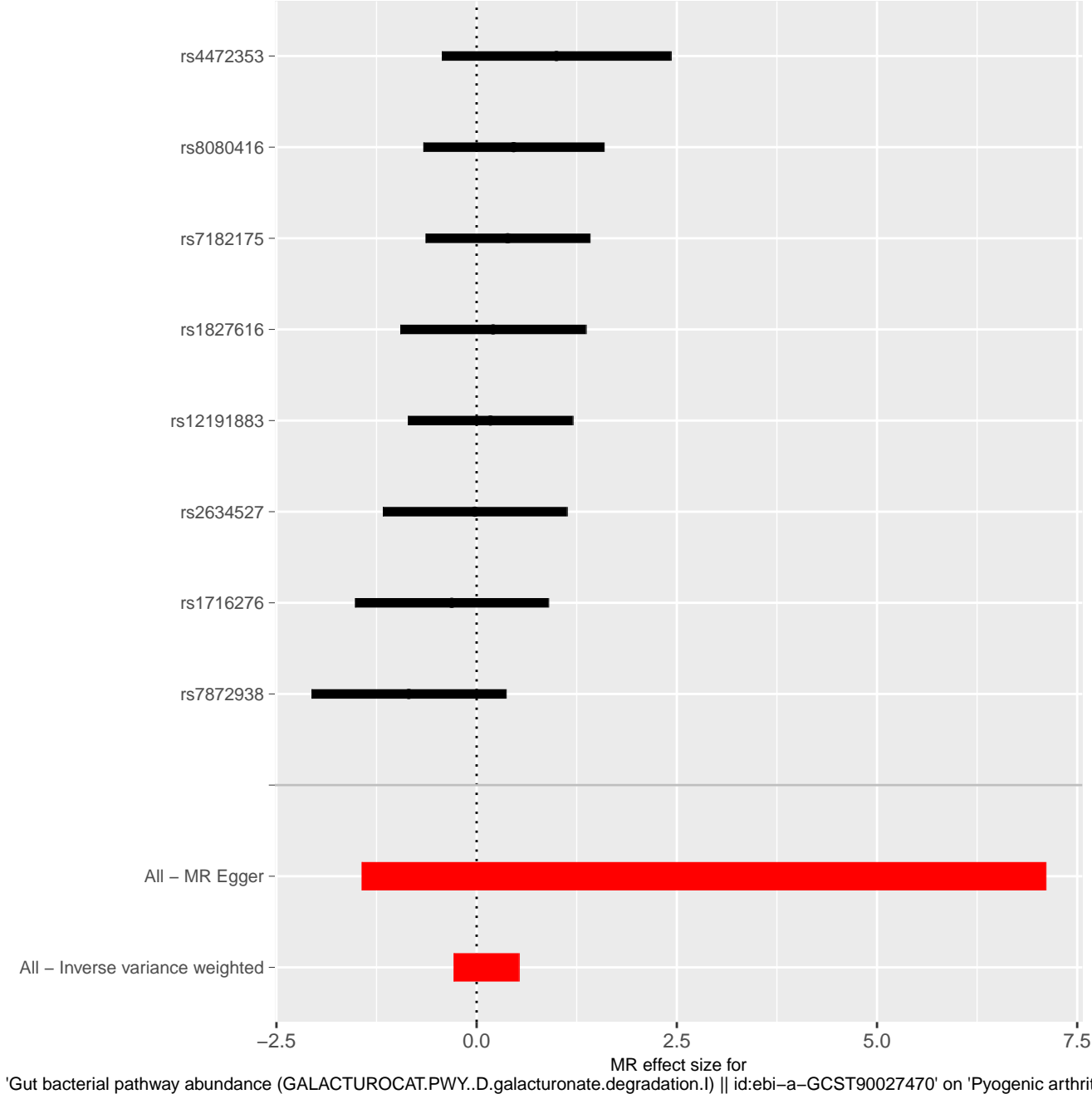

Supplement: Supplementary file 1 [file DataSheet1.zip › mendelian test/ebi-a-GCST90027470.csv_forest.pdf]

# MR Method

- Inverse variance weighted
- MR Egger

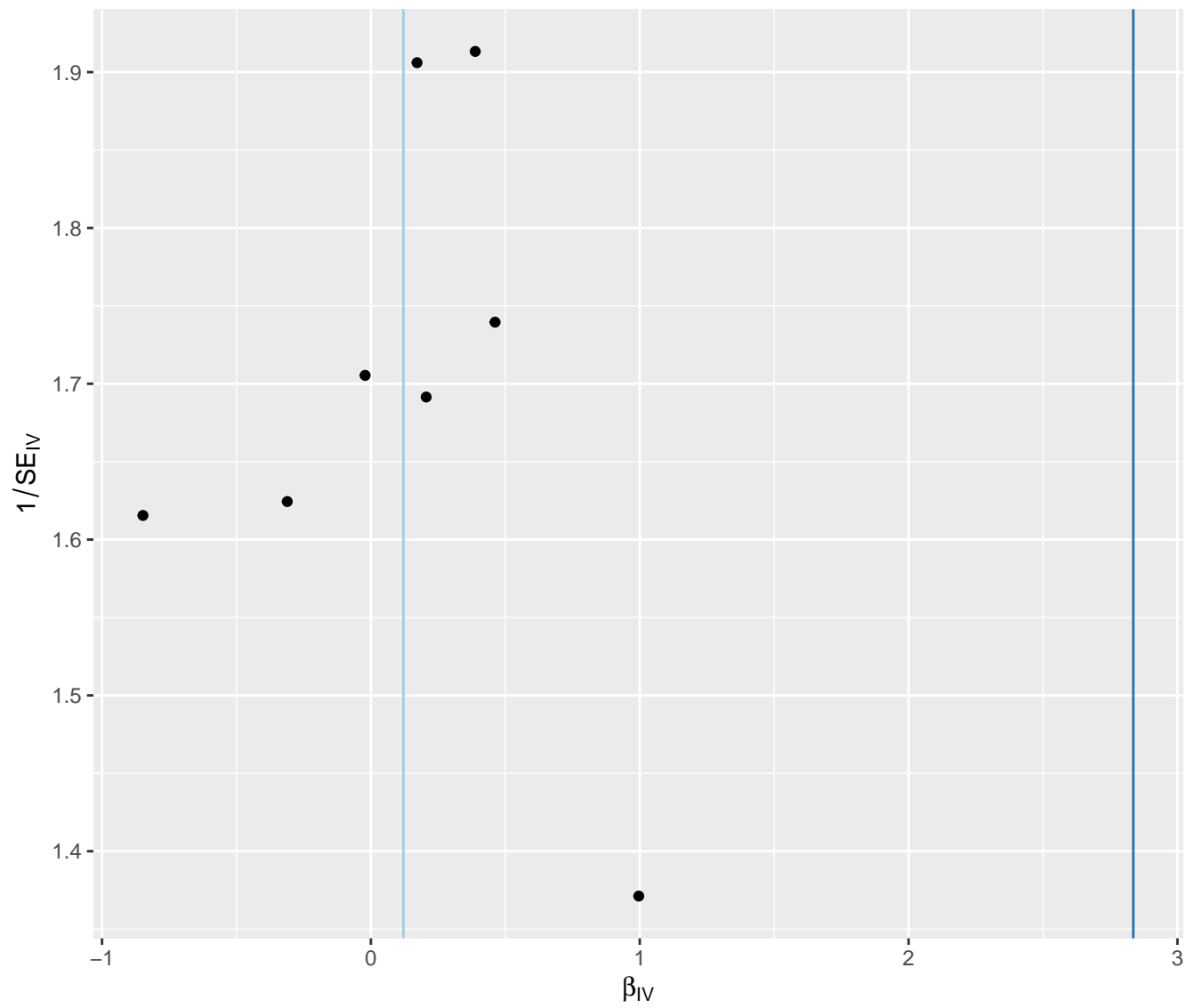

Supplement: Supplementary file 1 [file DataSheet1.zip › mendelian test/ebi-a-GCST90027470.csv_funnel.pdf]

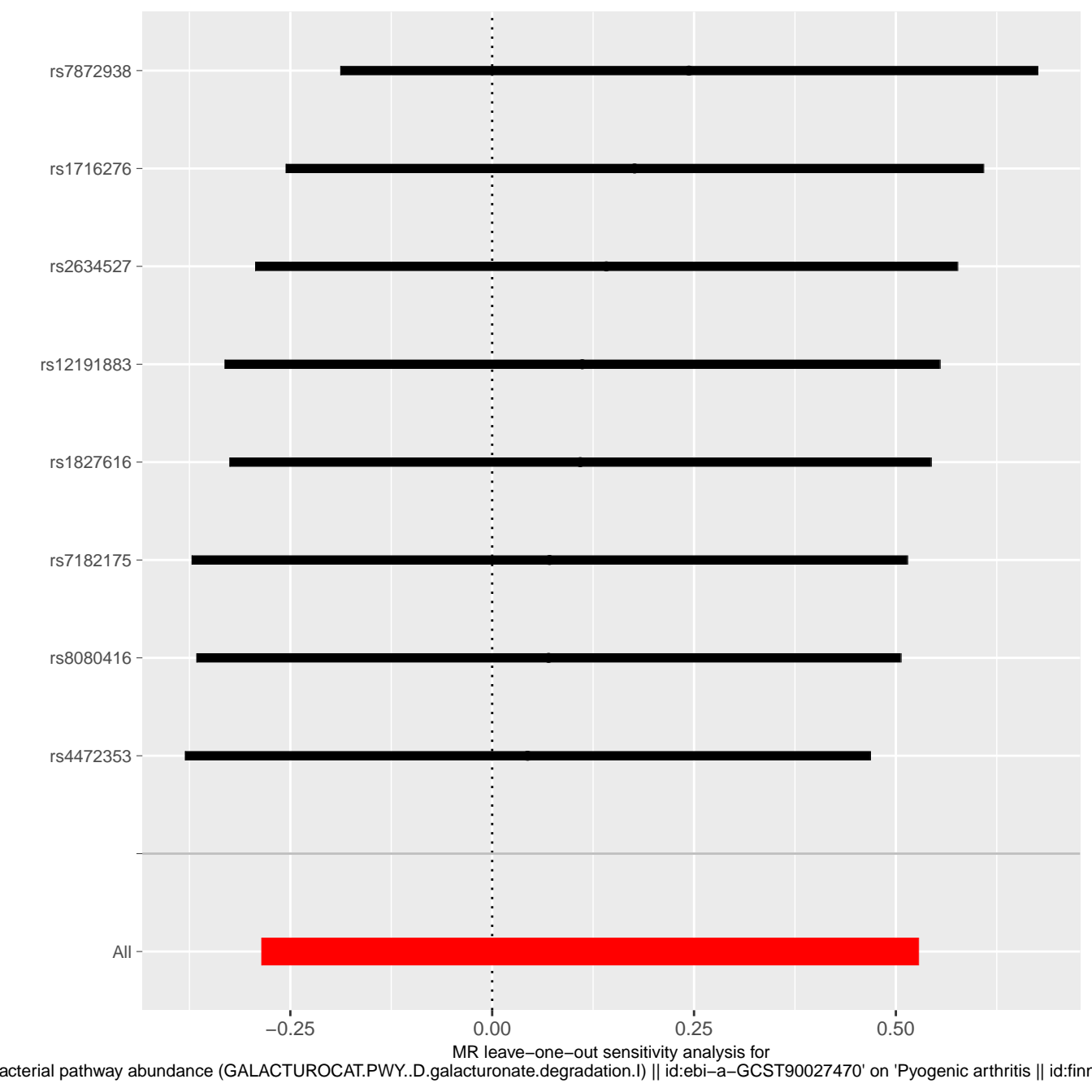

Supplement: Supplementary file 1 [file DataSheet1.zip › mendelian test/ebi-a-GCST90027470.csv_leave_one_out.pdf]

# MR Test

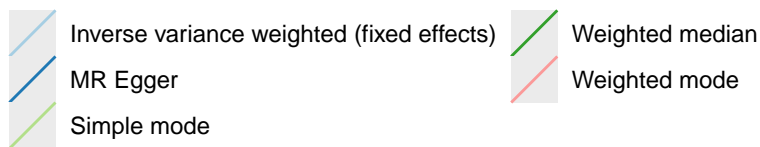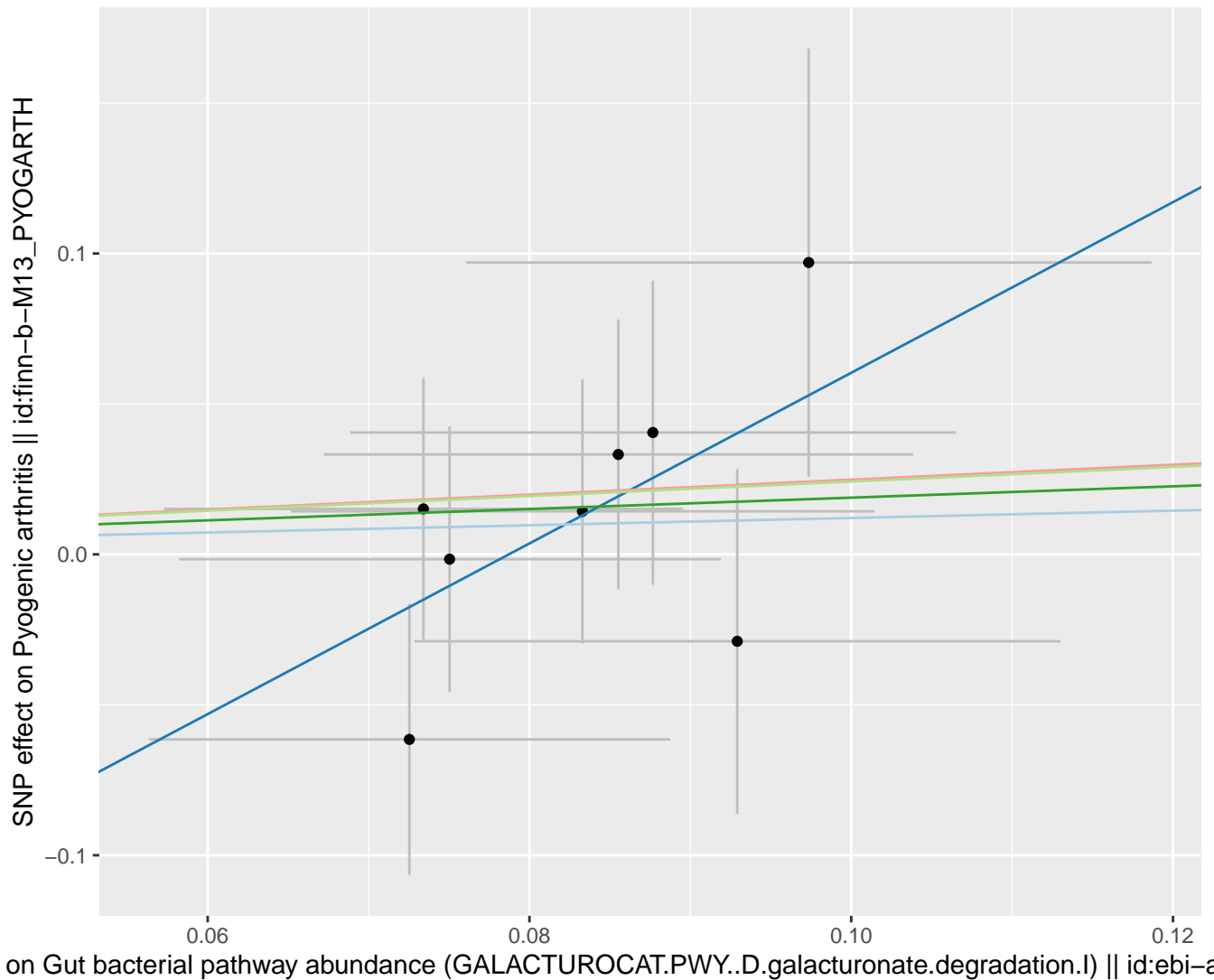

Supplement: Supplementary file 1 [file DataSheet1.zip › mendelian test/ebi-a-GCST90027470.csv_scatter.pdf]

# MR Method

- Inverse variance weighted
- MR Egger

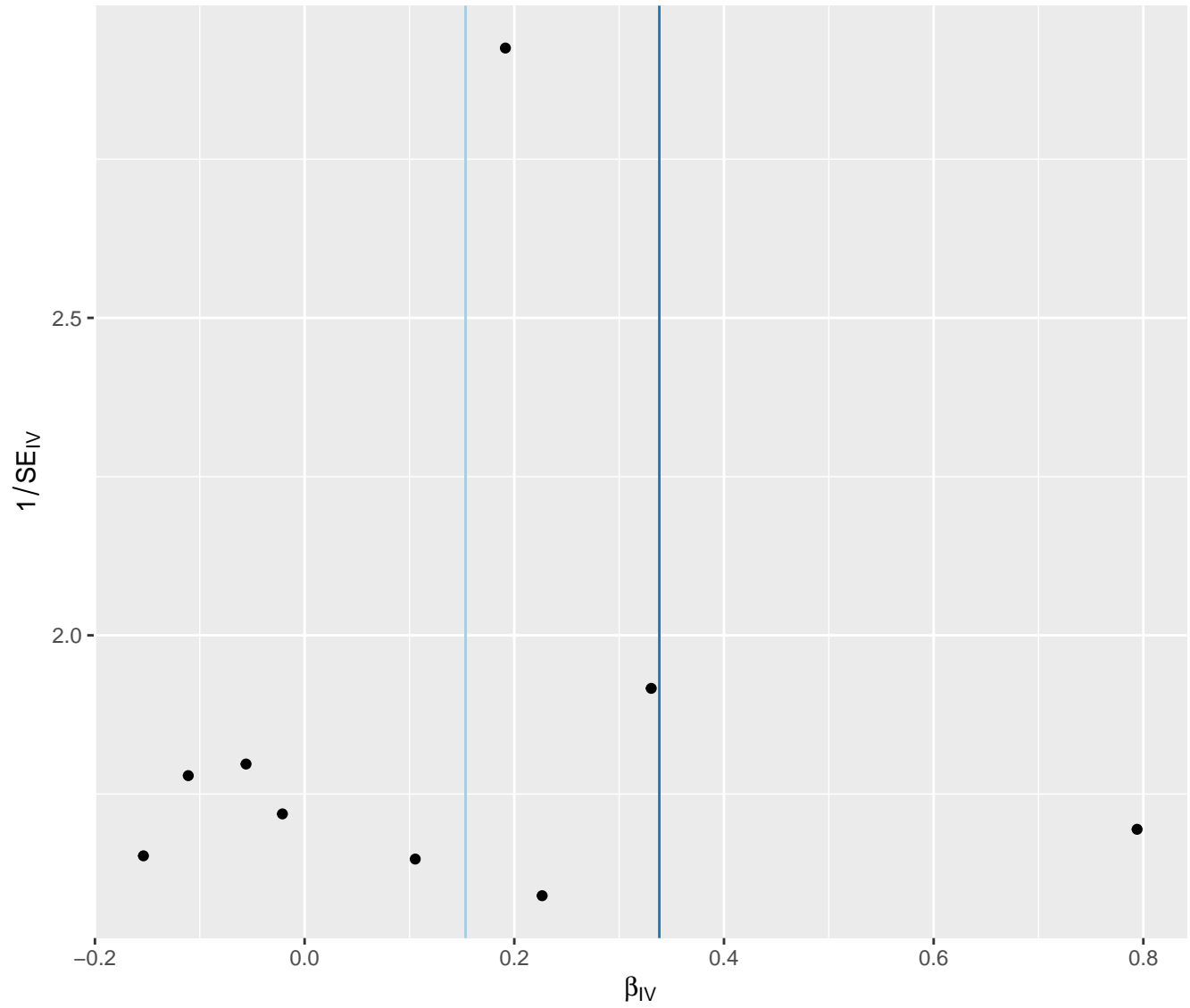

Supplement: Supplementary file 1 [file DataSheet1.zip › mendelian test/ebi-a-GCST90027471.csv_funnel.pdf]

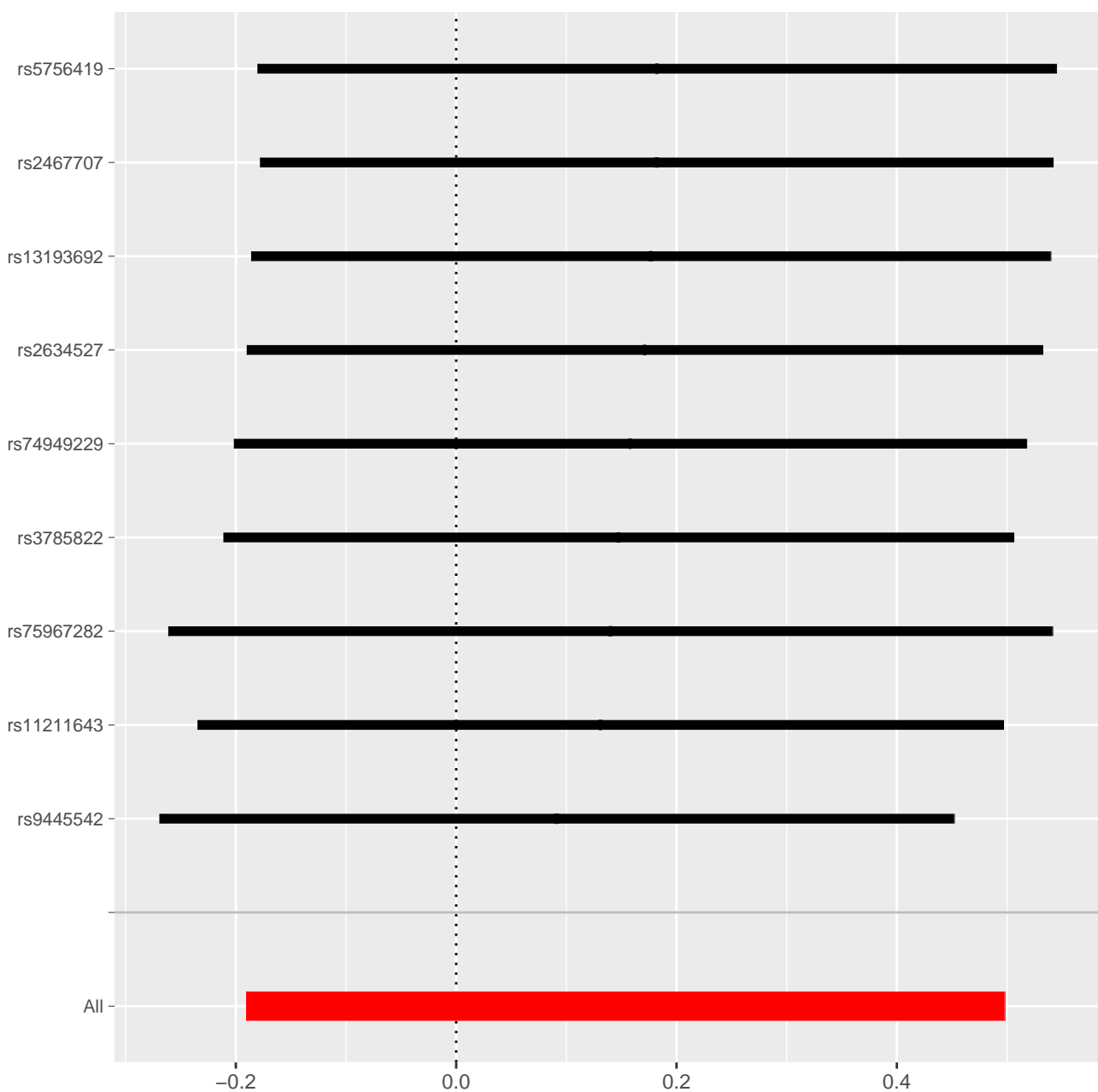

Supplement: Supplementary file 1 [file DataSheet1.zip › mendelian test/ebi-a-GCST90027471.csv_leave_one_out.pdf]

# MR Test

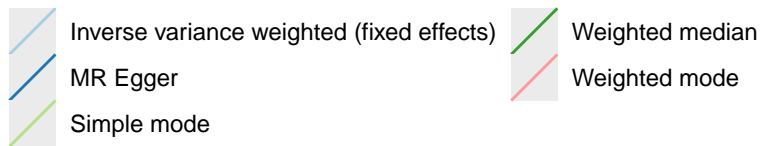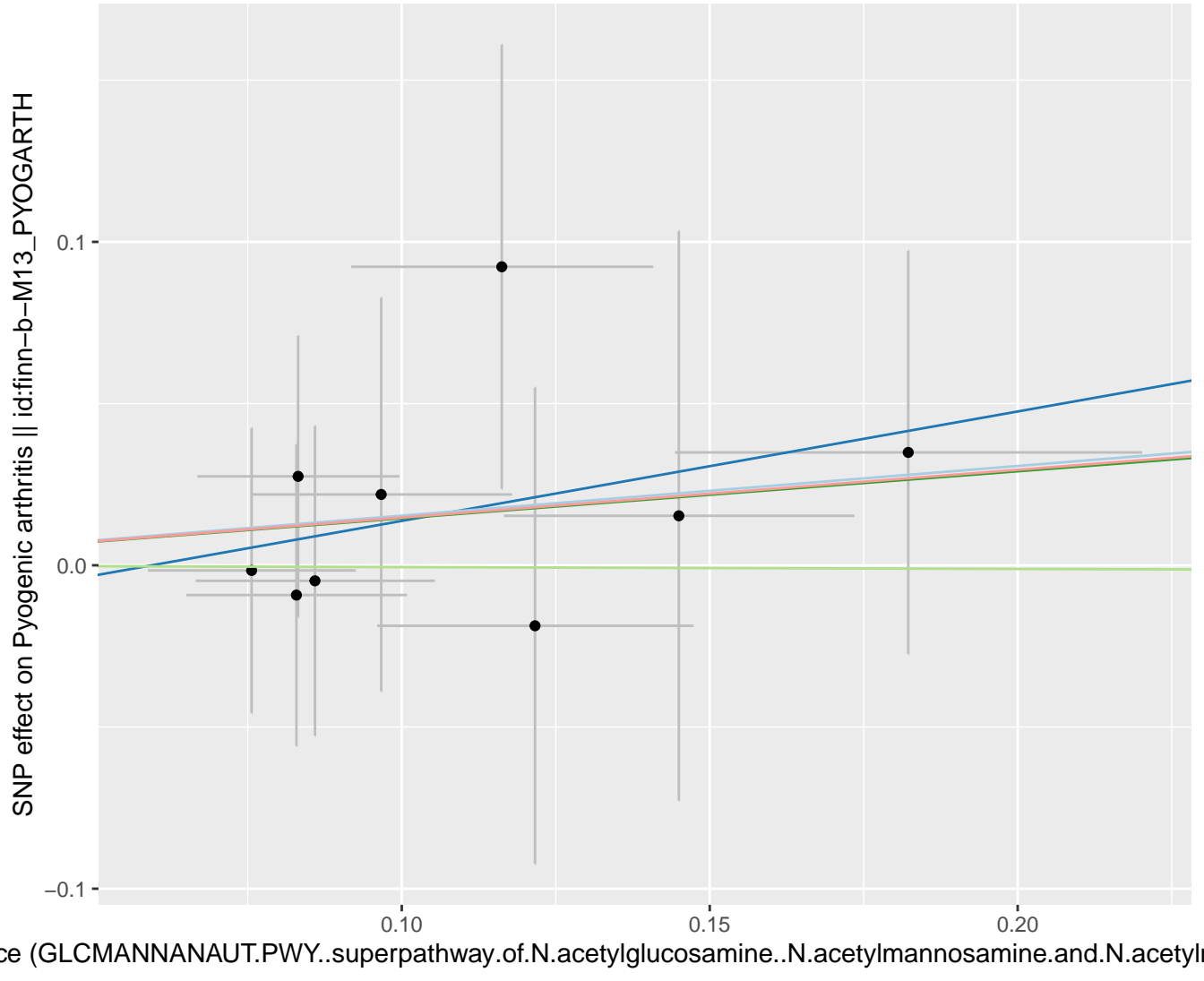

Supplement: Supplementary file 1 [file DataSheet1.zip › mendelian test/ebi-a-GCST90027471.csv_scatter.pdf]

# MR Method

- Inverse variance weighted
- MR Egger

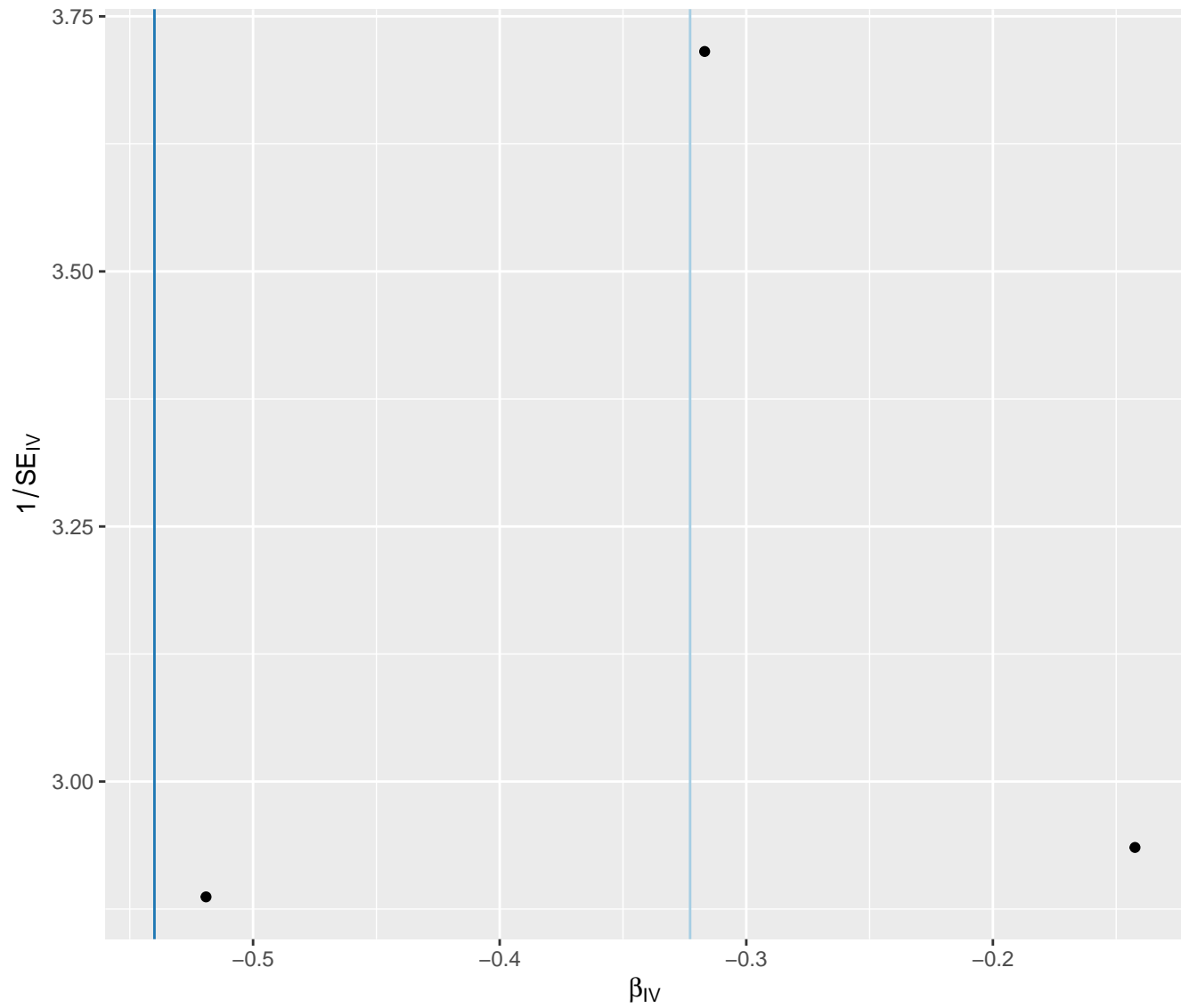

Supplement: Supplementary file 1 [file DataSheet1.zip › mendelian test/ebi-a-GCST90027472.csv_funnel.pdf]

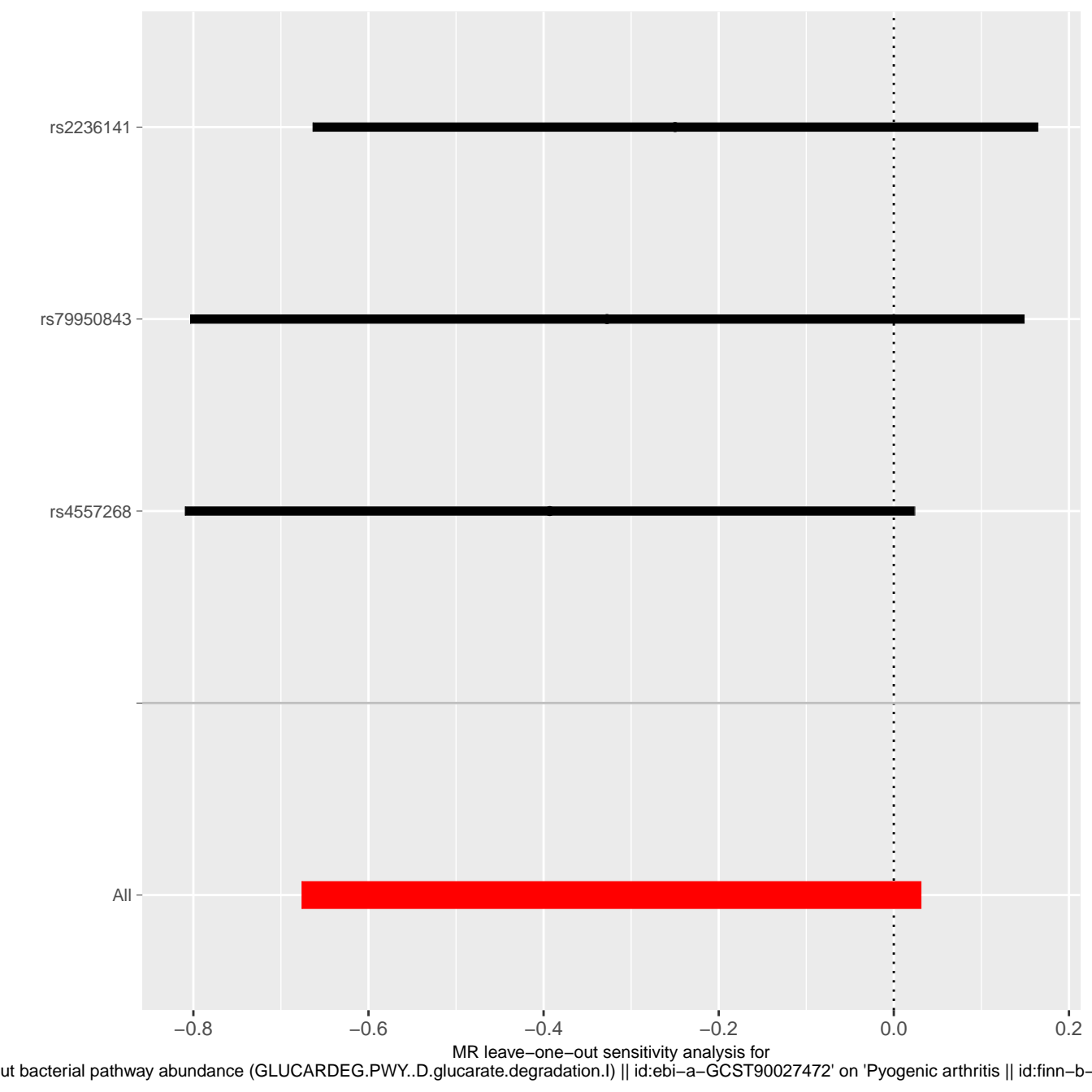

Supplement: Supplementary file 1 [file DataSheet1.zip › mendelian test/ebi-a-GCST90027472.csv_leave_one_out.pdf]

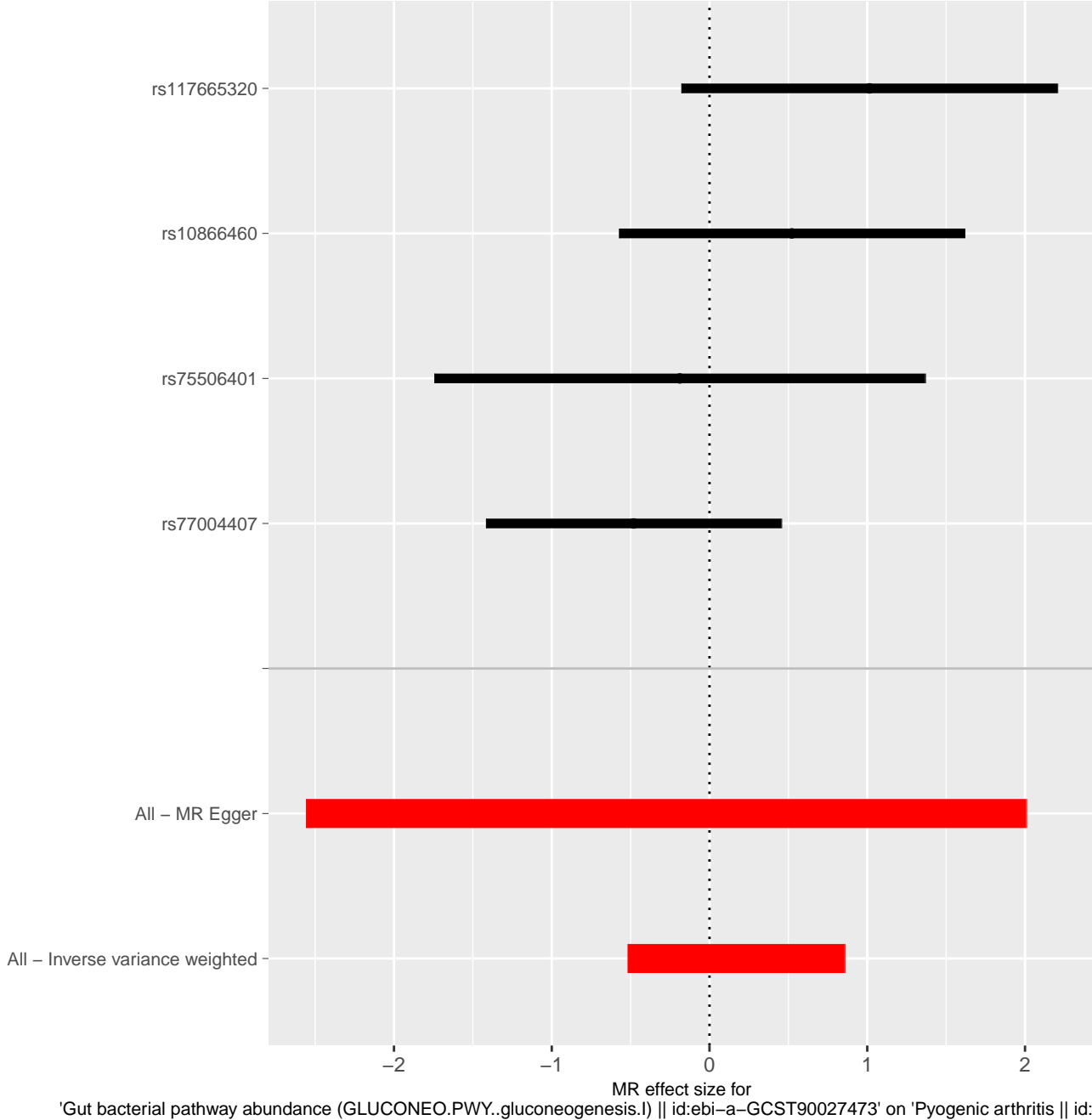

Supplement: Supplementary file 1 [file DataSheet1.zip › mendelian test/ebi-a-GCST90027473.csv_forest.pdf]

# MR Method

- Inverse variance weighted
- MR Egger

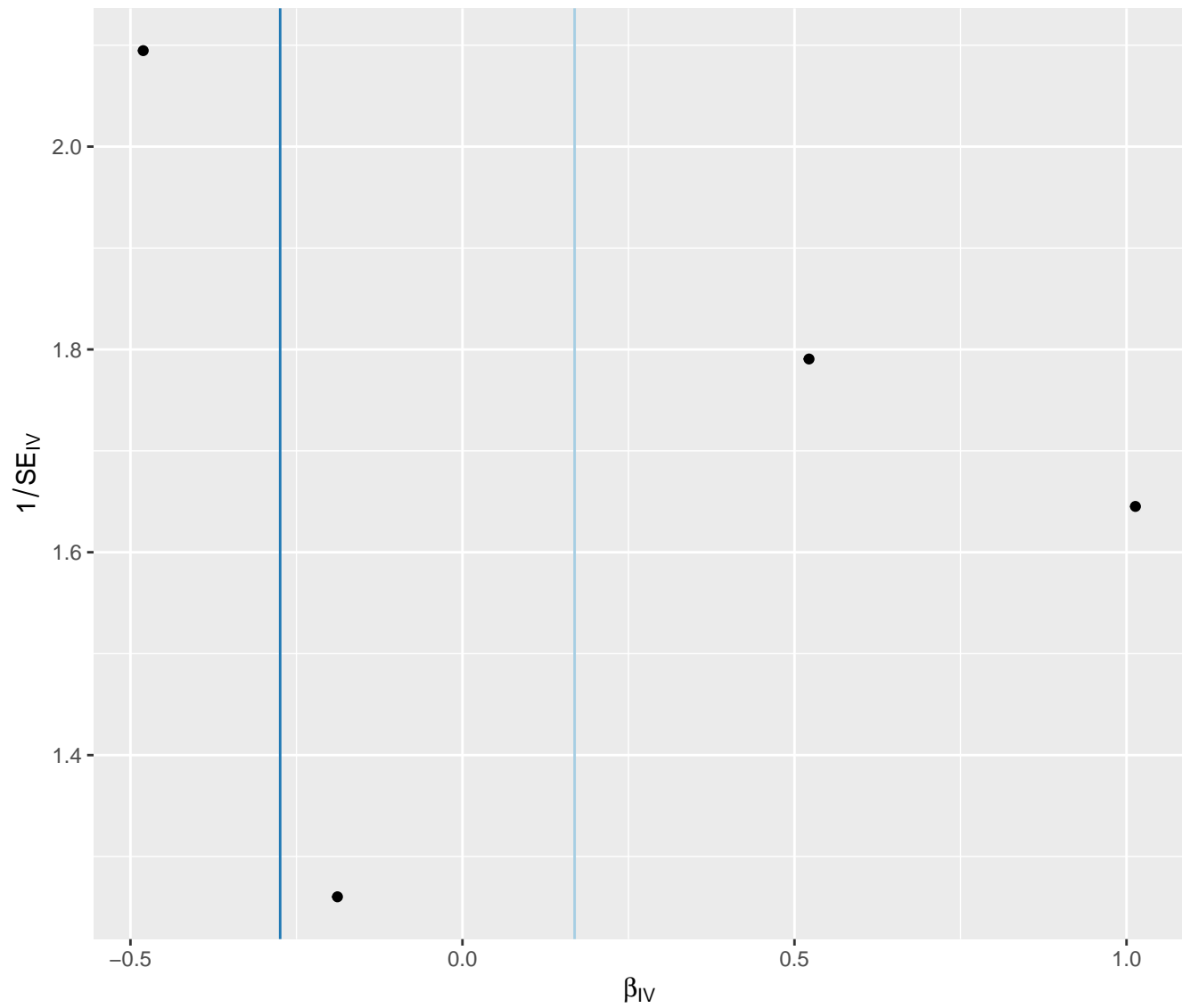

Supplement: Supplementary file 1 [file DataSheet1.zip › mendelian test/ebi-a-GCST90027473.csv_funnel.pdf]

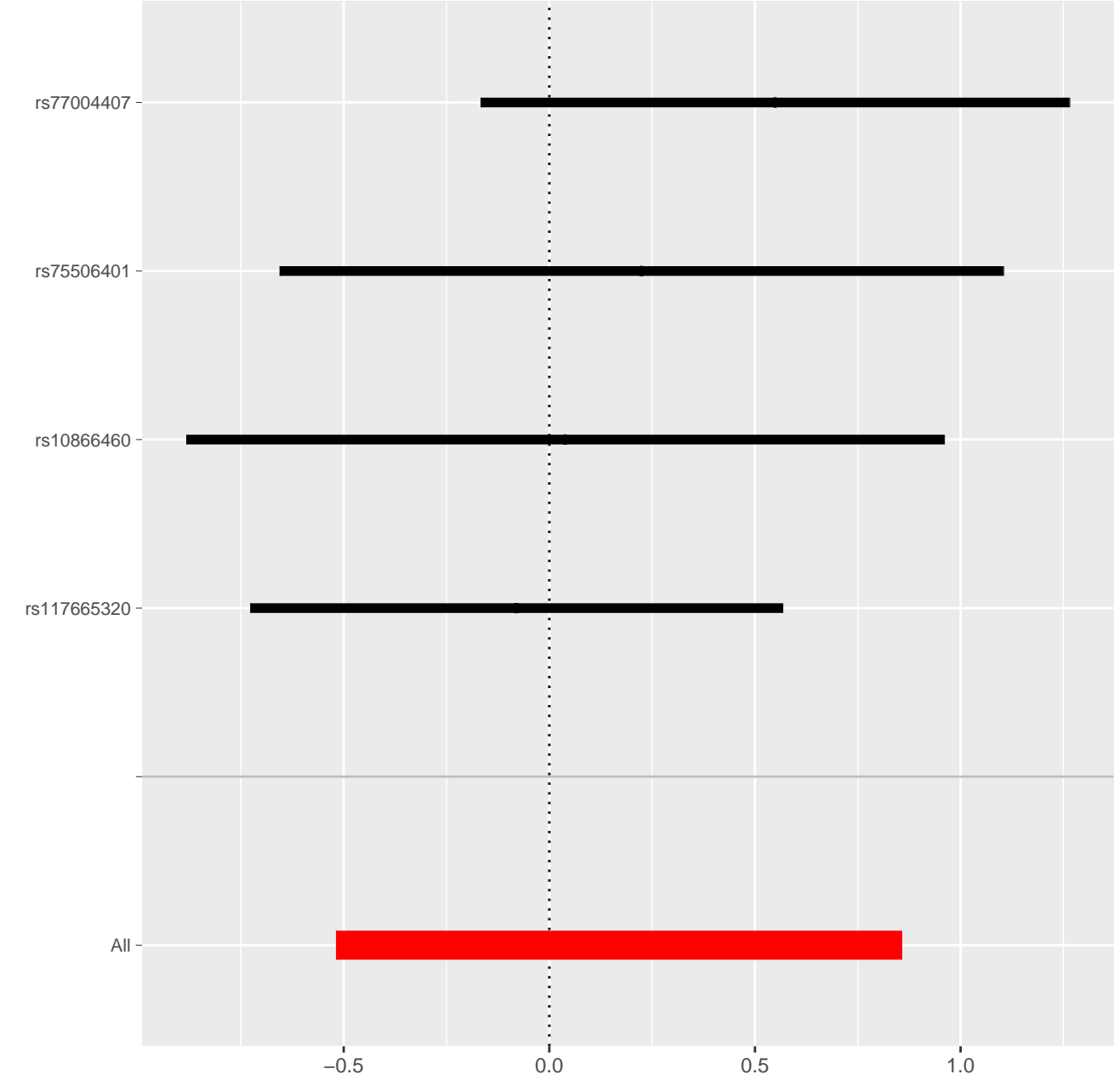

Supplement: Supplementary file 1 [file DataSheet1.zip › mendelian test/ebi-a-GCST90027473.csv_leave_one_out.pdf]

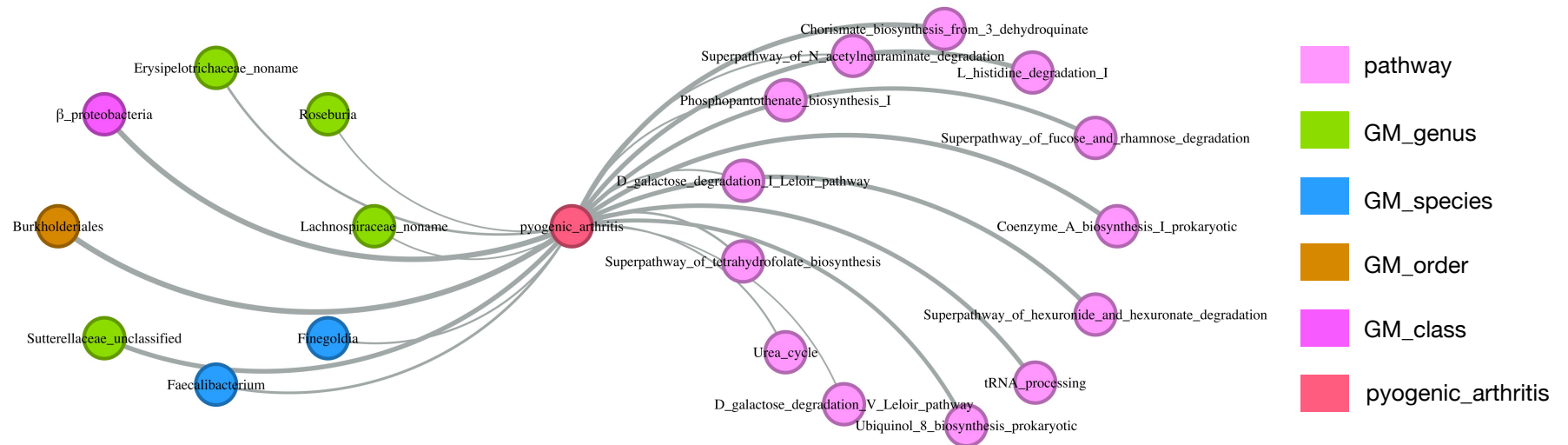

Fig.S1 The causal relationship between GMs and metabolic pathways and PA

Supplement: Supplementary file 3 [file DataSheet3.pdf]
